# The genus *Pseudovibrio* contains metabolically versatile bacteria adapted for symbiosis

**DOI:** 10.1111/1462-2920.12123

**Published:** 2013-04-18

**Authors:** Vladimir Bondarev, Michael Richter, Stefano Romano, Jörn Piel, Anne Schwedt, Heide N Schulz-Vogt

**Affiliations:** 1 Max Planck Institute for Marine Microbiology Ecophysiology GroupCelsiusstr. 1, 28359, Bremen, Germany; 2 Max Planck Institute for Marine Microbiology Microbial Genomics and Bioinformatics Research GroupCelsiusstr. 1, 28359, Bremen, Germany; 3 University of Bonn Kekulé-Institute of Organic Chemistry and BiochemistryGerhard-Domagk Str. 1, 53121, Bonn, Germany; 4 Leibniz-Institute for Baltic Sea Research Warnemuende (IOW) Section Biological OceanographySeestrasse 15, 18119, Rostock, Germany; 5 ETH Zurich Institute of MicrobiologyWolfgang-Pauli-Str. 10, 8093, Zurich, Switzerland

## Abstract

The majority of strains belonging to the genus *Pseudovibrio* have been isolated from marine invertebrates such as tunicates, corals and particularly sponges, but the physiology of these bacteria is poorly understood. In this study, we analyse for the first time the genomes of two *Pseudovibrio* strains – FO-BEG1 and JE062. The strain FO-BEG1 is a required symbiont of a cultivated *Beggiatoa* strain, a sulfide-oxidizing, autotrophic bacterium, which was initially isolated from a coral. Strain JE062 was isolated from a sponge. The presented data show that both strains are generalistic bacteria capable of importing and oxidizing a wide range of organic and inorganic compounds to meet their carbon, nitrogen, phosphorous and energy requirements under both, oxic and anoxic conditions. Several physiological traits encoded in the analysed genomes were verified in laboratory experiments with both isolates. Besides the versatile metabolic abilities of both *Pseudovibrio* strains, our study reveals a number of open reading frames and gene clusters in the genomes that seem to be involved in symbiont–host interactions. Both *Pseudovibrio* strains have the genomic potential to attach to host cells, interact with the eukaryotic cell machinery, produce secondary metabolites and supply the host with cofactors.

## Introduction

The first strain of the genus *Pseudovibrio*, *Pseudovibrio denitrificans*, was isolated from coastal seawater in 2004 and was described as a marine, heterotrophic, and facultatively anaerobic bacterium capable of denitrification and fermentation (Shieh *et al*., 2004). Three further type strains, *P. ascidiaceicola* (Fukunaga *et al*., 2006), *P. japonicus* (Hosoya and Yokota, 2007), and the recently described *P. axinellae* (O’Halloran *et al*., 2012) were subsequently isolated from a tunicate, coastal seawater, and a sponge respectively. Physiologically, these isolates were not notably different from *P. denitrificans*. Besides the four type strains, *Pseudovibrio*-related bacteria have been found in various studies throughout the world either by 16S rDNA analysis or direct isolation methods (Hentschel *et al*., 2001; Webster and Hill, 2001; Olson *et al*., 2002; Thakur *et al*., 2003; Thiel and Imhoff, 2003; Thoms *et al*., 2003; Agogué *et al*., 2005; Lafi *et al*., 2005; Enticknap *et al*., 2006; Koren and Rosenberg, 2006; Sertan-de Guzman *et al*., 2007; Muscholl-Silberhorn *et al*., 2008; Riesenfeld *et al*., 2008; Kennedy *et al*., 2009; Rypien *et al*., 2010; Santos *et al*., 2010; O’Halloran *et al*., 2011; Penesyan *et al*., 2011; Chiu *et al*., 2012; Flemer *et al*., 2012). Interestingly, all strains besides *P. denitrificans*, *P. japonicus*, a *Pseudovibrio*-related isolate from coastal, oligotrophic seawater (Agogué *et al*., 2005), and an epibiont of a red algae (Penesyan *et al*., 2011), were found to be associated with marine invertebrates like tunicates, corals, and mainly sponges. For instance, strains belonging to this genus have been described to be the dominating species of the culturable bacterial community in the sponges *Rhopaloeides odorabile*, *Suberites domuncula*, *Clathrina clathris*, and others (Webster and Hill, 2001; Muscholl-Silberhorn *et al*., 2008). Moreover, *Pseudovibrio*-related bacteria were the most abundant prokaryotes associated with larvae of the sponge *Mycale laxissima*, indicating vertical transmission of these bacteria in their hosts (Enticknap *et al*., 2006). The consistent co-occurrence of *Pseudovibrio*-related bacteria associated with sponges suggests that this genus contains symbionts of those metazoa (Webster and Hill, 2001; Enticknap *et al*., 2006). Whether the nature of this symbiosis is mutualistic/commensalistic or whether *Pseudovibrio* spp. rather represent pathogens/parasites is uncertain. However, the fact that *Pseudovibrio*-related bacteria have been isolated only from healthy sponges indicates that the bacteria do not harm the host and might be even required for its health (Webster and Hill, 2001; Webster *et al*., 2002).

Another feature shared by many cultured *Pseudovibrio* strains is the production of secondary metabolites. For instance, heptylprodigiosin, an antimicrobial compound, was isolated from a pure culture of *P. denitrificans* Z143-1 (Sertan-de Guzman *et al*., 2007). Furthermore, biosynthesis of the antimicrobially active tropodithietic acid (TDA) could be shown in an algal *Pseudovibrio*-related epiphyte (Penesyan *et al*., 2011) and a novel polypeptide with antimicrobial abilities (pseudovibrocin) was isolated from a coral-derived *Pseudovibrio*-related species (Vizcaino, 2011). Bioactivity of bacteria belonging to the *Pseudovibrio* genus could also be shown in other studies (Hentschel *et al*., 2001; Thiel and Imhoff, 2003; Muscholl-Silberhorn *et al*., 2008; Kennedy *et al*., 2009; Rypien *et al*., 2010; Santos *et al*., 2010; O’Halloran *et al*., 2011; Flemer *et al*., 2012), but the compounds were not further analysed.

Despite the fact that members of the genus *Pseudovibrio* seem to be ubiquitous, important associates of marine invertebrates and are also found free-living, very little is known about their overall physiology as well as the interactions with their hosts. In this study, we analysed the genomes of two *Pseudovibrio* strains. *Pseudovibrio* sp. FO-BEG1 has been isolated from an enrichment culture of *Beggiatoa* sp. 35Flor, a filamentous, sulfide-oxidizing bacterium (Brock and Schulz-Vogt, 2011; Schwedt, 2011). Initially, this *Beggiatoa* strain was sampled from a black band diseased scleractinian coral off the coast of Florida. This indicates that the strain *Pseudovibrio* sp. FO-BEG1 could have been associated with the coral at the time of sampling-either in a commensalistic/mutualistic or pathogenic relationship. The strain is now available as an axenic culture in our lab. Intriguingly, strain FO-BEG1 is also maintained in a co-culture with *Beggiatoa* sp. 35Flor, which is unable to grow on its own and therefore seems to be dependent on strain FO-BEG1. The second strain, *Pseudovibrio* sp. JE062, has been isolated off the coast of Florida from the sponge *Mycale laxissima* in the year 2006 and was described as a sponge symbiont by Enticknap and colleagues (2006). The axenic culture of this strain was kindly made available by Prof. R. Hill to be incorporated in this study. The draft genome of the latter strain was used as available in the public databases. The analysis of these genomes gives us an insight into the physiological and symbiotic potential of both *Pseudovibrio* strains revealing fascinating microorganisms that seem to be adapted to free-living as well as symbiotic lifestyles.

## Results and discussion

### Phylogenetic affiliation of *Pseudovibrio* strains FO-BEG1 and JE062

**Figure 1:**
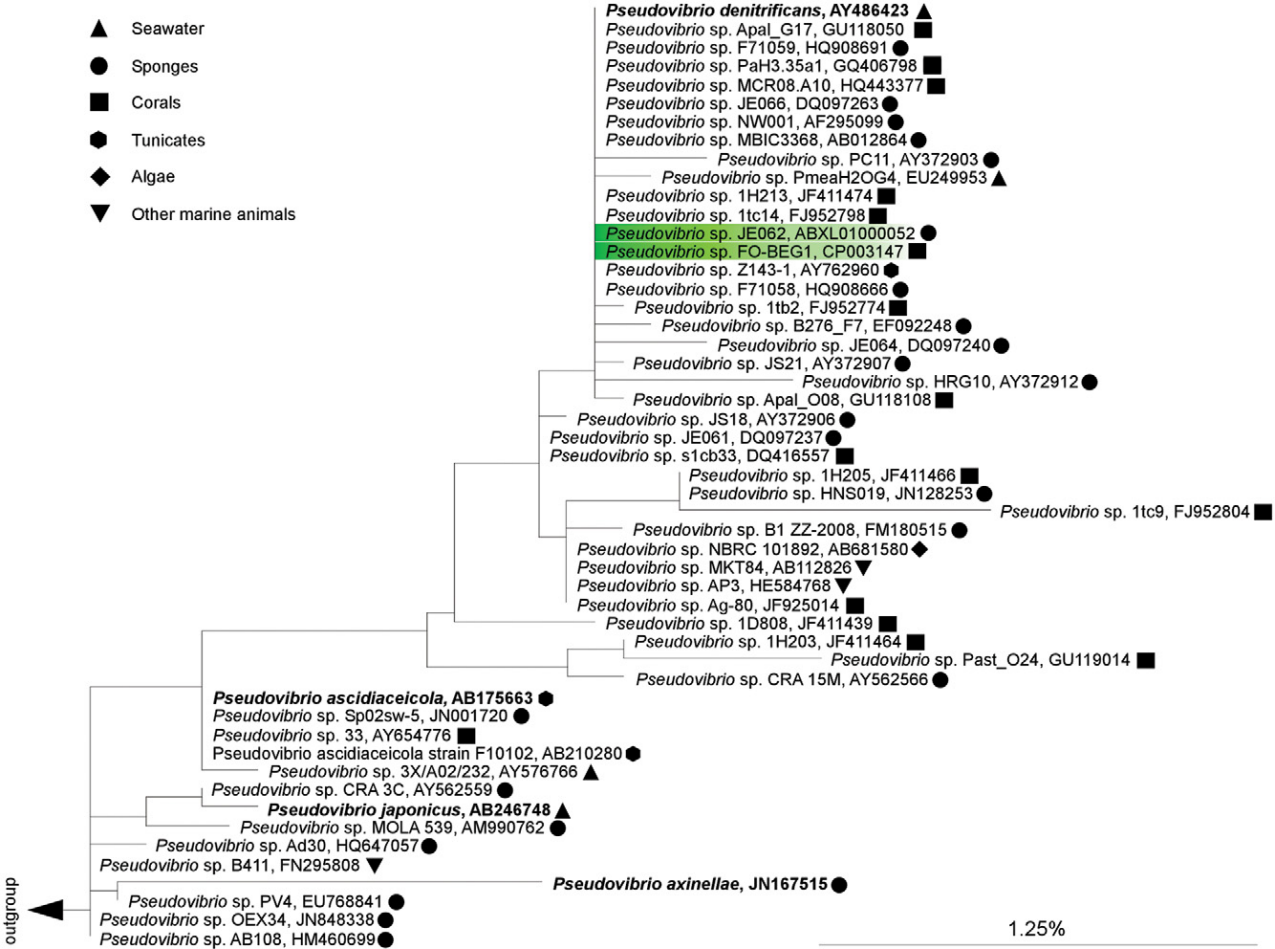
Consensus tree of nearly full-length 16S rDNA sequences of the *Pseudovibrio* genus, calculated with nucleotides at positions between 101 and 1405 according to *E. coli* numbering. Sequences belonging to the *Chloroflexaceae* have been used as out-group to root the tree. The type strains of the *Pseudovibrio* genus are shown in bold and the sequences investigated in this study are highlighted in green colour. The symbols behind the sequences indicate the isolation source. The isolation source of ‘Other marine animals’ refers to the sequences of *Pseudovibrio* sp. s1cb33 and *Pseudovibrio* sp. B411, which have been isolated from the abalone *Haliotis diversicolor* and the bryozoan *Cellepora pumicosa*, respectively, according to the NCBI database. For the sequence *Pseudovibrio* sp. MKT84 only the information ‘marine animals’ as isolation source was available. The bar represents 1.25% sequence divergence.

The investigated strains FO-BEG1 and JE062 belong to the *Pseudovibrio* genus, which, as shown in the description of the type strain *P. denitrificans* (Shieh *et al*., 2004), forms a separate cluster within the *Alphaproteobacteria* (Fig. S1). The two strains, which are completely identical on the 16S rDNA level, are monophyletic (99.9%) with the *P. denitrificans* type strain (Fig. 1). As mentioned previously, *Pseudovibrio*-related bacteria have been detected in coastal seawater and associated with sponges, corals, tunicates, and algae. Interestingly, the phylogenetic analysis of the 16S rDNA sequences affiliated with the *Pseudovibrio* genus did not show any clustering of the sequences according to their isolation source (Fig. 1). This implies the presence of adaptation mechanisms in *Pseudovibrio*-related bacteria to free-living and host-associated lifestyles throughout the *Pseudovibrio* genus.

### General genome characteristics of *Pseudovibrio* strains FO-BEG1 and JE062

**Figure 2:**
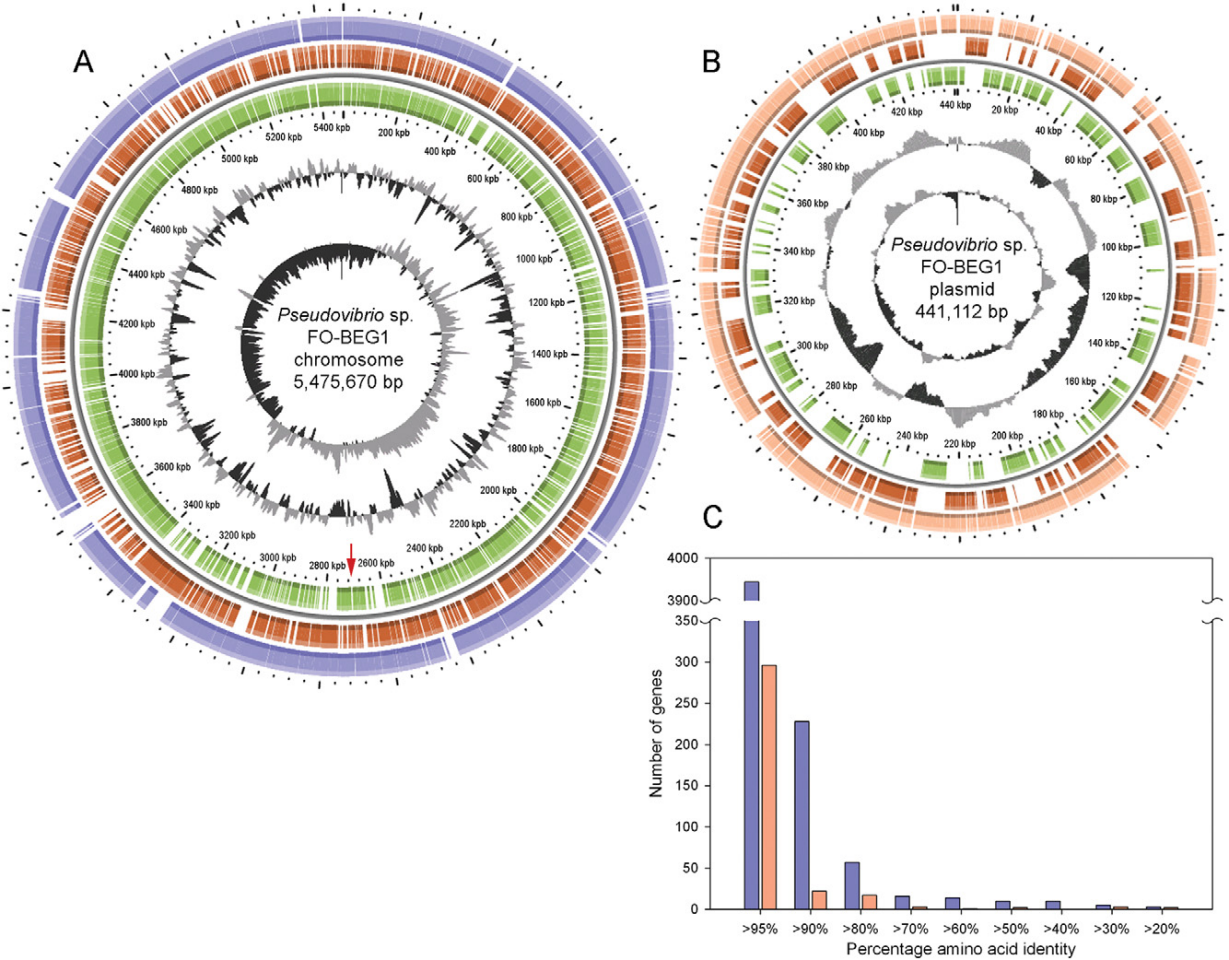
Comparative circular map of the *Pseudovibrio* sp. FO-BEG1 chromosome (A) and the plasmid (B). Most outer lane represents the reciprocal best match (RBM)-shared gene content between FO-BEG1 and JE062. Lane two and three represent all predicted ORFs on the lagging (red) and leading (green) strands. The two inner lanes display the GC-plot and the GC-skew. The red arrow indicates a sequence stretch of 3.4–18.4 kb that could not be closed during sequencing. The bar chart (C) expresses the amino acid percentage identity of each RBM shared gene-content between FO-BEG1 and JE062. The blue bar is representing the FO-BEG1 chromosome and orange the corresponding plasmid.

The genome size of strain FO-BEG1 was 5.9 Mbp, including a large plasmid of 0.4 Mbp (Fig. 2). The circular chromosome of 5.5 Mbp contained a large stretch of repeats at position 2 707 040. This area was present completely on one fosmid and had a predicted size between 3.4 and 18.4 kb, based on the minimal (30 kb) and maximal (45 kb) insert size of fosmids in vector pCC1. However, it could not be bridged with primer walking and a direct sequencing approach, indicating strong secondary structures, and has been masked with the ambiguous nucleotide code ‘N’. The G + C content of the genome was 52.5 mol%, which is consistent with the known values of described *Pseudovibrio* isolates (Shieh *et al*., 2004; Fukunaga *et al*., 2006; Hosoya and Yokota, 2007). Altogether, we found 5478 open reading frames (ORFs), 398 of which were located on the plasmid. This number of ORFs corresponded to about 87% of encoding DNA. Six complete rRNA operons and 69 tRNA encoding regions were annotated, indicating the capability of a quick response to changing environmental conditions and fast growth when nutrients are available. The genome of strain JE062 has not been closed, but there were 19 contigs available with an overall size of 5.7 Mbp, 5225 ORFs and 52.4 mol% G + C content, which was almost identical to that of the genome of strain FO-BEG1 (Fig. 2A and B). The draft genome contained 72 tRNA genes and seven complete rRNA operons. Unfortunately, the repeat-rich area that could not be sequenced in the genome of strain FO-BEG1 showed an ambiguous sequence in strain JE062 as well, and could therefore not be used to close the gap in FO-BEG1. Even though the genome of JE062 has not been completely closed, we assumed that it also contained a plasmid with a similar content, since most of the genes identified on the plasmid of FO-BEG1 were allocated in the genome of JE062 (Fig 2B). Tables 1 and S1 show an overview of the genome characteristics of *Pseudovibrio* sp. FO-BEG1 and JE062 and the assignment of the genes of both strains to cluster of orthologous group (COG) categories respectively. The shared gene content between FO-BEG1 and the draft genome of JE062 comprised 84.4% (4287 ORFs, Fig. 2C). An average nucleotide identity (ANI) analysis conducted between strains FO-BEG1 and JE062 revealed a 94.5% ANIb (87% genome alignment) and 95.4% ANIm (86% genome alignment) value. The values were in the range of the proposed species definition boundary (Richter and Rosselló-Móra, 2009), indicating a species level degree of similarity.

**Table:** General genome features of *Pseudovibrio* sp. FO-BEG1 and JE062

Characteristics	FO-BEG1	JE062
Base pairs	5 916 782	5 726 521
Contigs	2	19
G + C content (%)	52.5	52.4
No. of protein-coding genes	5 478	5 225
Percent coding	85.5	85
No. of rRNA operons	6	7
No. of tRNA genes	69	72

### Genomic and physiologic analysis of strains FO-BEG1 and JE062 reveal novel metabolic abilities within the *Pseudovibrio* genus

In both genomes we found a number of genes that indicate high metabolic versatility of *Pseudovibrio* sp. FO-BEG1 and JE062, most of which could be verified in physiological experiments with both strains. Degradation of carbohydrates was most likely performed via the Entner–Doudoroff pathway, enzymes of which were present in both genomes, and not by the Embden–Meyerhof–Parnas pathway, since the gene for the phosphofructokinase (PFK), a key enzyme of the glycolysis, was absent. This is a regularly encountered phenomenon within marine *Alphaproteobacteria* (Fürch *et al*., 2009; Tang *et al*., 2009; Williams *et al*., 2009). Besides the PFK, all other enzymes involved in glycolysis could be identified in both genomes, including fructose-1,6-bisphosphatase II, the key enzyme for gluconeogenesis, indicating that the Embden–Meyerhof–Parnas pathway could be used for anabolic purposes (Table S2). All genes encoding the enzymes of the citric acid cycle and pentose phosphate pathway were present.

Additionally, both strains had the genetic potential to aerobically degrade aromatic compounds via the β-ketoadipate pathway, which we demonstrated by growing *Pseudovibrio* sp. FO-BEG1 and JE062 with 4-hydroxybenzoate as the only carbon and energy source under aerobic conditions (Figs S2A and S5A). Benzoate, however, was not degraded, indicating that either the uptake of benzoate was absent or the hydroxylation of the aromatic ring structure in the *para* position to form the intermediate protocatechuate could not be catalysed by *Pseudovibrio* sp. FO-BEG1 and JE062. Under anoxic conditions without nitrate, both strains metabolized glucose in a mixed acid type fermentation, as suggested by the respective genes present in both strains (Table S2). Glucose consumption was accompanied by the acidification of the medium and the formation of mainly formate, lactate, acetate, and ethanol. Ethanol production during fermentation has not yet been described for any *Pseudovibrio* strain. Additionally, pyruvate, propionate, and succinate were formed, but to a lesser extent (Figs S3A and S6A). Trace amounts of fumarate were detected, but could not be quantified. As expected, we found the complete set of genes essential for denitrification, including a membrane-bound (*nar*) and a periplasmic nitrate reductase (*nap*). This was consistent with the complete denitrification to N_2_ that was observed in laboratory experiments with strains FO-BEG1 and JE062 (Table S2, Figs S3C and S6C). No genes for assimilatory nitrate reduction could be identified in either genome. For the type strain *P. denitrificans*, simultaneous denitrification and fermentation was described by Shieh and colleagues (2004) and was confirmed in our experiments for the strains FO-BEG1 and JE062 with acetate, formate, lactate, and ethanol as the main fermentation products (Figs S3B and S6B).

A set of *sox* genes suggested that both bacteria can use reduced inorganic sulfur compounds as a source of energy to complement heterotrophy. We could show experimentally that the addition of thiosulfate to the medium enhanced the aerobic growth of both *Pseudovibrio* cultures while sulfate accumulated in the course of the incubation (Fig. S2B, C and Fig. S5B, C). No tetrathionate could be measured as an intermediate in strain FO-BEG1 (results not shown). Therefore, we propose that both strains oxidize thiosulfate directly to sulfate, as it is typical for the Sox pathway in *Alphaproteobacteria* (for review, see Ghosh and Dam, 2009). We detected the complete Dimethylsulfoniopropionate (DMSP) cleavage pathway in the genomes of both *Pseudovibrio* strains (Table S2). Furthermore, we could also identify homologues of genes of the demethylation pathway in both genomes (Table S2), however, with a low sequence identity to the respective genes of *Ruegeria pomeroyi* DSS-3 (Reisch *et al*., 2011). DMSP is produced by phytoplankton as an osmoprotectant. Since it is abundant in the oceans (Yoch, 2002), it could be an important carbon and energy source for *Pseudovibrio*-related bacteria, especially strains, which are closely associated with algae (Penesyan *et al*., 2011). We confirmed that strains FO-BEG1 and JE062 were capable of growing with DMSP as the sole carbon and electron source (Fig. S4C). However, utilization of DMSP as the sole sulfur source could not be confirmed for the analysed strains over the monitored time span (Fig. S4C). This indicated that methanethiol (MeSH), which is required for the incorporation of the sulfur into macromolecules and is a product of the hydration reaction of DmdD in the last step of the demethylation pathway (Kiene *et al*., 1999; Reisch *et al*., 2011), was not produced by the analysed *Pseudovibrio* strains. This suggested that either the demethylation pathway was not active or the DmdD was not functional in strains FO-BEG1 and JE062.

We identified genes encoding a small (*cutS*), medium (*cutM*) and large (*cutL*) subunit of the aerobic form II carbon monoxide dehydrogenase (CODH) with the accessory gene *coxG* present in the respective operon of both genomes (Table S2), indicating the capability of CO oxidation. However, uptake of CO could not be demonstrated under applied conditions with strains FO-BEG1 and JE062 (results not shown). Interestingly, this result confirmed the hypothesis from a recent publication testing CO oxidation in bacteria containing type II CODH genes (Cunliffe, 2011), in which none of the isolates containing only the type II variant was capable of CO oxidation. Since only bacteria containing the form I CODH did effectively oxidize CO, it was questioned whether form II CODH is involved in the process of carbon monoxide oxidation, or if it has another primary function not known until now, as suggested by King and Weber (2007).

In both *Pseudovibrio* strains, we found genes for phosphonate import and degradation via the C-P lyase pathway, which allows bacteria to cleave the extremely stable C-P bonds of phosphonates (Table S2). Thereby, they can metabolize a less accessible phosphorous pool in times of phosphate limitation. We demonstrated growth of *Pseudovibrio* sp. FO-BEG1 and JE062 with phosphonoacetate as the only source of phosphorous (Figs S4A and S7A). Additionally, we demonstrated the adaptation of *Pseudovibrio* strain FO-BEG1 to oligotrophic conditions by culturing it with as little as 5 μmol C l^−1^ (0.06 mg C l^−1^) dissolved organic carbon in the medium (Schwedt, 2011). Furthermore, we were able to grow strain JE062 in the oligotrophic medium prepared according to Schwedt (2011) up to cell numbers of 6 × 10^4^ cells ml^−1^ (Fig. S7C), which showed that both *Pseudovibrio* strains were capable of growth under extreme nutrient depletion. Taken together, all experimental analyses performed led to comparable results for the two analysed strains.

The high metabolic versatility of *Pseudovibrio* sp. FO-BEG1 and JE062 was also reflected in the analysis of encoded primary transporters. In the genome of strain FO-BEG1 we could identify 31 tripartite ATP-independent periplasmic (TRAP) type transporters (Table S3) that are required for import of dicarboxylic acids like malate, succinate and fumarate, one of the highest numbers of TRAP type transporters reported in genomes of marine prokaryotes so far (compare Wagner-Döbler *et al*., 2010). In strain JE062 we identified 27 TRAP transporters. Citric acid cycle intermediates seem therefore to be an important source of carbon and energy for the investigated *Pseudovibrio* strains. In addition, we reconstructed over 80 ATP binding cassette (ABC) transporter systems with predicted substrate specificity from the genomic data of the strain FO-BEG1, and over 70 ABC transporter systems for JE062 (Tables 2 and S4). Sugars, oligopeptides and amino acids were the main putative substrates of the identified ABC systems, based on the *in silico* predictions. A large number of transporters for oligopeptides and amino acids in combination with over 85 genes encoding peptidases, proteases or their subunits (over 75 genes in strain JE062, see Table S5) could help the analysed *Pseudovibrio* strains to hydrolyse complex organic compounds into oligopeptides and amino acids, which could serve as nutrition for both the prokaryote and the host, as has been suggested by Siegl and colleagues (2011). We further identified eight iron transporters in each of the genomes, including three transporters for hemin and three ABC transport systems for siderophores (Table 2). As suggested by Egli (2010), a possibility for bacteria to survive and multiply in oligotrophic environments is to import and utilize a variety of different substrates simultaneously. The metabolic versatility and the high number of transporters encoded in the genomes of both strains provide therefore a potential explanation for the capability of both strains to grow with low amounts of organic material.

**Table:** Identified ABC and TRAP transporters in the genomes of both *Pseudovibrio* strains and their putative functions

	Transporters identified	
Transporter type and proposed function	FO-BEG1	JE062
TRAP transporter for dicarboxylates	31	27
ABC Transporter for:		
Sugars	22	19
Oligopeptides	15	13
Amino acids	12	10
Putrescine/spermidine	5	5
Glycerol 3-phosphate	4	4
Glycine betaine/L-proline	3	3
Glycine betaine/carnitine/choline	1	0
Taurine	1	1
Thiamine	1	1
Urea	1	1
Nopaline	1	1
Hemin	3	3
Enterobactin	1	1
Ferrichrome	1	1
Anguibactin	1	1
Iron	2	2
Manganese/zinc	2	2
Molybdenum	1	1
Cobalt	1	1
Sulfonate	2	2
Phosphate	1	1
Phosphonate	1	1

### *Pseudovibrio* sp. FO-BEG1 and JE062 possess possible biosynthetic pathways for different vitamins

Growth of pro- and eukaryotes highly depends on their requirements for cofactors that the organism can or cannot synthesize on its own. Vitamins are important for many different enzymatic processes and the synthesis of some vitamins is mainly accomplished by bacteria, making the prokaryotes a necessary part of the eukaryotic diet or an important partner in symbiotic relationships. The genomes of *Pseudovibrio* sp. FO-BEG1 and JE062 contain genes encoding key enzymes of the biosynthesis pathways of biotin (H), thiamin (B_1_), pyridoxin (B_6_), cobalamin (B_12_), riboflavin (B_2_), folic acid (B_9_) and lipoic acid (Table S6). Some genes are missing in the genome of strain JE062, most likely due to its draft status, since the vast majority of the required genes can be detected in this strain (Table S6). Independence from an external vitamin supply was confirmed during aerobic growth in the defined, vitamin-free Carbohydrate/mineral (CM) medium, which implies *de novo* synthesis of all required growth factors by the strains FO-BEG1 and JE062 under tested conditions (Figs S4B and S7B). The analysed *Pseudovibrio* strains would therefore be beneficial companions for other marine prokaryotes or eukaryotes, since the dependency on an external supply of those vitamins would be relieved.

### The analysed strains are capable of the production of bioactive compounds and strain FO-BEG1 possesses a potential colibactin synthesis machinery

Symbiotic relationships between bacteria and marine invertebrates, especially sponges, have been of special interest, since many bioactive compounds from sponges are suspected or have been shown to be of bacterial origin (Piel *et al*., 2004; Taylor *et al*., 2007; Fisch *et al*., 2009). Secondary metabolites were investigated in several *Pseudovibrio*-related strains, resulting in the identification and isolation of, e.g. heptylprodigiosin (Sertan-de Guzman *et al*., 2007) and pseudovibrocin (Vizcaino, 2011). Furthermore, Penesyan and colleagues (2011) isolated an epiphytic *Pseudovibrio* strain closely related to *P. ascidiaceicola*, producing the antimicrobial compound TDA. Interestingly, genes proposed to be involved in the biosynthesis of TDA have already been identified in the genome of the *Pseudovibrio* strain JE062 by Geng and Belas (2010) and during our investigations we detected homologues of these genes also in *Pseudovibrio* strain FO-BEG1 (Table S7). In fact, we detected TDA in strains FO-BEG1 and JE062 during growth in marine broth medium under static conditions (2.0 mg l^−1^ and 1.23 mg l^−1^ respectively). TDA production under these conditions has previously been shown for bacteria of the *Roseobacter* clade (Geng *et al*., 2008; Geng and Belas, 2010). Many bioactive compounds can be produced by bacteria using polyketide synthases (PKS) (Staunton and Weissman, 2001) and nonribosomal peptide synthetases (NRPS) (Finking and Marahiel, 2004). O’Halloran and colleagues (2011) identified PKS genes in *Pseudovibrio*-related bacteria, including one PKS gene in the strain JE062. We could likewise detect this 7.4 kb large type I PKS gene homologue on the plasmid of strain FO-BEG1 (Table S7).

**Figure 3:**
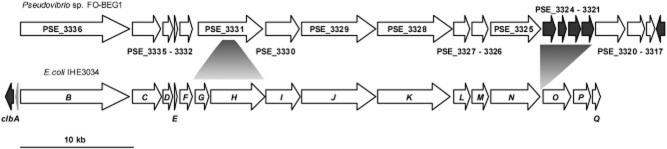
The nonribosomal peptide synthetase-polyketide synthase (NRPS-PKS) system in *Pseudovibrio* sp. FO-BEG1 and *Escherichia coli* strain IHE3034. White arrows represent the genes present in *Enterobacteriaceae* and strain FO-BEG1; black arrows represent the ORFs present only in either *Enterobacteriaceae* or FO-BEG1 but presumably involved in the production of colibactin; the gray arrow shows a gene presumably not involved in the synthesis of colibactin. The symbol at ORF PSE_3331 represents a gene fusion of *E. coli* genes *clbG* and *clbH* in FO-BEG1; the symbol at PSE_3324-3321 represents a gene insertion or deletion in strain FO-BEG1 or *E. coli* IHE3034 respectively.

In the chromosome of *Pseudovibrio* sp. FO-BEG1 we identified an intriguing genomic island of more than 50 kb containing a gene cluster of 20 genes predicted to be involved in the production of a bioactive compound (Table S7). The cluster exhibited high sequence similarity to an architecturally almost identical hybrid nonribosomal peptide synthetase-polyketide synthase (NRPS-PKS) system previously reported from many pathogenic and commensalistic *Escherichia coli* strains (Fig. 3) (Nougayrède *et al*., 2006). The *E. coli* metabolite, termed colibactin, remains structurally uncharacterized, as its synthesis is dependent on the contact of *E. coli* to mammalian cells, which hindered the isolation of this compound. However, transposon mutagenesis of the gene cluster suggested that colibactin is a pathogenicity determinant that induces DNA double strand breaks in eukaryotic host cells, leading to cell cycle arrest and eventually resulting in cell death. The only significant difference between the gene cluster in *Pseudovibrio* sp. FO-BEG1 and *E. coli* was an additional set of genes in FO-BEG1, encoding putative transporters and the presence of a different phosphopantetheinyl transferase gene variant, likely involved in generating *holo*-proteins from *apo* forms of PKSs and NRPSs (Lambalot *et al*., 1996). In addition, two *E. coli* genes were fused in the *Pseudovibrio* sp. FO-BEG1 cluster. Despite these differences, the architecture strongly suggests that the product of the FO-BEG1 cluster is colibactin, providing new opportunities to unveil the identity of this elusive and biomedically relevant compound. Interestingly, we found this NRPS-PKS fragment only in *Pseudovibrio* sp. FO-BEG1 but not in the genome of strain JE062. Since the flanking regions of the inserted fragment were highly conserved in synteny in strain JE062 (data not shown), it is likely that strain FO-BEG1 acquired the genetic island via horizontal gene transfer.

### Mechanisms for DNA exchange and horizontal gene transfer in strains FO-BEG1 and JE062

A set of genes coding for a complete gene transfer agent (GTA; a unit best described as a virus) was identified in the genome of strain FO-BEG1. In strain JE062, several of these genes were missing (see Table S7). GTAs harbour small parts of the host DNA and are capable of injecting it into appropriate cells, without having negative effects on the host cell (for reviews see Lang and Beatty, 2001; 2007). Using GTAs, the analysed strains could have taken up and dispersed DNA carried in virus-like particles, thereby gathering genes and establishing a versatile physiology for both, symbiotic and free-living lifestyles. Additionally, we found 14 integrase and 21 transposase elements in the genome of *Pseudovibrio* sp. FO-BEG1 (Table S7). Nine of the transposases were located adjacent to the hybrid NRPS-PKS gene cluster, supporting the proposed acquisition of this genomic island via horizontal gene transfer. In strain JE062, we could detect 11 integrases and 1 transposase.

### Strains FO-BEG1 and JE062 seem to be able to respond to but not synthesize quorum sensing molecules

Proteins containing the carboxy-terminal DNA binding LuxR domain were identified 13 and 12 times in strains FO-BEG1 and JE062 respectively. LuxR proteins are transcriptional regulators of N-acyl homoserine lactone (AHL) based quorum sensing systems. In addition to the identified carboxy-terminal DNA binding helix–turn–helix (HTH) motif, a functional LuxR transcriptional regulator requires the presence of an amino-terminal autoinducer binding domain (Whitehead *et al*., 2001; Fuqua and Greenberg, 2002). Binding of AHLs to the autoinducer domain activates the DNA binding domain of LuxR and thus the transcriptional activation of selected genes. Two of the identified genes encoded both, an autoinducer- and a DNA-binding domain (Table S8) in each strain, indicating that both strains are able to detect and respond to AHL quorum sensing molecules. Four further genes with a carboxy-terminal DNA binding domain contained an amino-terminal receiver domain of a response regulator (Table S8), which are usually activated via the phosphorylation by the respective sensory histidine kinase. Therefore, these proteins appear to be regulators of two component regulatory systems. The amino-terminal parts of the remaining seven (six in strain JE062; Table S8) proteins with a LuxR DNA binding domain were not homologous to domains with a known function. Similar LuxR-like proteins have already been identified in other bacteria and it was suggested that they are operating in an autoinducer-independent way or react to quorum sensing signals other then AHLs (Patankar and González, 2009; Subramoni and Venturi, 2009). Even more intriguingly, we could not find any *luxI* homologues, which code for the AHL synthase. This observation led us to the hypothesis that both *Pseudovibrio* strains do not communicate via AHLs within their own species, but seem to use the LuxR as receptors to react to quorum sensing molecules produced by other species in order to initiate a respective answer. Such a scenario has been described before by Case and colleagues (2008) and was called ‘eavesdropping’. The response reaction could include the production of bioactive compounds to repel competing prokaryotes or to protect the host from pathogens or parasites. Alternatively, such LuxR-family ‘solos’ could participate in interkingdom signalling, as suggested by Subramoni and Venturi (2009), thereby facilitating prokaryote–host interactions of the investigated *Pseudovibrio* strains with, e.g. marine invertebrates.

### Growth of *Pseudovibrio* sp. FO-BEG1 with *Beggiatoa* sp. 35Flor

*Pseudovibrio* sp. FO-BEG1 was the sole accompanying organism of the *Beggiatoa* strain 35Flor, which was growing in a chemolithoautotrophic oxygen-sulfide-gradient medium (Brock and Schulz-Vogt, 2011; Schwedt, 2011). All attempts to grow *Beggiatoa* sp. 35Flor in the absence of *Pseudovibrio* sp. FO-BEG1 failed, and so far we could not identify the factors required by the *Beggiatoa* strain for autonomous growth. It is known, however, that *Beggiatoa* spp. do not possess catalases (Larkin and Strohl, 1983) and therefore are susceptible to hydrogen peroxide generated during respiration. As the genomes of both *Pseudovibrio* strains contained reactive oxygen species protection systems consisting of genes encoding over 20 superoxide dismutases, catalases and peroxidases (Table S5), it seems plausible to assume that *Beggiatoa* sp. 35Flor benefits from the detoxification of reactive oxygen species by their accompanying organism. However, the addition of catalase to the *Beggiatoa* medium alone was not sufficient to release the dependency of the *Beggiatoa* strain on *Pseudovibrio* sp. FO-BEG1 (A. Fink, pers. comm.). Hence, further so far unknown factors seem to be involved in this symbiotic relationship.

### Two types of secretion systems could be involved in symbiont–host interactions of the investigated strains

In the genomes of FO-BEG1 and JE062 we could identify two loci that encode type VI secretion systems (T6SSs) as well as one type III secretion system (T3SS), including effector molecules. These findings indicated the capability of specific interactions with eukaryotes and the possibility of influencing their cell machinery.

The T6SS has been described as a major secretion system in the context of pathogenicity as a virulence factor in pathogenic bacteria (Mougous *et al*., 2006; Pukatzki *et al*., 2006) and a core of 13 highly conserved and essential subunits has been identified for this secretion system (Boyer *et al*., 2009). In each of the *Pseudovibrio* genomes, we found two gene clusters of 12 (cluster I) and 20 (cluster II) genes that encode T6SSs. Cluster II contained the complete set of core subunits and therefore we assume that cluster II could, if expressed, produce a complete and functional T6SS. In cluster I, two core genes were missing in the operon, *hcpI* and *vgrG*, which are main components of the injection apparatus with possible effector functions (Pukatzki *et al*., 2009; Bönemann *et al*., 2010). However, homologues of *hcpI* and *vgrG* could be identified in additional copies at other locations in the genomes of FO-BEG1 and JE062 (Table S9), which is a phenomenon regularly found in genomes containing T6SSs (Pukatzki *et al*., 2009). Consequently, it can be assumed that the T6SSs of both strains are functional, since the genomes contained the main structural components of the T6SS. The possible role for T6SSs in bacteria has not been completely elucidated so far, but several functions have been attributed to it already. Mainly, T6SSs have been described as virulence factors of pathogenic bacteria delivering effector proteins into host cells (Filloux *et al*., 2008). However, further studies revealed the involvement of T6SSs in biofilm formation (Aschtgen *et al*., 2008), quorum sensing (Weber *et al*., 2009), interbacterial interactions (Hood *et al*., 2010) and antipathogenesis (Chow and Mazmanian, 2010; Jani and Cotter, 2010).

**Figure 4:**
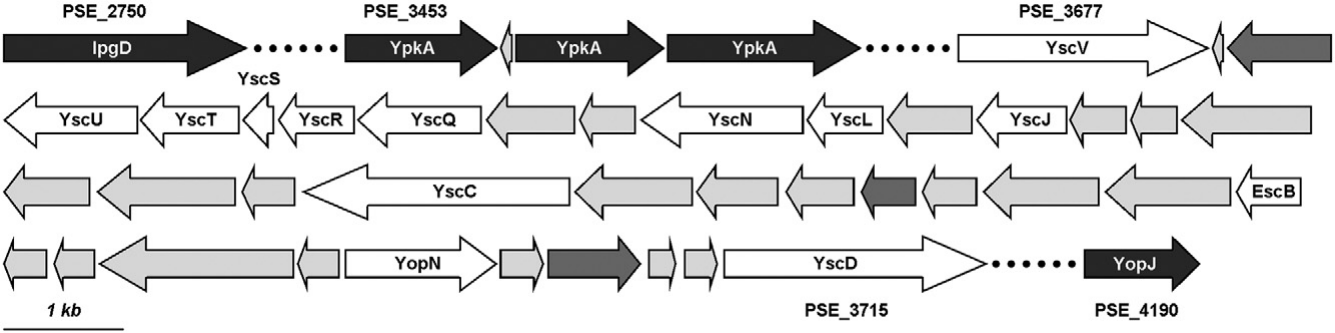
Operon encoding type III secretion system (T3SS) subunits and effector proteins in *Pseudovibrio* sp. FO-BEG1. White arrows show annotated homologues of T3SS subunits including the gene name within the arrows; black arrows represent annotated effector homologues; dark gray arrows show annotated genes coding for proteins presumably not involved in the T3SS; light gray arrows show hypothetical proteins with unknown function. The locus is indicated above and below some genes for orientation purposes.

In addition to the T6SSs, we identified a T3SS in the genomes of both *Pseudovibrio* strains (Fig. 4 and Table S9). It was located in a genomic region encompassing around 35 ORFs with various highly conserved proteins known from T3SSs (Cornelis and Van Gijsegem, 2000). Besides the secretion apparatus, we also identified genes encoding homologues of three types of effector molecules in the genome of strain FO-BEG1, and two effector molecule types in strain JE062. Those effectors might be directly involved in the establishment of a symbiosis between *Pseudovibrio* strains FO-BEG1 and JE062 and their hosts. YpkA, IpgD (found in both genomes) and YopJ (only in strain FO-BEG1) are homologues of effector molecules that affect the cytoskeleton or the innate immune response of the host respectively. YpkA is a serine/threonine kinase, which has negative effects on cytoskeletal dynamics due to its interaction with actin, thereby contributing to the resistance to phagocytosis (Cornelis, 2002). YpkA homologues were present in three copies in both genomes. In Porifera, specialized amoeboid cells, the archaeocytes, resembling macrophages, eliminate non-self material via phagocytosis (Müller and Müller, 2003). Also in corals phagocytotic activity has been described (Mydlarz *et al*., 2006). *Pseudovibrio*-related strains expressing and secreting the YpkA effector could interfere with this process, preventing phagocytic host cells from digesting *Pseudovibrio* cells. A similar effect could be induced by a homologue of IpgD found in both genomes, a virulence factor that is responsible for morphological changes of a host cell by increasing membrane detachment from the cytoskeleton (Niebuhr *et al*., 2000; 2002).

In strain FO-BEG1, we additionally identified a homologue of the YopJ effector, which has been shown to exhibit a serine/threonine acetyltransferase function. By acetylation of serine and threonine residues of mitogen-activated protein (MAP) kinases, it prevents phosphorylation of those molecules and therefore inhibits the innate immune response of the organism (Mukherjee *et al*., 2006). Analysis of the innate immune repertoire in Cnidaria on the genomic level indicates that those eukaryotes possess key components of the c-Jun N-terminal kinase (JNK)/MAP kinase pathway, proposed to be required for the activation of the innate immune response (Miller *et al*., 2007). Furthermore, it has been shown that also sponges possess a very efficient innate immune response system, using MAP kinases as the essential component of their response to the bacterial endotoxin lipopolysaccharide (LPS) (Böhm *et al*., 2001; Müller and Müller, 2003). Even though direct evidence of strain FO-BEG1 being associated with the coral during the sampling procedure is lacking, the genomic evidence indicates the potential to inhibit the JNK/MAP kinase pathway as a part of the innate immune response of the host coral, and, if necessary, also of a sponge.

The application of potential T3SS effector homologues by the *Pseudovibrio* strains FO-BEG1 and JE062 would allow these strains to avoid phagocytosis and to remain in the host for establishment of a symbiosis. This hypothesis is further supported by the fact that a homologue of YopJ (NopJ) was shown to be an effector in symbiotic rhizobia (Deakin and Broughton, 2009). Furthermore, Lackner and colleagues (2011) demonstrated that the T3SS is involved in the maintenance of a symbiosis between bacteria and fungi by enhancement of intracellular survival of the prokaryote within the host.

### Potential adhesion mechanisms and domains mediating prokaryote–eukaryote interactions in strains FO-BEG1 and JE062

In both genomes, we found homologues of genes coding for proteins responsible for adhesion to surfaces or other cells. These proteins, belonging to the group of amyloids, are extracellular proteinaceous components and are known in the *Enterobacteriaceae* as curli fibres. They are involved in adhesion to surfaces, cell aggregation, biofilm formation and mediate cell–cell adhesion and invasion of host cells (Barnhart and Chapman, 2006). The production of curli fibres in enteric bacteria is dependent on at least six proteins encoded by the operons *csgAB* and *csgDEFG* (*agf* in *Salmonella*) (Hammar *et al*., 1995), the latter of which is required for assembly, stability, and secretion of the amyloids (Hammar *et al*., 1995). *csgAB* encodes the structural subunits of the curli fibres, both genes containing characteristic repeat motifs (Hammar *et al*., 1996). A gene cluster in the genomes of *Pseudovibrio* sp. FO-BEG1 and JE062 resembled the curli formation operon in enteric bacteria (Table S8 and Fig. S8). Homologues of *csgF* and *csgG*, required for stabilization and secretion of the amyloids were found in direct proximity to three genes containing curlin associated repeats as typical structural components of the curli fibres. We hypothesize that the identified genomic region might code for amyloid structures comparable to curli fibres due to the existence of characteristic curlin repeat motifs and genes involved in the assembly and secretion of such structures. This mechanism might therefore allow *Pseudovibrio* sp. FO-BEG1 and JE062 to attach to host cells or to form biofilms and aggregates. Moreover, in both strains we could identify a complete *tad* (tight adherence) locus required for the assembly of adhesive Flp (fimbrial low-molecular-weight protein) pili (Table S8), mediating biofilm formation in different bacteria (Tomich *et al*., 2007). The identified cluster is also frequently found in bacteria belonging to the *Roseobacter* clade (Slightom and Buchan, 2009) and is homologous to the *cpa* genes of *Caulobacter crescentus* (Skerker and Shapiro, 2000), further supporting the proposed ability of both *Pseudovibrio* strains to form biofilms and colonize surfaces. Additionally, we identified 35 genes in strain FO-BEG1 and 32 in JE062 containing domains presumably mediating prokaryote–eukaryote interactions. This supports the proposed role of both *Pseudovibrio* strains as symbionts with possibilities to attach and interact with the host organism (Table S8).

### Conclusions

In this study, we analysed for the first time the genomes of two *Pseudovibrio* strains that originate from the coast off Florida. *Pseudovibrio* sp. FO-BEG1 was isolated from a coral and maintained over 10 years in co-culture with a chemolithoautotrophic *Beggiatoa* strain. *Pseudovibrio* sp. JE062 was isolated from a sponge in the same region (Enticknap *et al*., 2006). The genomes predict an extremely versatile physiology for both strains, which could partially be verified in experiments with the isolated strains. Here, we describe for the first time that bacteria of the genus *Pseudovibrio* oxidize thiosulfate under aerobic conditions, use aromatic compounds and DMSP as a carbon and electron source, and use phosphonates as a phosphorous source. The metabolic versatility is confirmed by the numerous transporter systems that are encoded in both genomes. Notably, both strains are able to grow with extremely low concentrations of dissolved organic compounds, which emphasizes their adaptation to life in the open ocean. Compared with other marine bacteria, like the closely related, prominent *Roseobacter* clade, which is known to be ubiquitous, multitudinous and physiologically versatile (Newton *et al*., 2010), the analysed *Pseudovibrio* strains seem to be capable of a similarly generalistic lifestyle, exploiting quite a number of sources for energy, nutrients and trace elements.

Aside from metabolic versatility, the genomic data of both strains also confirm close associations with marine invertebrates and indicate several potential mechanisms for establishing and maintaining a symbiosis. The most striking discovery is the presence of effector homologues secreted by type III secretion systems, which could affect marine invertebrates by interacting with their immune response system (YopJ, only detected in strain FO-BEG1) or the cytoskeleton (YpkA, IpgD, detected in both strains) and thereby have a severe impact on the cellular machinery of the host.

We could show that the strains JE062 and FO-BEG1 produce the potent antimicrobial substance TDA. Assuming that both strains have the potential to establish a symbiosis with marine invertebrates, the production and secretion of secondary metabolites with antimicrobial activities would protect the host organism from pathogens. This indicates that the proposed role of a *Pseudovibrio*-related strain to be required for the health of the host as previously proposed (Webster and Hill, 2001; Webster *et al*., 2002) could also be true for the representatives of this genus analysed in this study. Another fascinating discovery is the presence of the hybrid NRPS-PKS system in strain FO-BEG1, which has so far only been described for members of the *Enterobacteriaceae* (Putze *et al*., 2009). This NRPS-PKS cluster is assumed to produce the bioactive compound colibactin with yet unknown *in vivo* functions. It was, however, shown, that colibactin arrests eukaryotic cells in the G_2_ phase, eventually leading to cell death. Inhibition of cell division might be used by the colibactin-producing *Enterobacteriaceae* to prolong the attachment to intestinal epithelium cells in order to successfully colonize the intestine of their host (Nougayrède *et al*., 2006). The presence of a gene cluster coding for a cytopathic compound emphasizes the impact that strain FO-BEG1 might have on marine eukaryotes, especially in the context of symbiosis establishment between the strain FO-BEG1 and corals or sponges. It is ambiguous whether strain JE062 truly lacks the colibactin gene cluster as well as the putative YopJ effector protein as an adaptation to a different ecological niche, or if this genomic information is missing simply due to the fact that the genome of JE062 is not completely closed. Intriguingly, strain FO-BEG1 seems to be required for cultivation of *Beggiatoa* sp. 35Flor, highlighting its symbiotic role not only for marine invertebrates but also for prokaryotes. It is possible that the investigated *Pseudovibrio* strains have positive effects on certain bacteria under *in vivo* conditions, e.g. by supplying vitamins or detoxifying metabolic intermediates, including reactive oxygen species.

**Figure 5:**
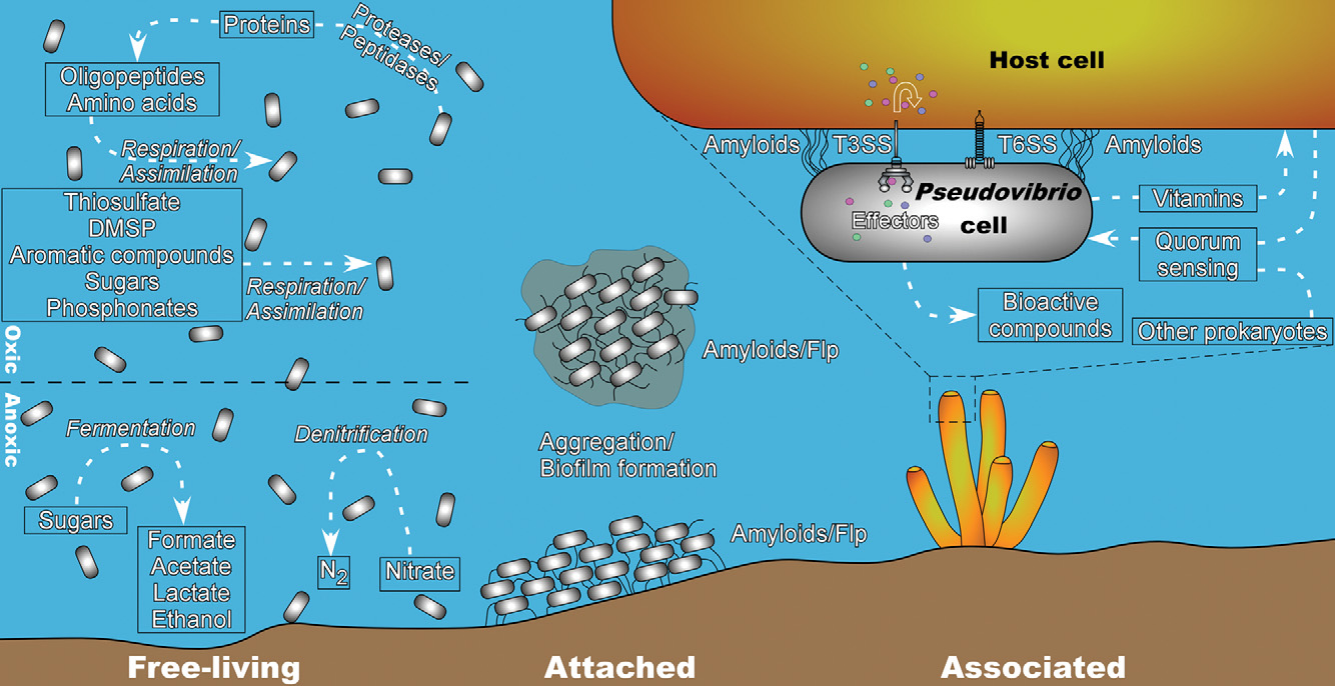
Schematic overview of the possible lifestyles and the physiologic capabilities derived from genetic information from the genomes of *Pseudovibrio* sp. FO-BEG1 and JE062. On the left hand side, physiologic abilities are depicted that could be used in free-living, oxic and anoxic conditions. On the right hand side, the attached or associated lifestyle is illustrated. The host organism for the associated lifestyle can be represented by a sponge, coral or tunicate. Biofilm formation and aggregation could be performed via, e.g. amyloid-like structures or the adhesive Flp (fimbrial low-molecular-weight protein) pili. Furthermore, the amyloid-like structures could be required for the attachment to host cells. The proposed secretion systems with the potential effector proteins could be involved in prokaryote–eukaryote interactions, influencing the cell machinery of the host. Additionally, both *Pseudovibrio* strains could supply the host with cofactors like vitamins or synthesize secondary metabolites like TDA as a defence mechanism against other prokaryotes or the host.

The frequent identification and isolation of *Pseudovibrio* strains in many studies over the last years suggests an important but rather unexplored role for this genus in marine habitats. According with the genomic and physiological data for *Pseudovibrio* sp. FO-BEG1 and JE062, we propose a free-living and attached or associated lifestyle model for the analysed strains (Fig. 5). As denitrifying heterotrophs, both *Pseudovibrio* strains have an obvious influence on the carbon and nitrogen cycles. With the presented information, their ecological impact can now be extended to both, organic and inorganic sulfur cycles and the phosphorus cycle due to their ability to metabolize thiosulfate, DMSP and phosphonates respectively. Additionally, we hypothesize that, due to the predictions based on the genomic data, similar to *E. coli* in humans, *Pseudovibrio* sp. FO-BEG1 and JE062 are commensalistic or even beneficial symbionts of marine invertebrates with a potential to become pathogenic.

## Experimental procedures

### Strains and accession numbers

In this study, the strains *Pseudovibrio* sp. FO-BEG1 and JE062 were analysed genetically and physiologically. Strain FO-BEG1 was sequenced as described hereafter. The genome shotgun project of strain FO-BEG1 has been deposited at DDBJ/EMBL/GenBank under the Accession Number CP003147 for the chromosome and CP003148 for the plasmid. Strain JE062 was sequenced in the year 2008 in the framework of the Marine Microbial Genome Sequencing Project (by the Gordon and Betty Moore Foundation) and the draft genome sequence was deposited at DDBJ/EMBL/GenBank with the Accession Number ABXL00000000. This publically available genome information was used in the following study. The physiological experiments were conducted with axenic cultures of both strains. For this purpose, strain JE062 was kindly provided to us by Prof. Russell Hill.

### Calculation of the phylogenetic tree

The phylogenetic analysis of the 16S rDNA sequences of the two investigated strains was inferred using the ARB software package (Ludwig *et al*., 2004) based on the release 111 of the SILVA SSU Ref database (Pruesse *et al*., 2007). Based on the phylogenetic analysis performed by Taylor and colleagues (2007), we manually selected bacterial 16S rDNA sequences belonging to the class *Alphaproteobacteria*, including all the available sequences affiliated with the *Pseudovibrio* genus. The number of sequences present in the SILVA database that was used for this study, however, was higher than the one initially used by Taylor and colleagues (2007). Therefore, we also added sequences belonging to the *Alphaproteobacteria*, which in the SILVA database were designated to be retrieved from the following sponge genera: *Halicondria*, *Pseudoceratina*, *Mycale*, *Phyllospongia*, *Microciona*, *Dysidea*, *Agelas*, *Cymbastela*, *Antho*, *Axinella*, *Spongilla*, *Plakortis*, and *Aplysina*. The sequences of the strains used for this study were already present in the SILVA database; therefore, the internal alignment was not further verified. In order to remove the redundant information and simplify the tree visualization we removed all sequences which shared 100% identity among each other and were obtained during the same study. A termini filter considering only nucleotide positions between 101 and 1405 (according to *E. coli* numbering) was calculated and used for all the phylogenetic analyses performed. Since the number of sequences present in the database selected was still very high, we also removed all sequences shorter than the above mentioned termini filter in order to simplify the obtained tree. Additionally, we included 16S rDNA sequences of the major clusters of the *Roseobacter* lineages based on the phylogenetic analysis performed by Buchan and colleagues (2005), as bacteria of this lineage represent important and abundant marine *Alphaproteobacteria* with a versatile metabolism and the potential to interact with marine eukaryotes. The accession numbers of the *Roseobacter* sequences can be found in Table S10. The obtained database was used for the reconstruction of the phylogenetic tree using the maximum likelihood (RAxML) method. The tree was obtained using the GTRGAMMA model and the rapid bootstrap analysis algorithm with 1000 repetitions. Sequences belonging to the *Chloroflexaceae* were included in the analysis as out-group. For the *Pseudovibrio* cluster, two additional trees were calculated using maximum likelihood (RAxML) with the GTRGAMMA model and the maximum parsimony method. The branching pattern of the two trees was manually compared and a consensus tree was constructed according to Peplies and colleagues (2008). Multiforcation were introduced at nodes where the exact tree topology could not be clearly resolved.

### Growth conditions for strains FO-BEG1 and JE062

For aerobic growth, CM medium modified after Shieh and colleagues (2004) was used. After autoclaving, the medium was supplemented with K_2_HPO_4_ (1.15 mmol l^−1^), glucose (10 mmol l^−1^ unless stated otherwise), 1 ml l^−1^ tungsten/selenium solution (Brysch *et al*., 1987), 1 ml l^−1^ trace elements (Widdel and Pfennig, 1984) and 1 ml l^−1^ of four vitamin solutions (prepared according to Aeckersberg *et al*., 1991). For measurement of SO_4_^2−^ evolution during S_2_O_3_^2−^ oxidation, 10 mmol l^−1^ Na_2_S_2_O_3_ · 5 H_2_O and 5 mmol l^−1^ glucose were added and 2 g l^−1^ K_2_SO_4_ from the original recipe was replaced with 0.75 g l^−1^ KCl. To compare growth between a culture with and without S_2_O_3_^2−^, K_2_SO_4_ was not omitted from the medium and glucose and Na_2_S_2_O_3_ · 5 H_2_O were used in the same concentrations as described above. To investigate the growth with 4-hydroxybenzoic acid and benzoate, both compounds were added in a concentration of 2 mmol l^−1^, respectively, without any other carbon source. DMSP utilization as the single carbon and electron source was tested using the CM medium, from which glucose has been omitted and 2 mmol l^−1^ DMSP were added. DMSP utilization as the single sulfur source was performed in the CM medium containing 2 mmol l^−1^ DMSP, 5 mmol l^−1^ glucose, and 0.75 g l^−1^ KCl instead of 2 g l^−1^ K_2_SO_4_. 2.1 g l^−1^ FeSO_4_ · 7 H_2_O and 0.14 g l^−1^ ZnSO_4_ · 7 H_2_O were replaced with 1.5 g l^−1^ FeCl_2_ · 4 H_2_O and 0.07 g l^−1^ ZnCl_2_ in the trace element solution respectively. Growth with phosphonoacetate (1 mmol l^−1^) as phosphorus source was tested by adding this compound as the only phosphorus source and all vitamins were omitted from the medium. For fermentation and denitrification experiments under anoxic conditions with strain FO-BEG1, aged and autoclaved North Sea water was buffered with 50 mmol l^−1^ TRIS, supplemented with NH_4_Cl (10 mmol l^−1^) and the pH adjusted to 8. We could not grow strain FO-BEG1 in the CM medium under anaerobic conditions, for so far unknown reasons, and therefore exchanged the deionized water with aged, autoclaved North Sea water. For strain JE062, the CM medium was used. Preparation of the anoxic medium was performed according to Widdel and Bak (1992). Cooling was performed under N_2_ atmosphere, except for experiments in which N_2_ production was monitored, in which Ar was used as the atmosphere instead. After autoclavation, the medium was supplemented with glucose (10 mmol l^−1^), 1 ml l^−1^ tungsten/selenium solution, 1 ml l^−1^ trace elements, 1 ml l^−1^ of four vitamin solutions, and 1 ml l^−1^ of the K_2_HPO_4_ solution as described above. When the North Sea medium was used, no K_2_HPO_4_ solution was added, but phosphate was supplied to the medium as the vitamins were dissolved in phosphate buffers. NaNO_3_ (10 mmol l^−1^) was added for experiments investigating denitrification. To test CO oxidation, CM medium was prepared as described above, containing 1 mmol l^−1^ glucose and supplied with 500 p.p.m. CO to the bottle headspace. To test for TDA production, both strains were grown in Difco 2216 marine broth media prepared according to the manufacturers instructions (BD Biosciences, Frankllin Lakes, NJ). For aerobic growth experiments, 250 ml Erlenmeyer flasks were filled with 100 ml medium. For anaerobic growth, 156 ml serum bottles (Wheaton, Millville, USA) were filled anoxically with 50 ml medium and closed with butyl rubber stoppers. For all experiments, 0.1% or 0.5% of the respective FO-BEG1 or JE062 preculture grown aerobically in CM medium was used as inoculum. All growth experiments were performed in triplicates at 28°C in the dark with shaking at 120 r.p.m.

### Chemical analyses

Bacterial growth was monitored as the OD_600_ using an Eppendorf BioPhotometer (Eppendorf AG, Hamburg, Germany). SO_4_^2−^ was measured with a Metrohm 761 Compact IC with conductivity detector (Metrohm AG, Herisau, Switzerland) equipped with a Metrosep A Supp 5–100 column with a carbonate eluent (3.2 mmol l^−1^ Na_2_CO_3_/1 mmol l^−1^ NaHCO_3_ in deionized water) at a flow rate of 0.7 ml min^−1^. Tetrathionate was measured according to Kamyshny (2009). Glucose and organic acids were determined using a high-performance liquid chromatography (HPLC) system (Sykam GmbH) equipped with an anion neutral pre-column (4 × 20 mm; Sykam GmbH) and an Aminex HPX-87H separation column (300 × 7.8 mm; Biorad, Munich, Germany) at a temperature of 60°C. The eluent consisted of 5 mmol l^−1^ H_2_SO_4_ in HPLC-grade water with a flow rate of 0.6 ml min^−1^. Quantification of glucose, succinate, lactate, formate, acetate, propionate and ethanol was performed with the 7515A RI detector (ERC, Riemerling, Germany); pyruvate was measured with the Sapphire UV-Vis detector at 210 nm (Ecom, Praha, Czech Republic). NO_3_^−^ was quantified with a HPLC system (Sykam GmbH, Eresing, Germany) containing an anion neutral pre-column (4 × 20 mm; Sykam GmbH) and an IBJ A3 anion separation column (4 × 60 mm; Sykam GmbH) with a column temperature of 50°C. The eluent consisted of 25 mmol l^−1^ NaCl and 45% ethanol in deionized water with a flow rate of 1 ml min^−1^. Detection of NO_3_^−^ was conducted with Linear Uvis 200 (Thermo Fischer Scientific GmbH, Dreieich, Germany) at 220 nm. N_2_ was measured as described by Zedelius and colleagues (2011). CO determination was conducted with a Shimadzu GC-8A (Shimadzu, Duisburg, Germany) gas chromatograph with a Molecular Sieve 5A column (80–100; 0.125 in. by 2 m; Restek, Bellefonte, USA) at a flow of 20 ml of synthetic air per minute at 40°C and an RGD2 reduction gas detector (Trace Analytical, Menlo Park, USA). Extraction and the RP-HPLC-based determination of the TDA content in the spent medium of both strains were performed by BioViotica Naturstoffe GmbH (Göttingen, Germany). Briefly, the samples where acidified to a pH of 3 with 2 mol l^−1^ HCl and 20 ml of each sample were extracted twice with 20 ml ethyl acetate. The ethyl acetate in the extracts was evaporated completely, the samples where resuspended in 1 ml acetonitrile, and analysed via a HPLC system with a Nucleodur 100-5 C18 ec (250 × 3 mm) column. The mobile phase consisted of A: deionized water with 0.1% trifluoroacetic acid (TFA) and B: acetonitrile with 0.1% (TFA) with a flow rate of 0.5 ml min^−1^. The gradient was the following: 0–25 min: 20% B to 100% B; 25–30 min: 100% B.

### DNA extraction and sequencing of strain FO-BEG1

DNA was extracted from strain FO-BEG1 using the Fast DNA SPIN Kit for Soil (MP Biomedicals LLC, Illkirch, France), according to manufacturers’ instructions. 454 sequencing was conducted by LGC Genomics GmbH with a 454 GS FLX System and resulted in 122 large (> 500 bp) contigs. A fosmid library was constructed in pCC1 and 192 clones have been end-sequenced. 96 gaps were closed via end-sequencing of fosmids and by 73 walking reads on selected fosmids. The remaining 25 gaps not covered by fosmids were amplified with combinatorial PCR using up to 8 primers per reaction. Gaps were closed by walking reactions on PCR products. All Sanger sequencing reactions were done using BigDye version 3.1 chemistry on a 3730XL Genetic Analyzer (Applied Biosystems, Life Technologies, Paisley, UK). The Newbler 2.0.00.22 software was used for sequence assembly and quality assessment. Overall, 522919 sequenced reads with an average length of 336.30 bp lead to a 29-fold sequence coverage.

### Gene prediction, annotation and data mining of strain FO-BEG1

Gene prediction was carried out by using the software Glimmer3 (Delcher *et al*., 2007). Ribosomal RNA genes were detected by using the RNAmmer 1.2 software (Lagesen *et al*., 2007) and transfer RNAs by tRNAscan-SE (Lowe and Eddy, 1997). Annotation was performed by using the GenDB, version 2.2 system (Meyer *et al*., 2003), supplemented by the tool JCoast, version 1.6 (Richter *et al*., 2008). For each predicted ORF observations have been collected from similarity searches against sequence databases NCBI-nr, Swiss-Prot, KEGG and genomesDB (Richter *et al*., 2008) and for protein family databases from Pfam (Bateman *et al*., 2004) and InterPro (Mulder *et al*., 2005). SignalP has been used for signal peptide predictions (Bendtsen *et al*., 2004) and TMHMM for transmembrane helix-analysis (Krogh *et al*., 2001). Predicted protein coding sequences were automatically annotated by the in-house software MicHanThi (Quast, 2006). The MicHanThi software predicts gene functions based on similarity searches using the NCBI-nr (including Swiss-Prot) and InterPro database. The annotation of proteins highlighted within the scope of this study was subject of manual inspection. For all observations regarding putative protein functions, an *expectation (E)*-value cut-off of 10^−4^ was considered.

### Comparison of the shared gene content by reciprocal best matches (RBMs)

Determination of the shared gene content has been performed by a blast all versus all search between FO-BEG1 and JE062. Reciprocal best matches were counted by a blast result with *E*-values of < 10^−5^ each and a subject coverage of over 65%.

### Functional classification with KEGG

For metabolic pathway identification, genes were searched for similarity against the KEGG database. A match was counted if the similarity search resulted in an expectation *E*-value below 10^−5^. All occurring KO (KEGG Orthology) numbers were mapped against KEGG pathway functional hierarchies and statistical analysed.

### Functional classification with COG

All predicted ORFs were also searched for similarity against the COGs database (Tatusov *et al*., 2003). A match was counted if the similarity search resulted in an *E*-value below 10^−5^.

### Average nucleotide identity

The ANI between the closed-genome sequences of strain FO-BEG1 and the draft-genome sequences of strain JE062 was determined by using the *in silico* DNA–DNA hybridization method of the JSpecies (Richter and Rosselló-Móra, 2009) software with default parameters.

### Creation of circular genome maps

Comparative circular genome maps of the RBMs shared between JE062 and FO-BEG1 have been drawn by using JCoast’s plugin for CGView (Stothard and Wishart, 2005). Circular GC-plot and GC-skew representation has been drawn by using DNAPlotter (Carver *et al*., 2009).

## Notes

Fig S1 Maximum likelihood tree including bootstrap values of 1000 runs of nearly full-length 16S rDNA sequences of the *Alphaproteobacteria* based on the study of Taylor and colleagues (2007), including the *Pseudovibrio* genus and the *Roseobacter* clade. The tree has been calculated with nucleotides at positions between 101 and 1405 according to *E. coli* numbering. Sequences belonging to the *Chloroflexaceae* have been used as out-group to root the tree. The number within the collapsed branches displays the number of sequences present in the respective cluster. The accession numbers of the *Pseudovibrio* sequences can be found in the tree in Fig. 1 and the accession numbers of the sequences affiliated with the marine *Roseobacter* clade are listed in Table S10. The bar represents 10% sequence divergence.

Fig S2 A. Growth of *Pseudovibrio* strain FO-BEG1 with aromatic compounds as the sole carbon and energy source. B. Growth and SO_4_^2-^ evolution by *Pseudovibrio* sp. FO-BEG1 under oxic conditions with the addition of 10 mmol l^-1^ Na_2_S_2_O_3_ to the medium. Closed squares and circles represent the SO_4_^2-^ evolution and optical density in inoculated samples. Open squares and open circles represent SO_4_^2-^ evolution and optical density, respectively, in an uninoculated control to exclude chemical oxidation of Na_2_S_2_O_3_. Initially, the medium did not contain any SO_4_^2-^ in order to decrease the SO_4_^2-^ background during measurements. C. Growth of *Pseudovibrio* sp. FO-BEG1 with and without the addition of 10 mmol l^-1^ Na_2_S_2_O_3_ to the medium. The medium for this experiment contains 11.5 mmol l^-1^ K_2_SO_4_ to ensure that the culture without Na_2_S_2_O_3_ contains a sulfur source for growth. Error bars represent the standard deviation in biological triplicates.

Fig S3 A. Glucose consumption, growth and production of fermentation products by *Pseudovibrio* sp. FO-BEG1 grown under anoxic conditions without NO_3_^-^. B. Glucose consumption, growth and production of fermentation products during simultaneous denitrification and fermentation by *Pseudovibrio* sp. FO-BEG1 grown under anoxic conditions with the addition of 10 mmol l^-1^ NO_3_^-^. C. Consumption of NO_3_^-^, evolution of gaseous nitrogen and growth during simultaneous denitrification and fermentation of *Pseudovibrio* sp. FO-BEG1 with 10 mmol l^-1^ NO_3_^-^. Error bars represent the standard deviation in biological triplicates.

Fig S4 A. Growth of *Pseudovibrio* sp. FO-BEG1 with 1 mmol l^-1^ phosphonoacetate as the only phosphorus source and without the addition of any phosphorus to the medium. B. Growth of *Pseudovibrio* sp. FO-BEG1 with and without the addition of vitamins to the medium. C. Growth of *Pseudovibrio* sp. FO-BEG1 and JE062 with 2 mmol l^-1^ DMSP as the single carbon and electron source and with 2 mmol l^-1^ DMSP, 5 mmol l^-1^ glucose and without SO_4_^2-^ to test whether the DMSP can be used as the single sulfur source. Error bars represent the standard deviation in biological triplicates.

Fig S5 A. Growth of *Pseudovibrio* strain JE062 with aromatic compounds as the sole carbon and energy source. B. Growth and SO_4_^2-^ evolution by *Pseudovibrio* sp. JE062 under oxic conditions with the addition of 10 mmol l^-1^ Na_2_S_2_O_3_ to the medium. Initially, the medium did not contain any SO_4_^2-^ in order to decrease the SO_4_^2-^ background during measurements. C. Growth of *Pseudovibrio* sp. JE062 with and without the addition of 10 mmol l^-1^ Na_2_S_2_O_3_ to the medium. The medium for this experiment contains 11.5 mmol l^-1^ K_2_SO_4_ to ensure that the culture without Na_2_S_2_O_3_ contains a sulfur source for growth. Error bars represent the standard deviation in biological triplicates.

Fig S6 A. Glucose consumption, growth and production of fermentation products by *Pseudovibrio* sp. JE062 grown under anoxic conditions without NO_3_^-^. B. Glucose consumption, growth and production of fermentation products during simultaneous denitrification and fermentation by *Pseudovibrio* sp. JE062 grown under anoxic conditions with the addition of 10 mmol l^-1^ NO_3_^-^. C. Growth, consumption of NO_3_^-^ and production of N_2_ and NO_2_^-^ during simultaneous denitrification and fermentation of *Pseudovibrio* sp. JE062 with 10 mmol l^-1^ NO_3_^-^. The incomplete utilization of NO_3_^-^ and the production of a small amount NO_2_^-^ in the stationary growth phase can most likely be attributed to the strong acidification of the medium (pH measured at the last day of sampling was 5.8). Error bars represent the standard deviation in biological triplicates.

Fig S7 A. Growth of *Pseudovibrio* sp. JE062 with 1 mmol l^-1^ phosphonoacetate as the only phosphorus source and without the addition of any phosphorus to the medium. B. Growth of *Pseudovibrio* sp. JE062 with and without the addition of vitamins to the medium. C. Growth of *Pseudovibrio* sp. JE062 under oligotrophic conditions. Error bars represent the standard deviation in biological triplicates.

Fig S8 Comparison of genes encoding for amyloids in *Enterobacteriaceae* and the operon in *Pseudovibrio* sp. FO-BEG1. White arrows represent homologues of genes in enteric bacteria; gray arrows show genes present in *Enterobacteriaceae* only; black arrows show genes containing curli repeats, typical motifs of the amyloid structural subunits. The number within the black arrows shows the amount of curli repeats in the according gene.

Table S1 Categorization of the genes of *Pseudovibrio* sp. FO-BEG1 and JE062 into COG categories.

**Table S2.** Genes detected in the genomes of *Pseudovibrio* sp. FO-BEG1 and JE062 coding for predicted proteins involved in carbon metabolism, denitrification, oxidation of thiosulfate, and phosphonate utilization. Genes that could not be detected in the not closed genome of strain JE062 are indicated with ‘–’. Absence of a gene name or an EC number indicates that no assignment was made due to missing of these parameters for the respective genes. **Table S3.** Genes detected in the genomes of *Pseudovibrio* sp. FO-BEG1 and JE062 coding for predicted TRAP transporter subunits. Genes that could not be detected in the not closed genome of strain JE062 are indicated with ‘–’. **Table S4.** Genes detected in the genomes of *Pseudovibrio* sp. FO-BEG1 and JE062 coding for predicted ABC transporter subunits. Genes that could not be detected in the not closed genome of strain JE062 are indicated with ‘–’. Pfam model specifies the family, to which the identified protein belongs, according with the Pfam database (Bateman *et al*., 2004). Absence of a gene name indicates that no assignment was made due to missing of this parameter for the respective genes. Predicted substrate specificity was derived from the annotations of the genes belonging to the respective ABC transporter system. **Table S5.** Genes detected in the genomes of *Pseudovibrio* sp. FO-BEG1 and JE062 coding for predicted peptidases/proteases and proteins involved in protection against reactive oxygen species (ROS). Genes that could not be detected in the not closed genome of strain JE062 are indicated with ‘–’. Absence of a gene name or an EC number indicates that no assignment was made due to missing of these parameters for the respective genes. **Table S6.** Genes detected in the genomes of *Pseudovibrio* sp. FO-BEG1 and JE062 coding for predicted proteins involved vitamin synthesis. Genes that could not be detected in the not closed genome of strain JE062 are indicated with ‘–’. Absence of a gene name or an EC number indicates that no assignment was made due to missing of these parameters for the respective genes. **Table S7.** Genes detected in the genomes of *Pseudovibrio* sp. FO-BEG1 and JE062 coding for predicted integrases, transposases, subunits of the gene transfer agent (GTA) and genes of the NRPS/PKS cluster as well as the *tdaA-tdaF* locus involved in tropodithietic acid (TDA) synthesis. Genes that could not be detected in the not closed genome of strain JE062 are indicated with ‘–’. Absence of a gene name indicates that no assignment was made due to missing of this parameter for the respective genes. For the NRPS/PKS cluster, sequence similarity to the genes of the colibactin producing *E. coli* CFT073 strain is given. **Table S8.** Genes detected in the genomes of *Pseudovibrio* sp. FO-BEG1 and JE062 coding for predicted proteins presumably involved in attachment, protein-protein interactions and predicted LuxR transcriptional regulators. Genes that could not be detected in the not closed genome of strain JE062 are indicated with ‘–’. For *luxR* homologues, the presence of an amino-terminal autoinducer-binding or response regulatory domain is indicated with a ‘+’. Absence of a gene name or an EC number indicates that no assignment was made due to missing of these parameters for the respective genes. **Table S9.** Genes detected in the genomes of *Pseudovibrio* sp. FO-BEG1 and JE062 coding for predicted subunits of the type III and type VI secretion systems. Genes that could not be detected in the not closed genome of strain JE062 are indicated with ‘–’. Absence of a gene name indicates that no assignment was made due to missing of this parameter for the respective genes. For the type III secretion system, sequence similarity to the best SwissProt hit is given.

Table S10 Sequences associated with the *Roseobacter* cluster that have been used for the calculation of the phylogenetic tree.
