# Supplementary figures and images for: The genus Pseudovibrio contains metabolically versatile bacteria adapted for symbiosis
Source: Environ Microbiol. 2013 Apr 18;15(7):2095–113. doi: 10.1111/1462-2920.12123 (PMC3806328; doi:10.1111/1462-2920.12123)

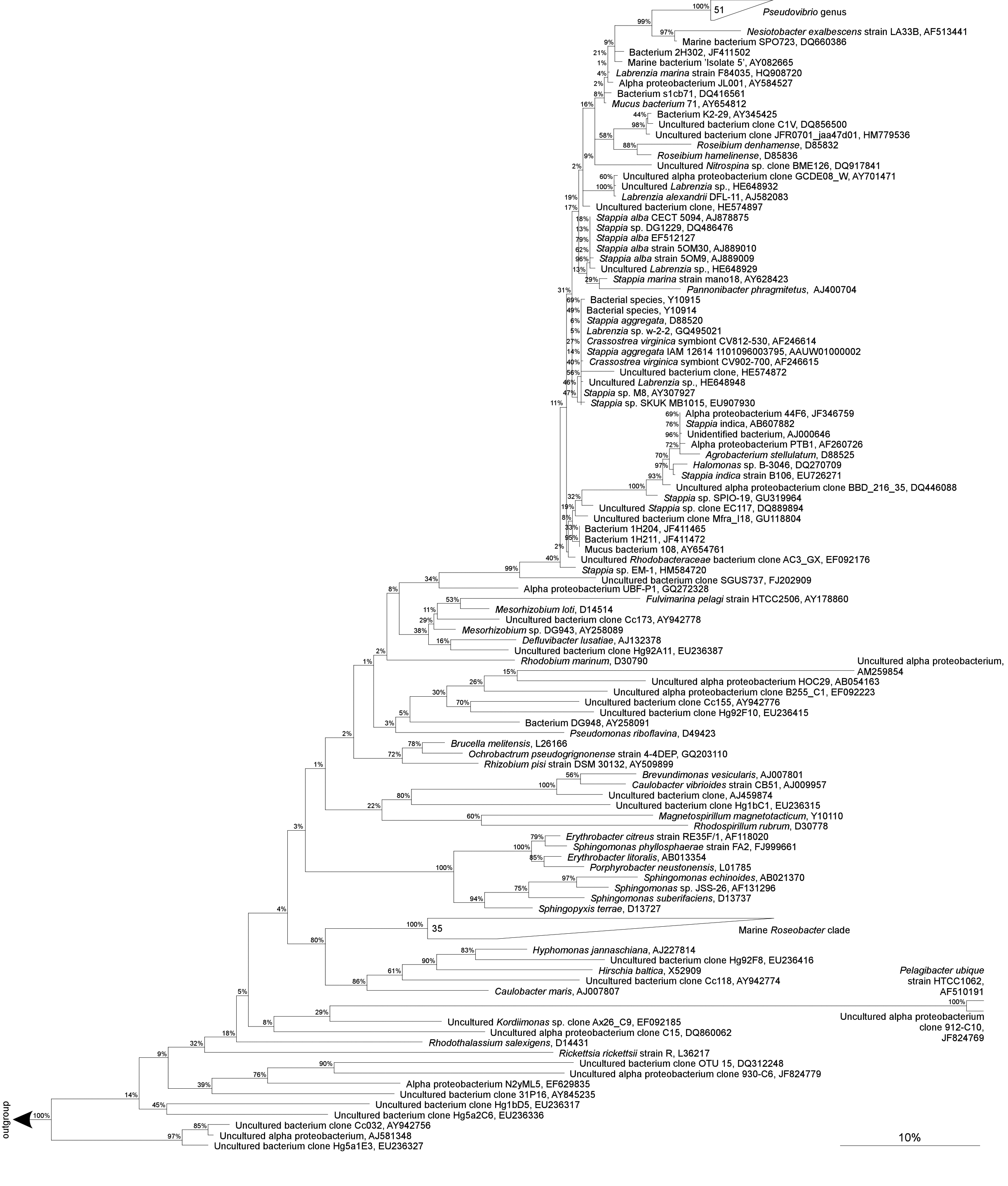

Supplement: Fig S1 — Maximum likelihood tree including bootstrap values of 1000 runs of nearly full-length 16S rDNA sequences of the Alphaproteobacteria based on the study of Taylor and colleagues (2007), including the Pseudovibrio genus and the Roseobacter clade. The tree has been calculated with nucleotides at positions between 101 and 1405 according to E. coli numbering. Sequences belonging to the Chloroflexaceae have been used as out-group to root the tree. The number within the collapsed branches displays the number of sequences present in the respective cluster. The accession numbers of the Pseudovibrio sequences can be found in the tree in Fig. 1 and the accession numbers of the sequences affiliated with the marine Roseobacter clade are listed in Table S10. The bar represents 10% sequence divergence. [file emi0015-2095-sd1.tif]

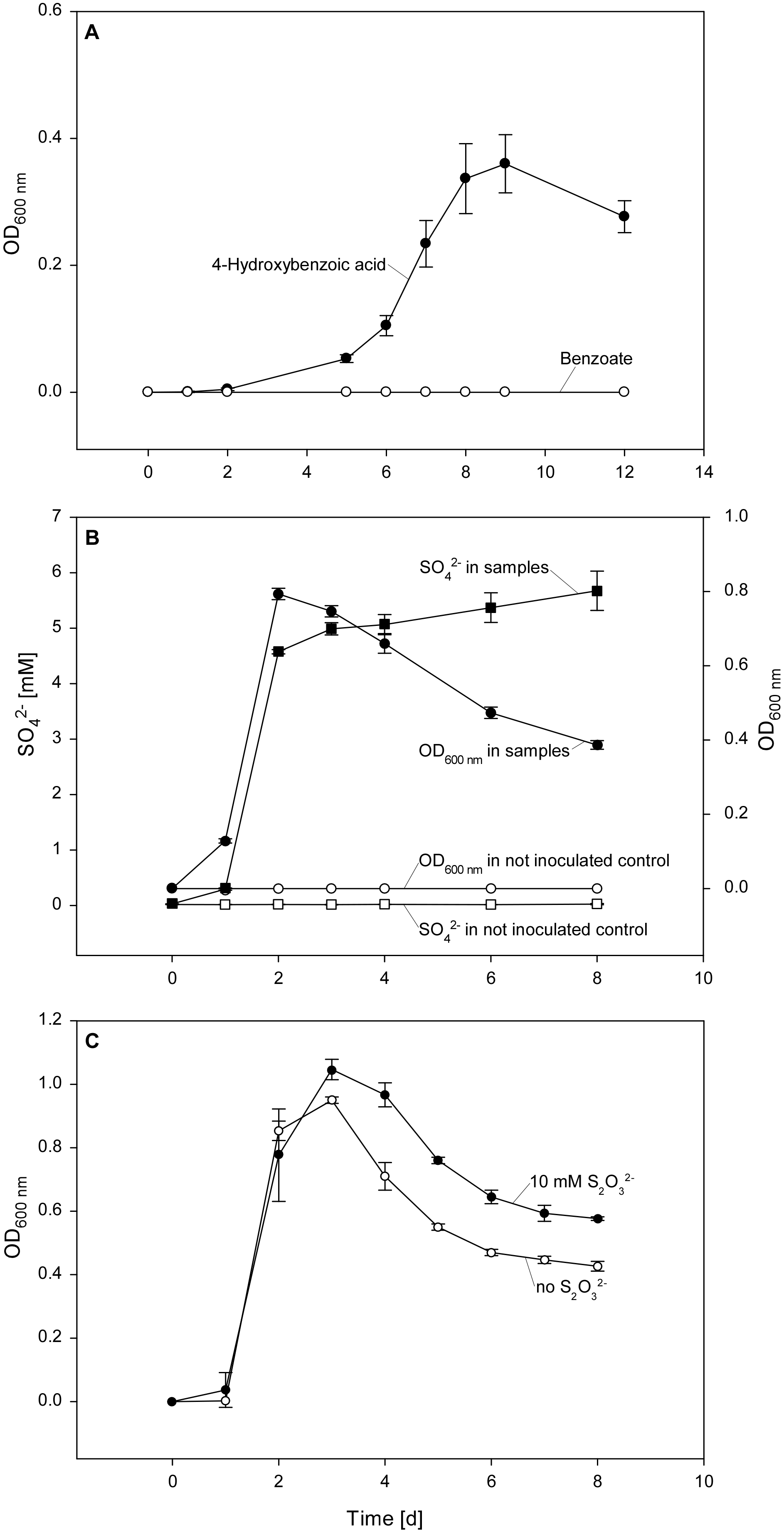

Supplement: Fig S2 — A. Growth of Pseudovibrio strain FO-BEG1 with aromatic compounds as the sole carbon and energy source. B. Growth and SO42- evolution by Pseudovibrio sp. FO-BEG1 under oxic conditions with the addition of 10 mmol l-1 Na2S2O3 to the medium. Closed squares and circles represent the SO42- evolution and optical density in inoculated samples. Open squares and open circles represent SO42- evolution and optical density, respectively, in an uninoculated control to exclude chemical oxidation of Na2S2O3. Initially, the medium did not contain any SO42- in order to decrease the SO42- background during measurements. C. Growth of Pseudovibrio sp. FO-BEG1 with and without the addition of 10 mmol l-1 Na2S2O3 to the medium. The medium for this experiment contains 11.5 mmol l-1 K2SO4 to ensure that the culture without Na2S2O3 contains a sulfur source for growth. Error bars represent the standard deviation in biological triplicates. [file emi0015-2095-sd2.tif]

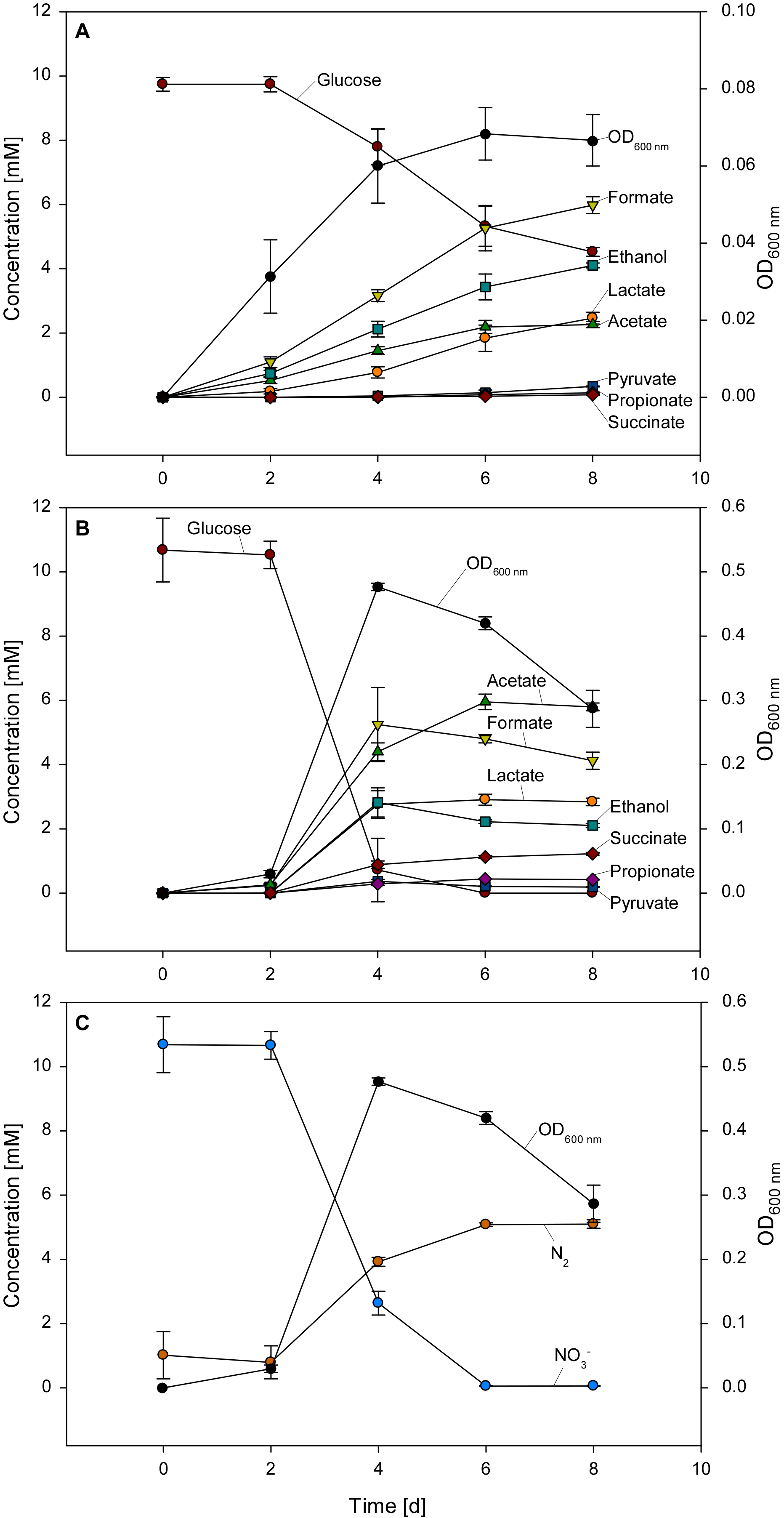

Supplement: Fig S3 — A. Glucose consumption, growth and production of fermentation products by Pseudovibrio sp. FO-BEG1 grown under anoxic conditions without NO3-. B. Glucose consumption, growth and production of fermentation products during simultaneous denitrification and fermentation by Pseudovibrio sp. FO-BEG1 grown under anoxic conditions with the addition of 10 mmol l-1 NO3-. C. Consumption of NO3-, evolution of gaseous nitrogen and growth during simultaneous denitrification and fermentation of Pseudovibrio sp. FO-BEG1 with 10 mmol l-1 NO3-. Error bars represent the standard deviation in biological triplicates. [file emi0015-2095-sd3.tif]

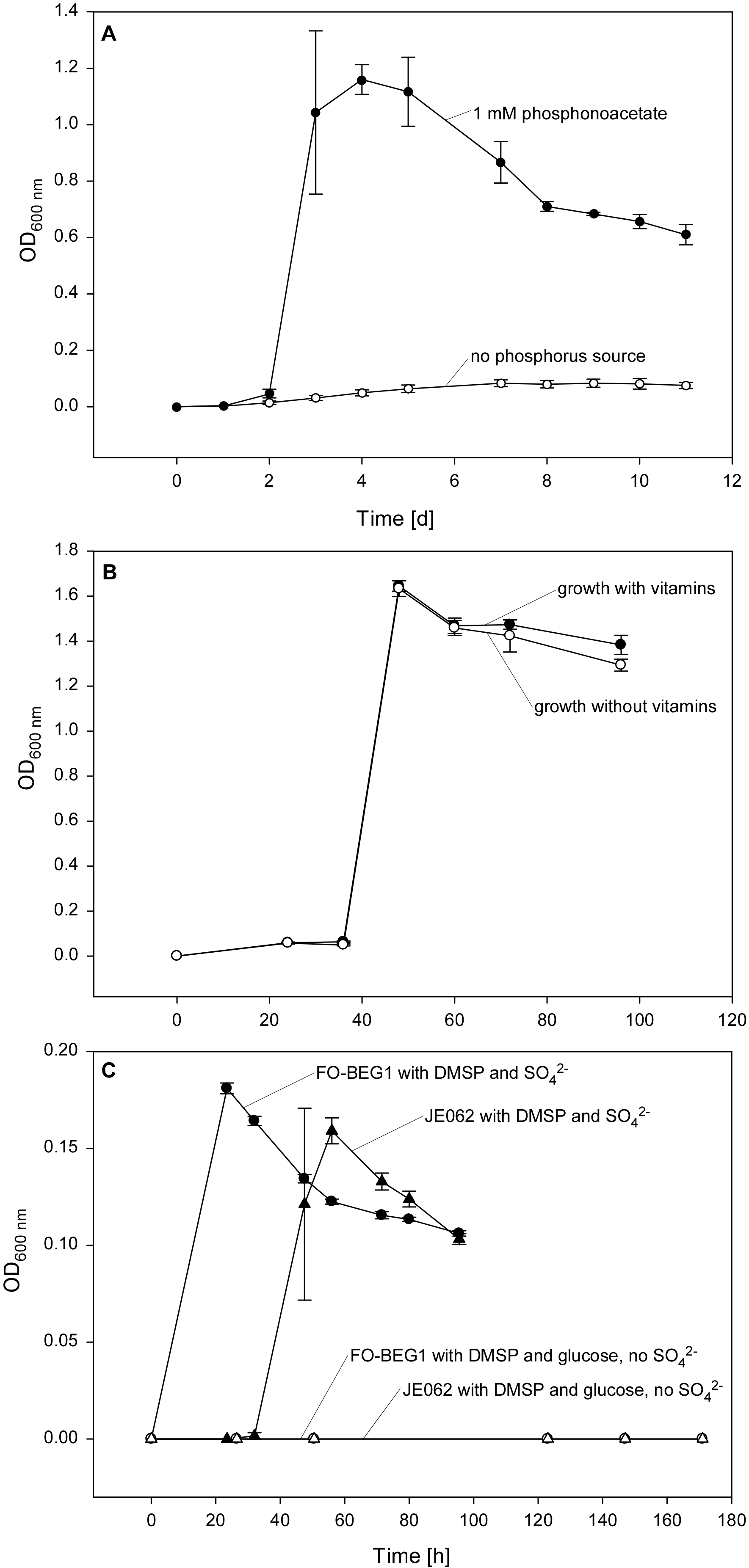

Supplement: Fig S4 — A. Growth of Pseudovibrio sp. FO-BEG1 with 1 mmol l-1 phosphonoacetate as the only phosphorus source and without the addition of any phosphorus to the medium. B. Growth of Pseudovibrio sp. FO-BEG1 with and without the addition of vitamins to the medium. C. Growth of Pseudovibrio sp. FO-BEG1 and JE062 with 2 mmol l-1 DMSP as the single carbon and electron source and with 2 mmol l-1 DMSP, 5 mmol l-1 glucose and without SO42- to test whether the DMSP can be used as the single sulfur source. Error bars represent the standard deviation in biological triplicates. [file emi0015-2095-sd4.tif]

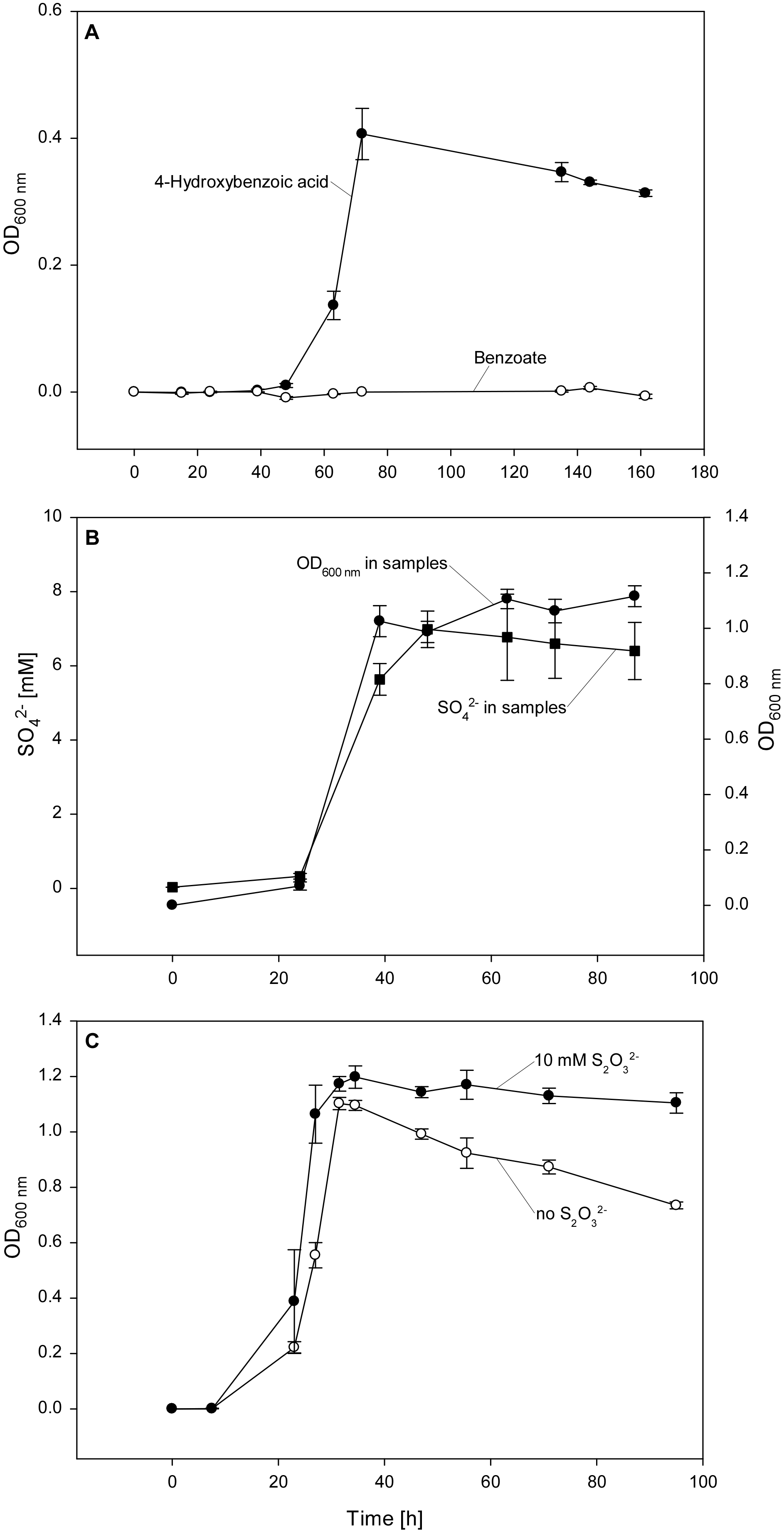

Supplement: Fig S5 — A. Growth of Pseudovibrio strain JE062 with aromatic compounds as the sole carbon and energy source. B. Growth and SO42- evolution by Pseudovibrio sp. JE062 under oxic conditions with the addition of 10 mmol l-1 Na2S2O3 to the medium. Initially, the medium did not contain any SO42- in order to decrease the SO42- background during measurements. C. Growth of Pseudovibrio sp. JE062 with and without the addition of 10 mmol l-1 Na2S2O3 to the medium. The medium for this experiment contains 11.5 mmol l-1 K2SO4 to ensure that the culture without Na2S2O3 contains a sulfur source for growth. Error bars represent the standard deviation in biological triplicates. [file emi0015-2095-sd5.tif]

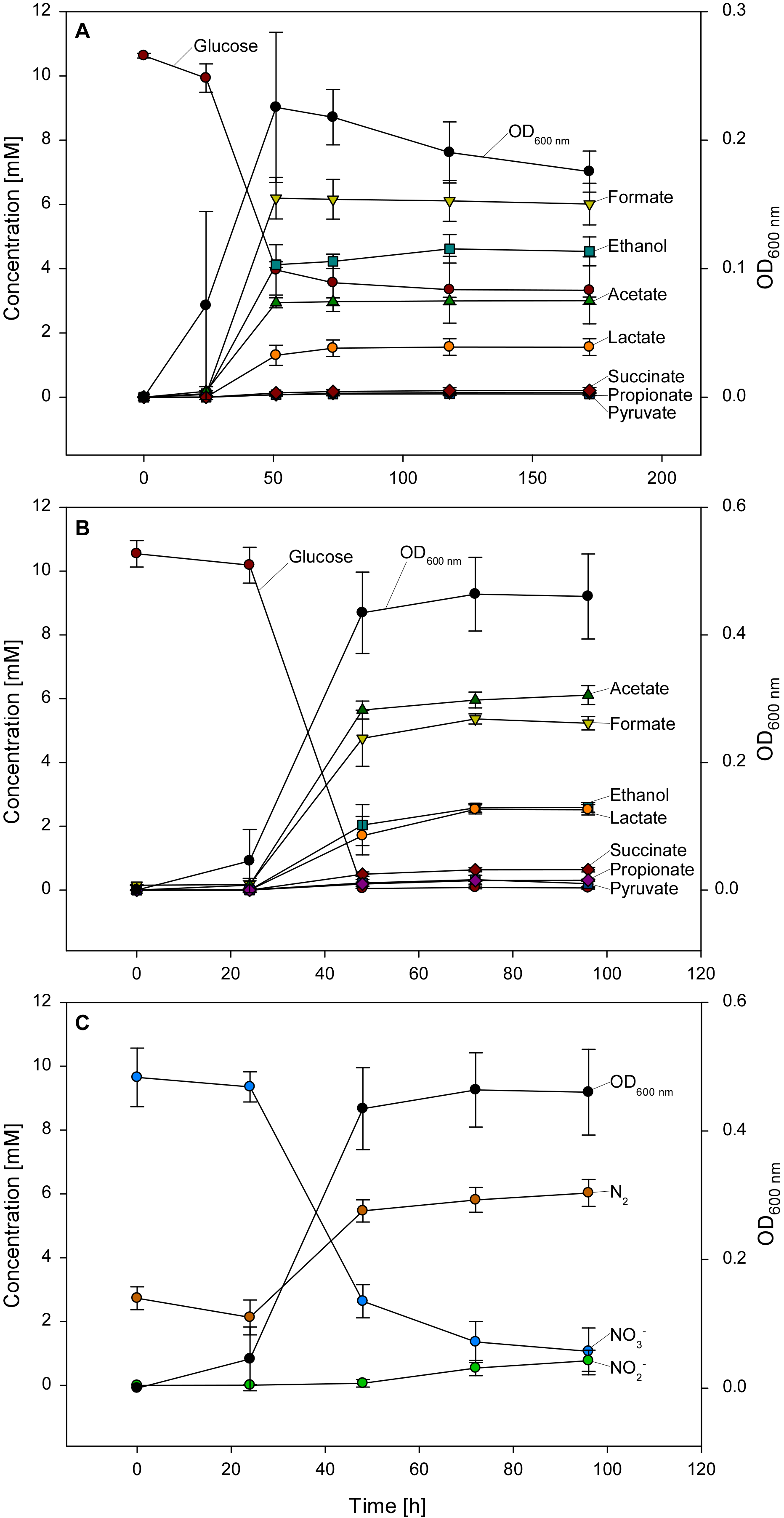

Supplement: Fig S6 — A. Glucose consumption, growth and production of fermentation products by Pseudovibrio sp. JE062 grown under anoxic conditions without NO3-. B. Glucose consumption, growth and production of fermentation products during simultaneous denitrification and fermentation by Pseudovibrio sp. JE062 grown under anoxic conditions with the addition of 10 mmol l-1 NO3-. C. Growth, consumption of NO3- and production of N2 and NO2- during simultaneous denitrification and fermentation of Pseudovibrio sp. JE062 with 10 mmol l-1 NO3-. The incomplete utilization of NO3- and the production of a small amount NO2- in the stationary growth phase can most likely be attributed to the strong acidification of the medium (pH measured at the last day of sampling was 5.8). Error bars represent the standard deviation in biological triplicates. [file emi0015-2095-sd6.tif]

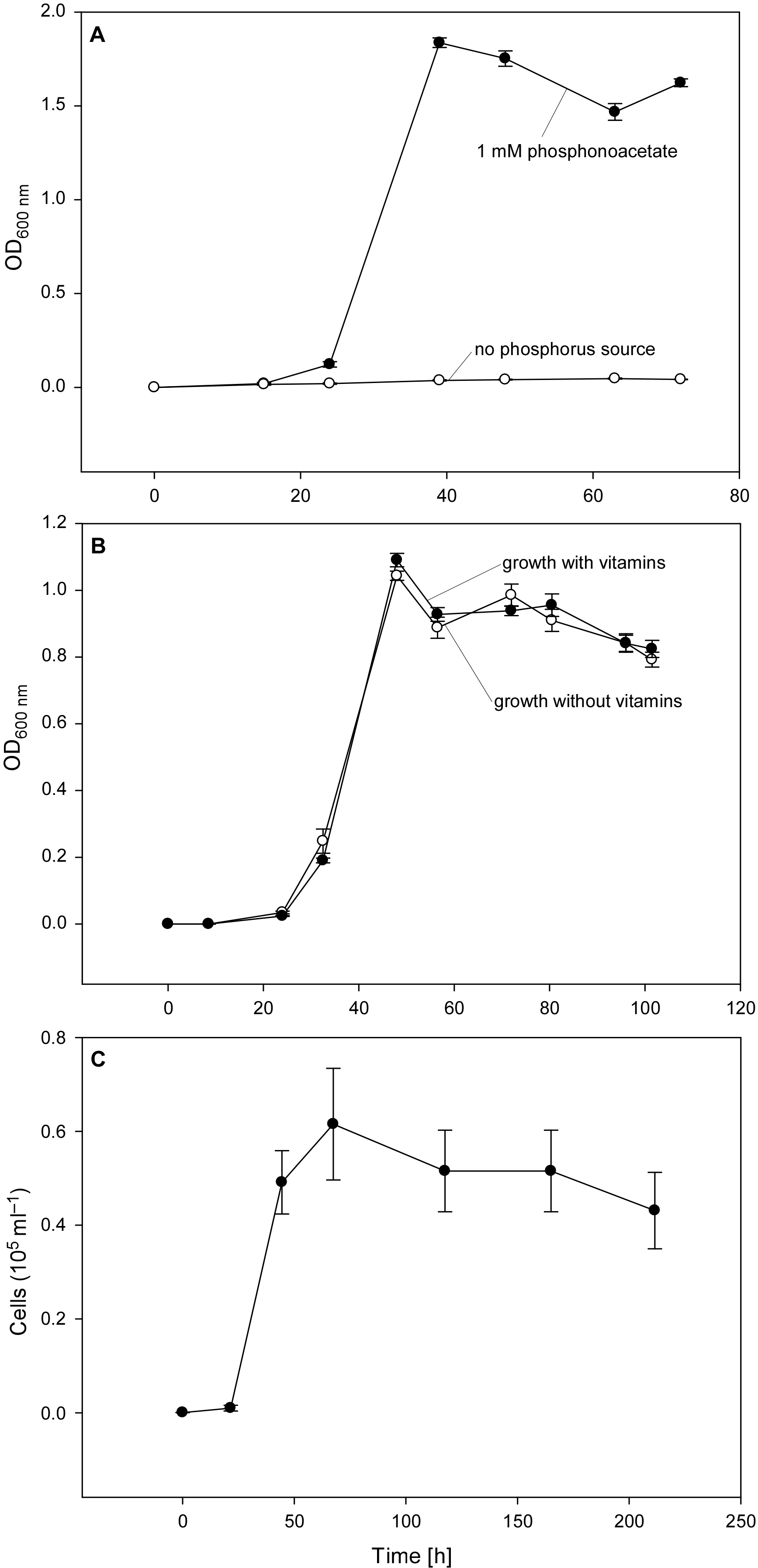

Supplement: Fig S7 — A. Growth of Pseudovibrio sp. JE062 with 1 mmol l-1 phosphonoacetate as the only phosphorus source and without the addition of any phosphorus to the medium. B. Growth of Pseudovibrio sp. JE062 with and without the addition of vitamins to the medium. C. Growth of Pseudovibrio sp. JE062 under oligotrophic conditions. Error bars represent the standard deviation in biological triplicates. [file emi0015-2095-sd7.tif]

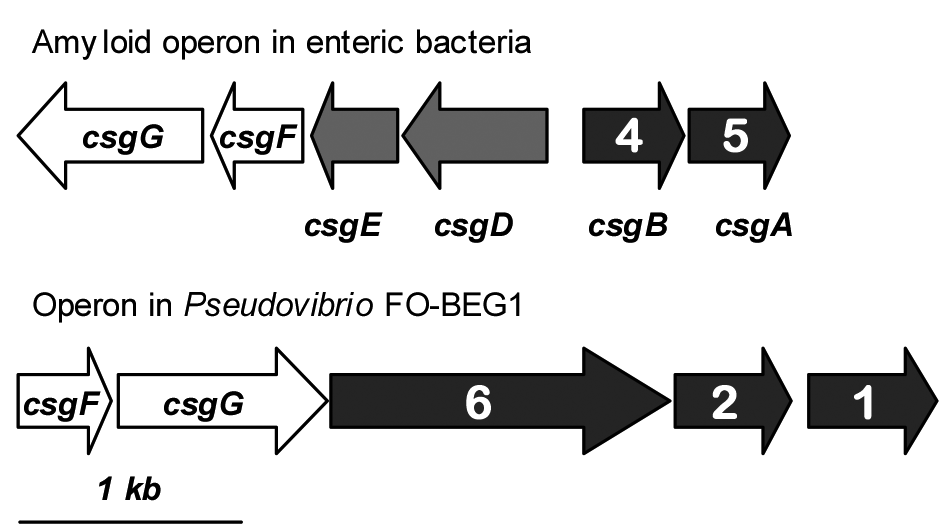

Supplement: Fig S8 — Comparison of genes encoding for amyloids in Enterobacteriaceae and the operon in Pseudovibrio sp. FO-BEG1. White arrows represent homologues of genes in enteric bacteria; gray arrows show genes present in Enterobacteriaceae only; black arrows show genes containing curli repeats, typical motifs of the amyloid structural subunits. The number within the black arrows shows the amount of curli repeats in the according gene. [file emi0015-2095-sd8.tif]
