# Supplementary material for: The genus Pseudovibrio contains metabolically versatile bacteria adapted for symbiosis
Source: Environ Microbiol. 2013 Apr 18;15(7):2095–113. doi: 10.1111/1462-2920.12123 (PMC3806328; doi:10.1111/1462-2920.12123)
Supplement: Table S1 — Categorization of the genes of Pseudovibrio sp. FO-BEG1 and JE062 into COG categories. [file emi0015-2095-sd9.doc]

**Table S1:** Categorization of the genes of *Pseudovibrio* sp. FO-BEG1 and JE062 into cluster of orthologous group (COG) categories.

| **COG category** | **FO-BEG1** | **JE062** |
| --- | --- | --- |
| [J] Translation, ribosomal structure and biogenesis | 196 | 190 |
| [K] Transcription | 387 | 367 |
| [L] Replication, recombination and repair | 135 | 125 |
| [D] Cell cycle control, cell division, chromosome partitioning | 21 | 20 |
| [T] Signal transduction mechanism | 138 | 140 |
| [M] Cell wall/membrane/envelope biogenesis | 190 | 176 |
| [N] Cell motility | 153 | 149 |
| [O] Posttranslational modification, protein turnover, chaperones | 135 | 127 |
| [C] Energy production and conversion | 245 | 245 |
| [G] Carbohydrate transport and metabolism | 323 | 311 |
| [E] Amino acid transport and metabolism | 507 | 492 |
| [F] Nucleotide transport and metabolism | 99 | 92 |
| [H] Coenzyme transport and metabolism | 185 | 181 |
| [I] Lipid transport and metabolism | 148 | 142 |
| [P] Inorganic ion transport and metabolism | 291 | 287 |
| [Q] Secondary metabolites biosynthesis, transport and catabolism | 210 | 204 |
| [R] General function prediction only | 598 | 576 |
| [S] Function unknown | 281 | 272 |
