# Supplementary material for: The genus Pseudovibrio contains metabolically versatile bacteria adapted for symbiosis
Source: Environ Microbiol. 2013 Apr 18;15(7):2095–113. doi: 10.1111/1462-2920.12123 (PMC3806328; doi:10.1111/1462-2920.12123)
Supplement: Supplementary file 10 — Table S2. Genes detected in the genomes of Pseudovibrio sp. FO-BEG1 and JE062 coding for predicted proteins involved in carbon metabolism, denitrification, oxidation of thiosulfate, and phosphonate utilization. Genes that could not be detected in the not closed genome of strain JE062 are indicated with ‘–’. Absence of a gene name or an EC number indicates that no assignment was made due to missing of these parameters for the respective genes. Table S3. Genes detected in the genomes of Pseudovibrio sp. FO-BEG1 and JE062 coding for predicted TRAP transporter subunits. Genes that could not be detected in the not closed genome of strain JE062 are indicated with ‘–’. Table S4. Genes detected in the genomes of Pseudovibrio sp. FO-BEG1 and JE062 coding for predicted ABC transporter subunits. Genes that could not be detected in the not closed genome of strain JE062 are indicated with ‘–’. Pfam model specifies the family, to which the identified protein belongs, according with the Pfam database (Bateman et al., 2004). Absence of a gene name indicates that no assignment was made due to missing of this parameter for the respective genes. Predicted substrate specificity was derived from the annotations of the genes belonging to the respective ABC transporter system. Table S5. Genes detected in the genomes of Pseudovibrio sp. FO-BEG1 and JE062 coding for predicted peptidases/proteases and proteins involved in protection against reactive oxygen species (ROS). Genes that could not be detected in the not closed genome of strain JE062 are indicated with ‘–’. Absence of a gene name or an EC number indicates that no assignment was made due to missing of these parameters for the respective genes. Table S6. Genes detected in the genomes of Pseudovibrio sp. FO-BEG1 and JE062 coding for predicted proteins involved vitamin synthesis. Genes that could not be detected in the not closed genome of strain JE062 are indicated with ‘–’. Absence of a gene name or an EC number indicates that no ass [file emi0015-2095-sd10.doc]

**Table S 2.** Genes detected in the genomes of *Pseudovibrio* sp. FO-BEG1 and JE062 coding for predicted proteins involved in carbon metabolism, denitrification, oxidation of thiosulfate, and phosphonate utilization. Genes that could not be detected in the not closed genome of strain JE062 are indicated with ‘─’. Absence of a gene name or an EC number indicates that no assignment was made due to missing of these parameters for the respective genes.

| **Locus  FO-BEG1** | **Locus JE062** | **Product** | | **Gene name** | | **EC number** |
| --- | --- | --- | --- | --- | --- | --- |
|  |  | **Glycolysis / Gluconeogenesis** | |  | |  |
| PSE_0520 | PJE062_2836 | 2,3-bisphosphoglycerate-dependent phosphoglycerate mutase | | *gpmA* | | 5.4.2.1 |
| PSE_0524 | PJE062_2549 | 2,3-bisphosphoglycerate-dependent phosphoglycerate mutase | | *gpmA* | | 5.4.2.1 |
| PSE_1032 | PJE062_4521 | Glucokinase | | *glk* | | 2.7.1.2 |
| PSE_2344 | PJE062_1398 | Fructose-bisphosphate aldolase | | *fbaA* | | 4.1.2.13 |
| PSE_2526 | PJE062_127 | Phosphomannomutase/phosphoglucomutase | | *algC* | | 5.4.2.2 |
| PSE_3200 | PJE062_1722 | Enolase/2-phosphoglycerate dehydratase | | *eno* | | 4.2.1.11 |
| PSE_3383 | PJE062_1304 | Triose-phosphate isomerase | | *tpiA* | | 5.3.1.1 |
| PSE_4148 | PJE062_4782 | Glucose-6-phosphate isomerase | | *gpi* | | 5.3.1.9 |
| PSE_4697 | PJE062_2190 | Glyceraldehyde-3-phosphate dehydrogenase B | | *gapB/epd* | | 1.2.1.12 |
| PSE_4698 | PJE062_2252 | Phosphoglycerate kinase | | *pgk* | | 2.7.2.3 |
| PSE_4767 | PJE062_2266 | Pyruvate kinase | | *pyk* | | 2.7.1.40 |
| PSE_3532 | PJE062_1635 | Fructose-1,6-bisphosphatase class II | | *glpX* | | 3.1.3.37 |
| PSE_0639 | PJE062_2867 | Phosphoenolpyruvate carboxykinase | | *pckA* | | 4.1.1.49 |
| PSE_4866 | PJE062_2056 | Pyruvate carboxylase | | *pyc* | | 6.4.1.1 |
|  |  |  | |  | |  |
|  |  | **Entner-Doudoroff pathway** | |  | |  |
| PSE_0520 | PJE062_2836 | 2,3-bisphosphoglycerate-dependent phosphoglycerate mutase | | *gpmA* | | 5.4.2.1 |
| PSE_0524 | PJE062_2549 | 2,3-bisphosphoglycerate-dependent phosphoglycerate mutase | | *gpmA* | | 5.4.2.1 |
| PSE_1300 | PJE062_1936 | SMP-30/Gluconolaconase/LRE-like region | |  | | 3.1.1.17 |
| PSE_1664 | PJE062_417 | oxidoreductase, short chain dehydrogenase/reductase family protein | | *gdh* | | 1.1.1.47 |
| PSE_1887 | PJE062_3941 | SMP-30/Gluconolaconase/LRE-like region | |  | | 3.1.1.17 |
| PSE_1922 | PJE062_733 | Gluconokinase (Gluconate kinase) | | *gntK* | | 2.7.1.12 |
| PSE_3200 | PJE062_1722 | Enolase/2-phosphoglycerate dehydratase | | *eno* | | 4.2.1.11 |
| PSE_3907 | ─ | KDPG and KHG aldolase | | *eda* | | 4.1.2.14 |
| PSE_3909 | ─ | 2-dehydro-3-deoxygluconokinase | | *kdgK* | | 2.7.1.45 |
| PSE_p0106 | PJE062_762 | 2-dehydro-3-deoxygluconokinase | | *kdgK* | | 2.7.1.45 |
| PSE_p0137 | PJE062_797 | 2-dehydro-3-deoxygluconokinase | | *kdgK* | | 2.7.1.45 |
| PSE_4145 | PJE062_4650 | Glucose-6-phosphate 1-dehydrogenase | | *zwf* | | 1.1.1.49 |
| PSE_4146 | PJE062_4945 | Phosphogluconate dehydratase (6-phosphogluconate dehydratase) | | *edd* | | 4.2.1.12 |
| PSE_4147 | PJE062_4760 | KDPG and KHG aldolase | | *eda* | | 4.1.2.14 |
| PSE_4148 | PJE062_4782 | Glucose-6-phosphate isomerase | | *gpi* | | 5.3.1.9 |
| PSE_4154 | PJE062_4973 | SMP-30/Gluconolaconase/LRE-like region | |  | | 3.1.1.17 |
| PSE_4697 | PJE062_2190 | Glyceraldehyde-3-phosphate dehydrogenase B | | *gapB/epd* | | 1.2.1.12 |
| PSE_4698 | PJE062_2252 | Phosphoglycerate kinase | | *pgk* | | 2.7.2.3 |
| PSE_4767 | PJE062_2266 | Pyruvate kinase | | *pyk* | | 2.7.1.40 |
|  |  |  | |  | |  |
|  |  |  | |  | |  |
|  |  |  | |  | |  |
|  |  |  | |  | |  |
| **Table S 2.** Continued |  |  | |  | |  |
| **Locus  FO-BEG1** | **Locus  JE062** | **Product** | | **Gene** | | **EC** |
|  |  | **Pentose phosphate pathway** | |  | |  |
| PSE_0476 | PJE062_2965 | 6-phosphogluconate dehydrogenase | | *gnd* | | 1.1.1.44 |
| PSE_0628 | PJE062_3035 | Translaldolase | | *tal* | | 2.2.1.2 |
| PSE_1044 | PJE062_4442 | Phosphopentomutase | | *deoB* | | 5.4.2.7 |
| PSE_1456 | ─ | Fructose-bisphosphate aldolase | | *fbaA* | | 4.1.2.13 |
| PSE_2114 | PJE062_647 | Fructose-bisphosphate aldolase | | *fbaA* | | 4.1.2.13 |
| PSE_2344 | PJE062_1398 | Fructose-bisphosphate aldolase | | *fbaA* | | 4.1.2.13 |
| PSE_2648 | PJE062_4217 | Ribose-5-phosphate isomerase A | | *rpiA* | | 5.3.1.6 |
| PSE_2884 | PJE062_1466 | Ribokinase | | *rbsK* | | 2.7.1.15 |
| PSE_3532 | PJE062_1635 | Fructose-1,6-bisphosphatase class II | | *glpX* | | 3.1.3.11 |
| PSE_4036 | PJE062_4566 | Ribulose-phosphate 3-epimerase | | *rpe* | | 5.1.3.1 |
| PSE_4437 | PJE062_4652 | Ribose-phosphate pyrophosphokinase | | *prs* | | 2.7.6.1 |
| PSE_4696 | PJE062_2124 | Transketolase | | *tkt* | | 2.2.1.1 |
| PSE_1664 | PJE062_417 | oxidoreductase, short chain dehydrogenase/reductase family protein | | *gdh* | | 1.1.1.47 |
| PSE_1300 | PJE062_1936 | SMP-30/Gluconolaconase/LRE-like region | |  | | 3.1.1.17 |
| PSE_1887 | PJE062_3941 | SMP-30/Gluconolaconase/LRE-like region | |  | | 3.1.1.17 |
| PSE_4154 | PJE062_4973 | SMP-30/Gluconolaconase/LRE-like region | |  | | 3.1.1.17 |
| PSE_1922 | PJE062_733 | Gluconokinase (Gluconate kinase) | | *gntK* | | 2.7.1.12 |
| PSE_4148 | PJE062_4782 | Glucose-6-phosphate isomerase | | *gpi* | | 5.3.1.9 |
| PSE_4145 | PJE062_4650 | Glucose-6-phosphate 1-dehydrogenase | | *zwf* | | 1.1.1.49 |
|  |  |  | |  | |  |
|  |  | **Citric acid cycle** | |  | |  |
| PSE_0214 | PJE062_3108 | Aconitate hydratase | | *acnA* | | 4.2.1.3 |
| PSE_0452 | PJE062_3422 | Aconitate hydratase 2 | | *acnB* | | 4.2.1.3 |
| PSE_0603 | PJE062_2553 | Succinate dehydrogenase cytochrome b556 subunit (Cytochrome b-556) | | *sdhC* | | 1.3.99.1 |
| PSE_0604 | PJE062_2930 | Succinate dehydrogenase hydrophobic membrane anchor subunit | | *sdhD* | | 1.3.99.1 |
| PSE_0605 | PJE062_2640 | Succinate dehydrogenase flavoprotein subunit | | *sdhA* | | 1.3.99.1 |
| PSE_0606 | PJE062_2569 | Succinate dehydrogenase iron-sulfur subunit | | *sdhB* | | 1.3.99.1 |
| PSE_0614 | PJE062_3013 | Malate dehydrogenase | | *mdh* | | 1.1.1.37 |
| PSE_0615 | PJE062_2857 | Succinyl-CoA synthetase subunit beta | | *sucC* | | 6.2.1.5 |
| PSE_0616 | ─ | succinyl-CoA synthetase subunit alpha | | *sucD* | | 6.2.1.5 |
| PSE_0617 | PJE062_2799 | alpha-ketoglutarate decarboxylase | | *sucA (odhA)* | | 1.2.4.2 |
| PSE_0618 | PJE062_2606 | Dihydrolipoamide acetyltransferase | | *sucB* | | 2.3.1.61 |
| PSE_2580 | PJE062_4244 | Fumarate hydratase class I | | *fumA* | | 4.2.1.2 |
| PSE_4461 | PJE062_4634 | Fumarate hydratase class II (Fumarase C) | | *fumC* | | 4.2.1.2 |
| PSE_3294 | PJE062_1258 | Dihydrolipoyllysine-residue acetyltransferase component of pyruvate dehydrogenase complex (E2) | | *pdhC* | | 2.3.1.12 |
| PSE_3295 | PJE062_1014 | Pyruvate dehydrogenase E1 component subunit beta | | *pdhB* | | 1.2.4.1 |
| PSE_3296 | PJE062_1075 | Pyruvate dehydrogenase E1 component subunit alpha | | *pdhA* | | 1.2.4.1 |
| PSE_3391 | PJE062_1324 | Citrate synthase | | *cisY* | | 2.3.3.1 |
| PSE_4065 | PJE062_4617 | Isocitrate dehydrogenase | | *icd (idhA)* | | 1.1.1.42 |
| PSE_4461 | PJE062_4634 | Fumarate hydratase class II (Fumarase C) | | *fumC* | | 4.2.1.2 |
| PSE_4866 | PJE062_2056 | Pyruvate carboxylase | | *pyc* | | 6.4.1.1 |
| PSE_4928 | PJE062_2277 | Citrate synthase | | *cisY* | | 2.3.3.1 |
|  |  |  | |  | |  |
|  |  |  | |  | |  |
| **Table S 2.** Continued |  |  | |  | |  |
| **Locus  FO-BEG1** | **Locus  JE062** | **Product** | | **Gene** | | **EC** |
|  |  | **Aromatic compound degradation** | |  | |  |
| PSE_1913 | PJE062_845 | Carboxymuconolactone decarboxylase | | *pcaC* | | 4.1.1.44 |
| PSE_2272 | PJE062_483 | P-hydroxybenzoate hydroxylase (4-hydroxybenzoate 3-monooxygenase) | | *pobA* | | 1.14.13.2 |
| PSE_2278 | PJE062_940 | 3-oxoadipate enol-lactonase | | *pcaD* | | 3.1.1.24 |
| PSE_2279 | PJE062_920 | Protocatechuate 3,4-dioxygenase beta chain | | *pcaH* | | 1.13.11.3 |
| PSE_2280 | PJE062_637 | Protocatechuate 3,4-dioxygenase alpha chain | | *pcaG* | | 1.13.11.3 |
| PSE_2281 | PJE062_798 | 3-oxoadipate:succinyl-CoA transferase, subunit A | | *pcaI* | | 2.8.3.12 |
| PSE_2282 | PJE062_821 | 3-oxoadipate:succinyl-CoA transferase, subunit B | | *pcaJ* | | 2.8.3.- |
| PSE_2283 | PJE062_938 | Beta-ketoadipyl-CoA thiolase | | *pcaF* | | 2.3.1.16 |
| PSE_2284 | PJE062_754 | 3-carboxy-cis,cis-muconate cycloisomerase | | *pcaB* | | 5.5.1.2 |
| PSE_2827 | PJE062_1336 | Benzoate 1,2-dioxygenase subunit beta | | *benB* | | 1.14.12.- |
| PSE_2828 | PJE062_1217 | Benzoate 1,2-dioxygenase subunit alpha | | *benA* | | 1.14.12.- |
| PSE_2829 | PJE062_1491 | Vanillate O-demethylase oxidoreductase | | *vanB* | | 1.14.13.82 |
|  |  |  | |  | |  |
|  |  | **Carbon monoxide oxidation** | |  | |  |
| PSE_1023 | PJE062_4489 | Carbon monoxide dehydrogenase subunit G | | *coxG* | | 1.2.99.2 |
| PSE_1024 | PJE062_4477 | Carbon monoxide dehydrogenase small chain | | *coxS* | | 1.2.99.2 |
| PSE_1025 | PJE062_4485 | Carbon monoxide dehydrogenase large chain | | *coxL* | | 1.2.99.2 |
| PSE_1027 | PJE062_4412 | Carbon monoxide dehydrogenase medium chain | | *coxM* | | 1.2.99.2 |
|  |  |  | |  | |  |
|  |  | **Mixed acid fermentation** | |  | |  |
| PSE_0477 | PJE062_2774 | L-lactate dehydrogenase | | *ldh* | | 1.1.1.27 |
| PSE_1924 | PJE062_844 | D-lactate dehydrogenase | | *dld* | | 1.1.1.28 |
| PSE_3254 | PJE062_1131 | Formate acetyltransferase | | *tdcE* | | 2.3.1.54 |
| PSE_3255 | PJE062_1378 | Pyruvate formate-lyase activating enzyme | | *pflA* | | 1.97.1.4 |
| PSE_1331 | PJE062_1899 | Aldehyde-alcohol dehydrogenase | | *adhE* | | 1.1.1.1 |
| PSE_1086 | PJE062_4486 | Phosphate acetyltransferase (Phosphotransacetylase) | | *pta* | | 2.3.1.8 |
| PSE_1087 | PJE062_4379 | Acetate kinase | | *ackA* | | 2.7.2.1 |
| PSE_0614 | PJE062_3013 | Malate dehydrogenase | | *mdh* | | 1.1.1.37 |
| PSE_2580 | PJE062_4244 | Fumarate hydratase class I | | *fumA* | | 4.2.1.2 |
| PSE_4461 | PJE062_4634 | Fumarate hydratase class II (Fumarase C) | | *fumC* | | 4.2.1.2 |
| PSE_2225 | PJE062_741 | Fumarate reductase/succinate dehydrogenase flavoprotein | |  | |  |
| PSE_4599 | PJE062_2498 | Propionyl-CoA carboxylase, beta subunit | |  | |  |
| PSE_4597 | PJE062_2449 | Propionyl-CoA carboxylase alpha chain | |  | |  |
| PSE_2418 | PJE062_156 | Methylmalonyl-CoA mutase large subunit | | *mutB* | | 5.4.99.2 |
| PSE_2419 | PJE062_248 | Methylmalonyl-CoA mutase small subunit | | *mutA* | | 5.4.99.2 |
| PSE_3421 | PJE062_1661 | Methylmalonyl-CoA epimerase | | *mceE* | | 5.1.99.1 |
| PSE_1247 | PJE062_4342 | succinyl-CoA:3-ketoacid-coenzyme A transferase subunit A | | *scoA* | | 2.8.3.5 |
| PSE_1248 | PJE062_4358 | succinyl-CoA:3-ketoacid-coenzyme A transferase subunit B | | *scoB* | | 2.8.3.5 |
|  |  |  | |  | |  |
|  |  |  | |  | |  |
|  |  |  | |  | |  |
|  |  |  | |  | |  |
| **Table S 2.** Continued |  |  | |  | |  |
| **Locus  FO-BEG1** | **Locus  JE062** | **Product** | | **Gene** | | **EC** |
|  |  | **Denitrification** | |  | |  |
| PSE_0757 | PJE062_2921 | Respiratory nitrate reductase, alpha subunit | | *narG* | | 1.7.99.4 |
| PSE_0758 | PJE062_3029 | Respiratory nitrate reductase beta subunit | | *narH* | | 1.7.99.4 |
| PSE_0759 | PJE062_2637 | Respiratory nitrate reductase, delta subunit | | *narJ* | | 1.7.99.4 |
| PSE_0760 | PJE062_2605 | Respiratory nitrate reductase, gamma subunit | | *narI* | | 1.7.99.4 |
| PSE_0895 | PJE062_2542 | Nitrite reductase protein (NO-forming) | | *nirN* | | 1.7.2.1 |
| PSE_0897 | PJE062_2612 | Denitrification system component NirT | | *nirT* | |  |
| PSE_0898 | PJE062_2813 | Nitrite reductase | | *nirS* | | 1.7.2.1 |
| PSE_2203 | PJE062_731 | Protein NirF | | *nriF* | | 1.7.2.1 |
| PSE_2204 | PJE062_627 | Nitrite reductase heme biosynthesis D/L protein | |  | |  |
| PSE_2205 | PJE062_508 | Protein NirG | | *nirG* | |  |
| PSE_2207 | PJE062_853 | Nitrite reductase | | *nirS* | | 1.7.2.1 |
| PSE_4550 | PJE062_2454 | Protein NorD | | *norD* | | 1.7.99.7 |
| PSE_4551 | PJE062_2491 | Protein NorQ | | *norQ* | | 1.7.99.7 |
| PSE_4552 | PJE062_2475 | Nitric oxide reductase subunit B | | *norB* | | 1.7.99.7 |
| PSE_4553 | PJE062_2486 | Nitric oxide reductase subunit C | | *norC* | | 1.7.99.7 |
| PSE_3127 | PJE062_1200 | Regulatory protein NosR | | *nosR* | |  |
| PSE_3128 | PJE062_1080 | Nitrous-oxide reductase | | *nosZ* | | 1.7.99.6 |
| PSE_3129 | PJE062_1307 | nitrous oxide maturation protein NosD | | *nosD* | |  |
| PSE_3130 | PJE062_965 | Copper transport ATP-binding protein NosF | | *nosF* | |  |
| PSE_3131 | PJE062_1456 | Membrane protein NosY | | *nosY* | |  |
| PSE_3132 | PJE062_1117 | NosL protein required for nitrous oxide reduction | | *nosL* | |  |
| PSE_2100 | PJE062_842 | Cytochrome c-type protein NapC | | *napC* | |  |
| PSE_2101 | PJE062_629 | Nitrate reductase cytochrome c-type subunit (NapB) | | *napB* | |  |
| PSE_2102 | PJE062_585 | Nitrate reductase, large subunit, periplasmic | |  | |  |
| PSE_2103 | PJE062_729 | Protein NapD | | *napD* | |  |
| PSE_2104 | PJE062_910 | Ferredoxin-type protein NapF | | *napF* | |  |
| PSE_2105 | PJE062_562 | Periplasmic nitrate reductase protein | | *napE* | |  |
| PSE_4539 | PJE062_2443 | Cytochrome c-type protein NapC | | *napC* | |  |
| PSE_4540 | PJE062_2471 | Nitrate reductase cytochrome c-type subunit (NapB) | | *napB* | |  |
| PSE_4541 | PJE062_2490 | Ferredoxin-type protein NapH | | *napH* | |  |
| PSE_4542 | PJE062_2479 | MauM/NapG ferredoxin-type protein | | *napG* | |  |
| PSE_4543 | PJE062_2442 | Nitrate reductase, large subunit, periplasmic | |  | |  |
| PSE_4544 | PJE062_2506 | Protein NapD | | *napD* | |  |
|  |  |  | |  | |  |
|  |  | **Oxidation of reduced sulfur compounds** | |  | |  |
| PSE_1359 | PJE062_5277 | Sulfide dehydrogenase [flavocytochrome c] flavoprotein chain | | *soxF* | |  |
| PSE_1360 | PJE062_5324 | Diheme cytochrome c SoxE | | *soxE* | |  |
| PSE_1361 | PJE062_5237 | Diheme cytochrome c SoxD | | *soxD* | |  |
| PSE_1362 | PJE062_5256 | Sulfur oxidation molybdopterin C protein, SoxC | | *soxC* | |  |
| PSE_1363 | PJE062_5219 | Sulfur oxidation B protein | | *soxB* | |  |
| PSE_1364 | PJE062_5333 | Diheme cytochrome c SoxA | | *soxA* | |  |
| PSE_1365 | PJE062_5182 | Sulfur oxidation Z protein | | *soxZ* | |  |
| PSE_1366 | PJE062_5189 | Sulfur oxidation protein SoxY | | *soxY* | |  |
| PSE_1367 | PJE062_5241 | Monoheme cytochrome c SoxX | | *soxX* | |  |
| PSE_1368 | PJE062_5328 | Thioredoxin SoxW | | *soxW* | |  |
|  |  |  | |  | |  |
|  |  |  | |  | |  |
|  |  |  | |  | |  |
| **Table S 2.** Continued |  |  | |  | |  |
| **Locus  FO-BEG1** | **Locus  JE062** | **Product** | | **Gene** | | **EC** |
|  |  | **Phosphonate utilization** | |  | |  |
| PSE_4849 | PJE062_2269 | Phosphonate metabolism PhnG | | *phnG* | |  |
| PSE_4850 | PJE062_2196 | carbon-phosphorus lyase complex subunit | | *phnH* | |  |
| PSE_4851 | PJE062_2313 | Protein PhnI | | *phnI* | |  |
| PSE_4852 | PJE062_2247 | Protein PhnJ | | *phnJ* | |  |
| PSE_4853 | PJE062_2188 | Phosphonates transport ATP-binding protein PhnK | | *phnK* | |  |
| PSE_4854 | PJE062_2041 | Phosphonates transport ATP-binding protein PhnL | | *phnL* | |  |
| PSE_4857 | PJE062_2279 | Protein PhnM | | *phnM* | |  |
| PSE_4858 | PJE062_2090 | ATP-binding protein PhnN | | *phnN* | |  |
| PSE_3627 | PJE062_1982 | Phosphonates transport system permease protein PhnE | | *phnE* | |  |
| PSE_3628 | PJE062_2033 | Phosphonates transport system permease protein PhnE | | *phnE* | |  |
| PSE_3629 | PJE062_2039 | ABC transporter, phosphonate, periplasmic substrate-binding protein PhnD | | *phnD* | |  |
| PSE_3630 | PJE062_2265 | Phosphonates import ATP-binding protein PhnC | | *phnC* | |  |
|  |  |  | |  | |  |
| **Locus  FO-BEG1** | **Locus  JE062** | **Product** | **Gene** | | **Evalue / identity [%] to *Marinomonas* sp. MWYl1** | |
|  |  | **DMSP utilization** |  | |  | |
|  |  | DMSP cleavage |  | |  | |
| PSE_2912 | PJE062_997 | L-carnitine dehydratase/bile acid-inducible  protein F | *dddD* | | 0.0 / 70% | |
| PSE_2914 | PJE062_1320 | transporter, betaine/carnitine/choline transporter (BCCT) family | *dddT* | | 0.0 / 70% | |
| PSE_2915 | PJE062_1654 | Alcohol dehydrogenase | *dddB* | | 0.0 / 70% | |
| PSE_2529 | PJE062_62 | methylmalonate-semialdehyde dehydrogenase | *dddC* | | 0.0 / 56% | |
| PSE_2913 | PJE062_1493 | transcriptional regulator, LysR family protein | *dddR* | | 8.0E-107 / 53% | |
|  |  |  |  | |  | |
|  |  |  |  | | **Evalue / identity [%] to *Rugeria pomeroyi DSS-3*** | |
|  |  | DMSP demethylation |  | |  | |
| PSE_2975 | PJE062_1074 | aminomethyl transferase family protein | *dmdA* | | 5.0E-20 / 24% | |
| PSE_4733 | PJE062_2234 | acyl-CoA synthetase | *dmdB* | | 3.0E-72 / 30% | |
| PSE_1622 | PJE062_240 | L-aspartate oxidase | *dmdC* | | 2.0E-149 / 43% | |
| PSE_3286 | PJE062_1360 | Enoyl-CoA hydratase/isomerase | *dmdD* | | 5.0E-26 / 32% | |

**Table S 3.** Genes detected in the genomes of *Pseudovibrio* sp. FO-BEG1 and JE062 coding for predicted TRAP transporter subunits. Genes that could not be detected in the not closed genome of strain JE062 are indicated with ‘─’.

| **Locus  FO-BEG1** | **Locus  JE062** | **Product** | **Gene** |
| --- | --- | --- | --- |
|  |  | **TRAP Transporter** |  |
| PSE_0923 | PJE062_2908 | TRAP dicarboxylate transporter, DctM subunit | *dctM* |
| PSE_0924 | PJE062_2533 | TRAP dicarboxylate transporter, DctQ subunit | *dctQ* |
| PSE_0925 | PJE062_2627 | TRAP dicarboxylate transporter, DctP subunit | *dctP* |
|  |  |  |  |
| PSE_1209 | PJE062_4388 | TRAP dicarboxylate transporter, DctP subunit | *dctP* |
| PSE_1316 | PJE062_1939 | TRAP dicarboxylate transporter, DctM subunit | *dctM* |
| PSE_1317 | PJE062_1874 | TRAP dicarboxylate transporter, DctQ subunit | *dctQ* |
|  |  |  |  |
| PSE_1426 | PJE062_5273 | TRAP dicarboxylate transporter, DctP subunit | *dctP* |
| PSE_1427 | PJE062_5331 | TRAP dicarboxylate transporter, DctQ subunit | *dctQ* |
| PSE_1428 | PJE062_5225 | TRAP dicarboxylate transporter, DctM subunit | *dctM* |
|  |  |  |  |
| PSE_1834 | ─ | TRAP dicarboxylate transporter, DctM subunit | *dctM* |
| PSE_1835 | ─ | TRAP dicarboxylate transporter, DctQ subunit | *dctQ* |
| PSE_1836 | ─ | TRAP dicarboxylate transporter, DctP subunit | *dctP* |
|  |  |  |  |
| PSE_1888 | PJE062_3892 | TRAP dicarboxylate transporter, DctM subunit | *dctM* |
| PSE_1889 | PJE062_3768 | TRAP dicarboxylate transporter, DctQ subunit | *dctQ* |
| PSE_1890 | PJE062_3743 | TRAP dicarboxylate transporter, DctP subunit | *dctP* |
|  |  |  |  |
| PSE_1919 | PJE062_932 | TRAP dicarboxylate transporter, DctM subunit | *dctM* |
| PSE_1920 | PJE062_501 | TRAP dicarboxylate transporter, DctQ subunit | *dctQ* |
| PSE_1921 | PJE062_925 | TRAP dicarboxylate transporter, DctP subunit | *dctP* |
|  |  |  |  |
| PSE_1925 | PJE062_918 | TRAP dicarboxylate transporter, fused membrane component DctQM | *dctQM* |
| PSE_1926 | PJE062_600 | TRAP dicarboxylate transporter, DctP subunit | *dctP* |
|  |  |  |  |
| PSE_2239 | PJE062_646 | TRAP transporter solute receptor, TAXI family |  |
| PSE_2240 | PJE062_879 | TRAP dicarboxylate transporter, fused membrane component DctQM | *dctQM* |
|  |  |  |  |
| PSE_2274 | PJE062_919 | TRAP dicarboxylate transporter, DctM subunit | *dctM* |
| PSE_2275 | ─ | TRAP dicarboxylate transporter, DctQ subunit | *dctQ* |
| PSE_2276 | PJE062_523 | TRAP dicarboxylate transporter, DctP subunit | *dctP* |
|  |  |  |  |
| PSE_2337 | PJE062_584 | TRAP transporter solute receptor, TAXI family |  |
| PSE_2338 | PJE062_530 | TRAP dicarboxylate transporter, fused membrane component DctQM | *dctQM* |
|  |  |  |  |
| PSE_2346 | PJE062_1811 | TRAP dicarboxylate transporter, DctQ subunit | *dctQ* |
| PSE_2347 | PJE062_1617 | TRAP dicarboxylate transporter, DctM subunit | *dctM* |
| PSE_2348 | PJE062_1686 | TRAP dicarboxylate transporter, DctP subunit | *dctP* |
|  |  |  |  |
| PSE_2801 | PJE062_303 | TRAP dicarboxylate transporter, DctP subunit | *dctP* |
| PSE_2802 | PJE062_463 | TRAP dicarboxylate transporter, DctQ subunit | *dctQ* |
| PSE_2803 | PJE062_103 | TRAP dicarboxylate transporter, DctM subunit | *dctM* |
|  |  |  |  |
| **Table S 3.** Continued |  |  |  |
| **Locus  FO-BEG1** | **Locus  JE062** | **Product** | **Gene** |
| PSE_2830 | PJE062_1070 | TRAP dicarboxylate transporter, DctP subunit | *dctP* |
| PSE_2831 | PJE062_1159 | TRAP dicarboxylate transporter, DctQ subunit | *dctQ* |
| PSE_2832 | PJE062_1444 | TRAP dicarboxylate transporter, DctM subunit | *dctM* |
|  |  |  |  |
| PSE_2907 | PJE062_1331 | TRAP dicarboxylate transporter, fused membrane component DctQM | *dctQM* |
| PSE_2908 | PJE062_1118 | TRAP transporter solute receptor, TAXI family protein |  |
|  |  |  |  |
| PSE_2946 | PJE062_1639 | TRAP dicarboxylate transporter, DctP subunit | *dctP* |
| PSE_2947 | PJE062_1073 | TRAP dicarboxylate transporter, DctQ subunit | *dctQ* |
| PSE_2948 | PJE062_1496 | TRAP dicarboxylate transporter, DctM subunit | *dctM* |
|  |  |  |  |
| PSE_3111 | ─ | TRAP dicarboxylate transporter, DctP subunit | *dctP* |
| PSE_3112 | ─ | TRAP dicarboxylate transporter, DctQ subunit | *dctQ* |
| PSE_3113 | ─ | TRAP dicarboxylate transporter, DctM subunit | *dctM* |
|  |  |  |  |
| PSE_3353 | PJE062_1507 | TRAP dicarboxylate transporter, fused membrane component DctQM | *dctQM* |
| PSE_3354 | PJE062_1151 | TRAP transporter solute receptor, TAXI family protein |  |
|  |  |  |  |
| PSE_3664 | PJE062_1463 | TRAP dicarboxylate transporter, DctP subunit | *dctP* |
| PSE_3665 | PJE062_1642 | TRAP dicarboxylate transporter, DctQ subunit | *dctQ* |
| PSE_3666 | PJE062_1723 | TRAP dicarboxylate transporter, DctM subunit | *dctM* |
|  |  |  |  |
| PSE_3721 | PJE062_1201 | TRAP dicarboxylate transporter, DctM subunit | *dctM* |
| PSE_3722 | PJE062_1120 | TRAP dicarboxylate transporter, DctQ subunit | *dctQ* |
| PSE_3723 | PJE062_1561 | TRAP dicarboxylate transporter, DctP subunit | *dctP* |
|  |  |  |  |
| PSE_3912 | ─ | TRAP dicarboxylate transporter, DctP subunit | *dctP* |
| PSE_3913 | ─ | TRAP dicarboxylate transporter, DctQ subunit | *dctQ* |
| PSE_3914 | ─ | TRAP dicarboxylate transporter, DctM subunit | *dctM* |
|  |  |  |  |
| PSE_4136 | PJE062_4972 | TRAP dicarboxylate transporter- DctP subunit | *dctP* |
|  |  |  |  |
| PSE_4577 | PJE062_2484 | TRAP dicarboxylate transporter, fused membrane component DctQM | *dctQM* |
| PSE_4578 | PJE062_2464 | TRAP transporter solute receptor, TAXI family protein |  |
|  |  |  |  |
| PSE_4810 | PJE062_2025 | TRAP dicarboxylate transporter, DctM subunit | *dctM* |
| PSE_4811 | PJE062_2312 | TRAP dicarboxylate transporter, DctQ subunit | *dctQ* |
| PSE_4812 | PJE062_2046 | TRAP dicarboxylate transporter, DctP subunit | *dctP* |
|  |  |  |  |
| PSE_4845 | PJE062_2117 | TRAP dicarboxylate transporter, DctP subunit | *dctP* |
|  |  |  |  |
| PSE_4929 | PJE062_2193 | TRAP dicarboxylate transporter, DctP subunit | *dctP* |
| PSE_4930 | PJE062_2118 | TRAP dicarboxylate transporter, DctM subunit | *dctM* |
| PSE_4931 | PJE062_2335 | TRAP dicarboxylate transporter, DctQ subunit | *dctQ* |
| PSE_4932 | PJE062_2112 | TRAP dicarboxylate transporter, DctP subunit | *dctP* |
|  |  |  |  |
|  |  |  |  |
| **Table S 3.** Continued |  |  |  |
| **Locus  FO-BEG1** | **Locus  JE062** | **Product** | **Gene** |
| PSE_p0100 | PJE062_587 | TRAP dicarboxylate transporter, DctP subunit | *dctP* |
| PSE_p0101 | PJE062_597 | TRAP dicarboxylate transporter, DctQ subunit | *dctQ* |
| PSE_p0102 | PJE062_692 | TRAP dicarboxylate transporter, DctM subunit | *dctM* |
|  |  |  |  |
| PSE_p0107 | PJE062_891 | TRAP dicarboxylate transporter, DctP subunit | *dctP* |
| PSE_p0108 | PJE062_791 | TRAP dicarboxylate transporter, DctQ subunit | *dctQ* |
| PSE_p0109 | PJE062_739 | TRAP dicarboxylate transporter, DctM subunit | *dctM* |
|  |  |  |  |
| PSE_p0130 | PJE062_726 | TRAP dicarboxylate transporter, DctP subunit | *dctP* |
| PSE_p0131 | PJE062_678 | TRAP dicarboxylate transporter, DctQ subunit | *dctQ* |
| PSE_p0132 | PJE062_526 | TRAP dicarboxylate transporter, DctM subunit | *dctM* |
|  |  |  |  |
| PSE_p0325 | PJE062_3836 | TRAP dicarboxylate transporter, DctQ subunit | *dctQ* |
| PSE_p0326 | PJE062_3646 | TRAP dicarboxylate transporter, DctM subunit | *dctM* |
| PSE_p0327 | PJE062_3830 | TRAP dicarboxylate transporter, DctP subunit | *dctP* |
|  |  |  |  |
| PSE_p0307 | PJE062_3918 | TRAP dicarboxylate transporter, DctP subunit | *dctP* |
|  |  |  |  |
| PSE_p0343 | PJE062_3801 | TRAP dicarboxylate transporter, DctQ subunit | *dctQ* |
| PSE_p0344 | PJE062_3939 | TRAP dicarboxylate transporter, DctM subunit | *dctM* |
| PSE_p0345 | PJE062_3906 | TRAP dicarboxylate transporter, DctP subunit | *dctP* |
|  |  |  |  |
| PSE_p0362 | PJE062_3865 | TRAP dicarboxylate transporter, DctP subunit | *dctP* |
| PSE_p0363 | PJE062_3671 | TRAP dicarboxylate transporter, DctQ subunit | *dctQ* |
| PSE_p0364 | PJE062_3954 | TRAP dicarboxylate transporter, DctM subunit | *dctM* |
|  |  |  |  |
| PSE_p0369 | PJE062_3631 | TRAP dicarboxylate transporter, DctM subunit | *dctM* |
| PSE_p0370 | PJE062_3612 | TRAP dicarboxylate transporter, DctQ subunit | *dctQ* |
| PSE_p0371 | PJE062_3938 | TRAP dicarboxylate transporter, DctP subunit | *dctP* |
|  |  |  |  |
| PSE_p0394 | PJE062_3749 | TRAP dicarboxylate transporter, DctM subunit | *dctM* |
| PSE_p0395 | PJE062_3639 | TRAP dicarboxylate transporter, DctQ subunit | *dctQ* |
| PSE_p0396 | PJE062_3658 | TRAP dicarboxylate transporter, DctP subunit | *dctP* |

**Table S 4.** Genes detected in the genomes of *Pseudovibrio* sp. FO-BEG1 and JE062 coding for predicted ABC transporter subunits. Genes that could not be detected in the not closed genome of strain JE062 are indicated with ‘─’. Pfam model specifies the family, to which the identified protein belongs, according with the Pfam database (Bateman *et al.*, 2004). Absence of a gene name indicates that no assignment was made due to missing of this parameter for the respective genes. Predicted substrate specificity was derived from the annotations of the genes belonging to the respective ABC transporter system.

| **Locus  FO-BEG1** | **Locus  JE062** | **Pfam Model** | **Product** | **Gene** | **Predicted substrate specificity** |
| --- | --- | --- | --- | --- | --- |
|  |  |  | **ABC Transporter** |  |  |
| PSE_0112 | PJE062_3499 | ABC_tran | Glycine betaine/L-proline transport ATP-binding protein ProV | *proV* | **Glycine betaine/  L-proline** |
| PSE_0113 | PJE062_3280 | BPD_transp_1 | Glycine betaine/L-proline transport system permease protein ProW | *proW* |
| PSE_0114 | PJE062_3213 | OpuAC | Glycine betaine-binding protein | *proX* |
|  |  |  |  |  |  |
| PSE_0420 | PJE062_3099 | Peripla_BP_2 | Hemin-binding periplasmic protein HmuT | *hmuT* | **Hemin** |
| PSE_0421 | PJE062_3210 | FecCD | Hemin transport system permease protein HmuU | *hmuU* |
| PSE_0422 | PJE062_3070 | ABC_tran | Hemin import ATP-binding protein HmuV | *hmuV* |
|  |  |  |  |  |  |
| PSE_0430 | PJE062_3307 | OpuAC | Glycine betaine-binding protein | *proX* | **Glycine betaine/  L-proline** |
| PSE_0431 | PJE062_3373 | BPD_transp_1 | Glycine betaine/L-proline transport system permease protein ProW | *proW* |
| PSE_0432 | PJE062_3413 | ABC_tran | Glycine betaine/L-proline transport ATP-binding protein ProV | *proV* |
|  |  |  |  |  |  |
| PSE_0437 | PJE062_3357 | SBP_bac_5 | Periplasmic dipeptide transport protein | *dppA* | **Oligopeptide** |
| PSE_0438 | PJE062_3451 | BPD_transp_1 | Dipeptide transport system permease protein DppB | *dppB* |
| PSE_0439 | PJE062_3076 | BPD_transp_1 | Dipeptide transport system permease protein DppC | *dppC* |
| PSE_0440 | PJE062_3241 | ABC_tran | Dipeptide transport ATP-binding protein DppD | *dppD* |
| PSE_0441 | PJE062_3337 | ABC_tran | Dipeptide transport ATP-binding protein DppF | *dppF* |
|  |  |  |  |  |  |
| PSE_0443 | PJE062_3229 | Peripla_BP_1 | D-ribose-binding protein | *rbsB* | **Sugar** |
| PSE_0444 | PJE062_3285 | ABC_tran | Ribose import ATP-binding protein RbsA | *rbsA* |
| PSE_0445 | PJE062_3140 | BPD_transp_2 | Ribose transport system permease protein RbsC | *rbsC* |
|  |  |  |  |  |  |
|  |  |  |  |  |  |
|  |  |  |  |  |  |
|  |  |  |  |  |  |
|  |  |  |  |  |  |
| **Table S 4.** Continued |  |  |  |  |  |
| **Locus  FO-BEG1** | **Locus  JE062** | **Pfam Model** | **Product** | **Gene** | **Predicted substrate specificity** |
| PSE_0470 | PJE062_2829 | BPD_transp_1 | sn-glycerol-3-phosphate transport system permease protein UgpE | *ugpE* | **Glycerol-3-phosphate** |
| PSE_0471 | PJE062_2766 | BPD_transp_1 | sn-glycerol-3-phosphate transport system permease protein UgpA | *ugpA* |
| PSE_0472 | PJE062_3014 | SBP_bac_1 | sn-glycerol-3-phosphate-binding periplasmic protein UgpB | *ugpB* |
| PSE_0473 | PJE062_2743 | ABC_tran | sn-glycerol-3-phosphate import ATP-binding protein UgpC | *ugpC* |
|  |  |  |  |  |  |
| PSE_0528 | PJE062_2554 | SBP_bac_5 | peptide ABC transporter, periplasmic peptide-binding protein |  | **Oligopeptide** |
| PSE_0529 | PJE062_2658 | SBP_bac_5 | peptide ABC transporter, periplasmic peptide-binding protein |  |
| PSE_0530 | PJE062_2902 | BPD_transp_1 | peptide ABC transporter, permease protein |  |
| PSE_0531 | PJE062_2946 | BPD_transp_1 | peptide ABC transporter permease protein |  |
| PSE_0532 | PJE062_2818 | ABC_tran | peptide ABC transporter, ATP-binding protein |  |
|  |  |  |  |  |  |
| PSE_0587 | PJE062_2984 | FecCD | Hemin transport system permease protein HmuU | *hmuU* | **Hemin** |
| PSE_0588 | PJE062_2677 | Peripla_BP_2 | Hemin-binding periplasmic protein HmuT | *hmuT* |
| PSE_0589 | PJE062_2700 | ABC_tran | Hemin import ATP-binding protein HmuV | *hmuV* |
|  |  |  |  |  |  |
| PSE_0680 | PJE062_2538 | SBP_bac_1 | sn-glycerol-3-phosphate-binding periplasmic protein UgpB | *ugpB* | **Glycerol-3-phosphate** |
| PSE_0681 | PJE062_2885 | BPD_transp_1 | sn-glycerol-3-phosphate transport system permease protein UgpA | *ugpA* |
| PSE_0682 | PJE062_2602 | BPD_transp_1 | sn-glycerol-3-phosphate transport system permease protein UgpE | *ugpE* |
| PSE_0683 | PJE062_2790 | ABC_tran | sn-glycerol-3-phosphate import ATP-binding protein UgpC | *ugpC* |
|  |  |  |  |  |  |
| PSE_0763 | PJE062_2800 | Bmp | ABC transporter, periplasmic binding protein |  | **Sugar** |
| PSE_0765 | PJE062_2960 | ABC_tran | Ribose import ATP-binding protein RbsA | *rbsA* |
| PSE_0766 | PJE062_2751 | BPD_transp_2 | sugar ABC transporter, permease protein |  |
| PSE_0767 | PJE062_2698 | BPD_transp_2 | sugar ABC transporter, permease protein |  |
|  |  |  |  |  |  |
|  |  |  |  |  |  |
|  |  |  |  |  |  |
|  |  |  |  |  |  |
|  |  |  |  |  |  |
|  |  |  |  |  |  |
|  |  |  |  |  |  |
|  |  |  |  |  |  |
| **Table S 4.** Continued |  |  |  |  |  |
| **Locus  FO-BEG1** | **Locus  JE062** | **Pfam Model** | **Product** | **Gene** | **Predicted substrate specificity** |
| PSE_0856 | PJE062_2634 | ABC_tran | High-affinity branched-chain amino acid transport ATP-binding protein LivF (LIV-I protein F) | *livF* | **Amino acid** |
| PSE_0857 | PJE062_2943 | ABC_tran | High-affinity branched-chain amino acid transport ATP-binding protein LivG (LIV-I protein G) | *livG* |
| PSE_0858 | PJE062_2995 | BPD_transp_2 | High-affinity branched-chain amino acid transport system permease protein LivM (LIV-I protein M) | *livM* |
| PSE_0859 | PJE062_2944 | BPD_transp_2 | High-affinity branched-chain amino acid transport system permease protein LivH (LIV-I protein H) | *livH* |
| PSE_0860 | PJE062_2831 | ANF_receptor | branched-chain amino acid ABC transporter, periplasmic substrate-binding protein |  |
|  |  |  |  |  |  |
| PSE_0900 | PJE062_2932 | ABC_tran | Maltose/maltodextrin import ATP-binding protein MalK | *malK* | **Sugar** |
| PSE_0901 | PJE062_2619 | SBP_bac_1 | sugar uptake ABC transporter periplasmic solute-binding protein |  |
| PSE_0902 | PJE062_2590 | BPD_transp_1 | sugar uptake ABC transporter permease protein |  |
| PSE_0903 | PJE062_2733 | BPD_transp_1 | sugar uptake ABC transporter permease protein |  |
|  |  |  |  |  |  |
| PSE_0914 | PJE062_2898 | SBP_bac_1 | sugar ABC transporter, periplasmic sugar-binding protein |  | **Sugar** |
| PSE_0915 | PJE062_2980 | BPD_transp_1 | sugar ABC transporter, permease protein |  |
| PSE_0916 | PJE062_2684 | BPD_transp_1 | sugar ABC transporter, permease protein |  |
| PSE_0918 | PJE062_2573 | ABC_tran | Lactose transport ATP-binding protein LacK | *lacK* |
|  |  |  |  |  |  |
| PSE_0933 | PJE062_2872 | BPD_transp_1 | Taurine transport system permease protein TauC | *tauC* | **Taurine** |
| PSE_0934 | PJE062_2914 | ABC_tran | Taurine import ATP-binding protein TauB | *tauB* |
| PSE_0935 | PJE062_2710 | SBP_bac_3 | Taurine-binding periplasmic protein | *tauA* |
|  |  |  |  |  |  |
| PSE_1036 | PJE062_4410 | SBP_bac_1 | uncharacterized ABC-type transport system, periplasmic component/surface lipoprotein |  | **Sugar** |
| PSE_1037 | PJE062_4371 | ABC_tran | Ribose import ATP-binding protein RbsA | *rbsA* |
| PSE_1038 | PJE062_4507 | BPD_transp_2 | permease protein, ABC-type sugar transporter |  |
| PSE_1039 | PJE062_4366 | BPD_transp_2 | sugar ABC transporter, permease protein |  |
|  |  |  |  |  |  |
|  |  |  |  |  |  |
|  |  |  |  |  |  |
| **Table S 4.** Continued |  |  |  |  |  |
| **Locus  FO-BEG1** | **Locus  JE062** | **Pfam Model** | **Product** | **Gene** | **Predicted substrate specificity** |
| PSE_1120 | PJE062_4425 | ABC_tran | High-affinity branched-chain amino acid transport ATP-binding protein LivG (LIV-I protein G) | *livG* | **Amino acid** |
| PSE_1121 | PJE062_4370 | ABC_tran | High-affinity branched-chain amino acid transport ATP-binding protein LivF (LIV-I protein F) | *livF* |
| PSE_1122 | PJE062_4385 | BPD_transp_2 | High-affinity branched-chain amino acid transport system permease protein LivH (LIV-I protein H) | *livH* |
| PSE_1123 | PJE062_4430 | BPD_transp_2 | High-affinity branched-chain amino acid transport system permease protein LivM (LIV-I protein M) | *livM* |
| PSE_1124 | PJE062_4454 | ANF_receptor | ABC branched amino acid transporter family, periplasmic substrate-binding protein |  |  |
|  |  |  |  |  |  |
| PSE_1141 | PJE062_2757 | TonB_dep_Rec | Ferrichrome-iron receptor | *fhuA* | **Enterobactin** |
| PSE_1142 | PJE062_2912 | Peripla_BP_2 | Ferrienterobactin-binding periplasmic protein | *fepB* |
| PSE_1143 | PJE062_2884 | FecCD | Ferric enterobactin transport system permease protein FepD | *fepD* |
| PSE_1144 | PJE062_3038 | FecCD | Ferric enterobactin transport system permease protein FepG | *fepG* |
| PSE_1145 | PJE062_2878 | ABC_tran | Ferric enterobactin transport ATP-binding protein FepC | *fepC* |
|  |  |  |  |  |  |
| PSE_1213 | PJE062_4405 | BPD_transp_2 | High-affinity branched-chain amino acid transport system permease protein LivM (LIV-I protein M) | *livM* | **Amino acid** |
| PSE_1214 | PJE062_4382 | BPD_transp_2 | High-affinity branched-chain amino acid transport system permease protein LivH (LIV-I protein H) | *livH* |
| PSE_1215 | PJE062_4456 | ABC_tran | High-affinity branched-chain amino acid transport ATP-binding protein LivF (LIV-I protein F) | *livF* |
| PSE_1216 | PJE062_4457 | ABC_tran | High-affinity branched-chain amino acid transport ATP-binding protein LivG (LIV-I protein G) | *livG* |
| PSE_1217 | PJE062_4428 | SBF | P3 protein (Solute carrier family 10 member 3) |  |
|  |  |  |  |  |  |
| PSE_1243 | PJE062_4352 | SBP_bac_1 | Putrescine-binding periplasmic protein | *potF* | **Putrescine/ Spermidine** |
| PSE_1244 | PJE062_4362 | ABC_tran | Putrescine transport ATP-binding protein PotG | *potG* |
| PSE_1245 | PJE062_4348 | BPD_transp_1 | Putrescine transport system permease protein PotH | *potH* |
| PSE_1246 | PJE062_4343 | BPD_transp_1 | Putrescine transport system permease protein PotI | *potI* |
|  |  |  |  |  |  |
|  |  |  |  |  |  |
|  |  |  |  |  |  |
| **Table S 4.** Continued |  |  |  |  |  |
| **Locus  FO-BEG1** | **Locus  JE062** | **Pfam Model** | **Product** | **Gene** | **Predicted substrate specificity** |
| PSE_1292 | PJE062_1965 | ABC_tran | Lactose transport ATP-binding protein LacK | *lacK* | **Sugar** |
| PSE_1294 | PJE062_1894 | SBP_bac_1 | ABC transporter, substrate-binding protein |  |
| PSE_1296 | PJE062_1958 | BPD_transp_1 | sugar ABC transporter, permease protein, |  |
| PSE_1297 | PJE062_1975 | BPD_transp_1 | sugar ABC transporter, permease protein |  |
|  |  |  |  |  |  |
| PSE_1322 | PJE062_1888 | ABC_tran | Zinc import ATP-binding protein ZnuC | *znuC* | **Zinc** |
| PSE_1323 | PJE062_1960 | ABC-3 | High-affinity zinc uptake system membrane protein ZnuB | *znuB* |
| PSE_1324 | ─ | FUR | Zinc uptake regulation protein (Zinc uptake regulator) | *zur* |
| PSE_2536 | PJE062_14 | SBP_bac_9 | High-affinity zinc uptake system protein ZnuA | *znuA* |
|  |  |  |  |  |  |
| PSE_1389 | PJE062_5224 | BPD_transp_1 | sugar uptake ABC transporter permease protein |  | **Sugar** |
| PSE_1390 | PJE062_5312 | BPD_transp_1 | sugar uptake ABC transporter permease protein |  |
| PSE_1391 | PJE062_5176 | SBP_bac_1 | extracellular solute-binding protein family 1 |  |
| PSE_1392 | PJE062_5319 | ABC_tran | Maltose/maltodextrin import ATP-binding protein MalK | *malK* |
|  |  |  |  |  |  |
| PSE_1675 | PJE062_116 | SBP_bac_9 | Manganese-binding lipoprotein MntA / periplasmic zinc-binding protein TroA | *mntA/troA* | **Manganese** |
| PSE_1676 | PJE062_387 | ABC_tran | Manganese transport system ATP-binding protein MntB / zinc transport system ATP-binding protein TroB | *mntB/troB* |
| PSE_1677 | PJE062_164 | ABC-3 | Manganese transport system membrane protein MntC / zinc transport system membrane protein TroC | *mntC/troC* |
| PSE_1678 | PJE062_301 | ABC-3 | Manganese transport system membrane protein MntD / zinc transport system membrane protein TroD | *mntD/troD* |
|  |  |  |  |  |  |
| PSE_1679 | PJE062_321 | SBP_bac_1 | Spermidine/putrescine-binding periplasmic protein 2 | *potD* | **Putrescine/ Spermidine** |
| PSE_1681 | PJE062_41 | ABC_tran | Spermidine/putrescine import ATP-binding protein PotA | *potA* |
| PSE_1682 | PJE062_317 | BPD_transp_1 | Putrescine transport system permease protein PotH | *potH* |
| PSE_1683 | PJE062_261 | BPD_transp_1 | Spermidine/putrescine transport system permease protein PotC | *potC* |
|  |  |  |  |  |  |
|  |  |  |  |  |  |
|  |  |  |  |  |  |
|  |  |  |  |  |  |
|  |  |  |  |  |  |
| **Table S 4.** Continued |  |  |  |  |  |
| **Locus  FO-BEG1** | **Locus  JE062** | **Pfam Model** | **Product** | **Gene** | **Predicted substrate specificity** |
| PSE_1688 | PJE062_408 | SBP_bac_1 | phosphate ABC transporter, periplasmic binding protein | *pstS* | **Phosphate** |
| PSE_1689 | PJE062_354 | BPD_transp_1 | Phosphate transport system permease protein PstC | *pstC* |
| PSE_1690 | PJE062_413 | BPD_transp_1 | Phosphate transport system permease protein PstA | *pstA* |
| PSE_1691 | PJE062_39 | ABC_tran | Phosphate import ATP-binding protein PstB 1 | *pstB* |
| PSE_1692 | PJE062_279 | PhoU | Phosphate transport system protein PhoU | *phoU* |
|  |  |  |  |  |  |
| PSE_1815 | PJE062_176 | Peripla_BP_1 | periplasmic substrate-binding protein, ABC-type sugar transporter |  | **Sugar** |
| PSE_1816 | PJE062_31 | BPD_transp_2 | sugar ABC transporter, permease protein |  |
| PSE_1817 | PJE062_162 | ABC_tran | sugar ABC transporter, ATP-binding protein |  |
|  |  |  |  |  |  |
| PSE_1875 | PJE062_2861 | SBP_bac_1 | ABC sugar transporter extracellular solute-binding protein, family 1 |  | **Sugar** |
| PSE_1876 | PJE062_2615 | BPD_transp_1 | ABC-type sugar transport system, permease component |  |
| PSE_1877 | PJE062_3016 | BPD_transp_1 | ABC-type sugar transport system, permease component |  |
| PSE_1878 | PJE062_2608 | ABC_tran | ABC-type sugar transport system, ATPase component |  |
|  |  |  |  |  |  |
| PSE_1900 | PJE062_4447 | SBP_bac_1 | Thiamine-binding periplasmic protein | *thiB* | **Thiamine** |
| PSE_1901 | PJE062_4491 | BPD_transp_1 | Thiamine transport system permease protein ThiP | *thiP* |
| PSE_1902 | PJE062_4514 | ABC_tran | Thiamine import ATP-binding protein ThiQ | *thiQ* |
|  |  |  |  |  |  |
| PSE_1931 | PJE062_859 | NMT1 | ABC-type sulfonate transport system periplasmic component |  | **Sulfonates** |
| PSE_1932 | PJE062_632 | BPD_transp_1 | Aliphatic sulfonates transport permease protein SsuC | *ssuC* |
| PSE_1933 | PJE062_685 | ABC_tran | Aliphatic sulfonates import ATP-binding protein SsuB | *ssuB* |
|  |  |  |  |  |  |
| PSE_1938 | PJE062_519 | SBP_bac_1 | iron(III) ABC transporter, substrate-binding protein |  | **Iron** |
| PSE_1939 | PJE062_888 | BPD_transp_1 | iron(III) transport system permease protein FbpB | *fbpB* |
| PSE_1940 | PJE062_677 | ABC_tran | Fe(3+) ions import ATP-binding protein FbpC | *fbpC* |
|  |  |  |  |  |  |
|  |  |  |  |  |  |
|  |  |  |  |  |  |
|  |  |  |  |  |  |
|  |  |  |  |  |  |
| **Table S 4.** Continued |  |  |  |  |  |
| **Locus  FO-BEG1** | **Locus  JE062** | **Pfam Model** | **Product** | **Gene** | **Predicted substrate specificity** |
| PSE_1957 | PJE062_572 | SBP_bac_1 | Sugar-binding periplasmic protein |  | **Sugar** |
| PSE_1958 | PJE062_541 | BPD_transp_1 | ABC-type sugar transport systems, permease components |  |
| PSE_1959 | PJE062_553 | BPD_transp_1 | ABC sugar transporter inner membrane binding protein |  |
| PSE_1960 | PJE062_599 | ABC_tran | Sugar ABC transporter, ATP-binding protein |  |
|  |  |  |  |  |  |
| PSE_1987 | PJE062_601 | ABC_tran | Oligopeptide transport ATP-binding protein OppF | *oppF* | **Oligopeptide** |
| PSE_1988 | PJE062_645 | ABC_tran | Oligopeptide transport ATP-binding protein OppD | *oppD* |
| PSE_1989 | PJE062_931 | BPD_transp_1 | Oligopeptide transport system permease protein OppC | *oppC* |
| PSE_1990 | PJE062_886 | BPD_transp_1 | oligopeptide transport system permease protein OppB | *oppB* |
| PSE_1991 | PJE062_540 | SBP_bac_5 | Periplasmic oligopeptide-binding protein | *oppA* |
|  |  |  |  |  |  |
| PSE_2045 | PJE062_684 | SBP_bac_5 | oligopeptide ABC transporter, periplasmic oligopeptide-binding protein |  | **Oligopeptide** |
| PSE_2048 | PJE062_902 | ABC_tran | Oligopeptide transport ATP-binding protein OppF | *oppF* |
| PSE_2049 | PJE062_662 | ABC_tran | Oligopeptide transport ATP-binding protein OppD | *oppD* |
| PSE_2050 | PJE062_554 | BPD_transp_1 | Glutathione transport system permease protein GsiD | *gsiD* |
| PSE_2051 | PJE062_901 | BPD_transp_1 | Glutathione transport system permease protein GsiC | *gsiC* |
| PSE_2052 | PJE062_642 | SBP_bac_5 | Glutathione-binding protein GsiB | *gsiB* |
|  |  |  |  |  |  |
| PSE_2195 | PJE062_510 | SBP_bac_3 | extracellular solute-binding protein family 3 |  | **Amino acid** |
| PSE_2196 | PJE062_602 | BPD_transp_1 | Inner membrane amino-acid ABC transporter permease protein YecS | |
| PSE_2532 | PJE062_402 | ABC_tran | Cysteine/glutathione ABC transporter membrane/ATP-binding component |  |
|  |  |  |  |  |  |
| PSE_2229 | PJE062_837 | ABC_tran | ABC sugar transporter, ATPase subunit |  | **Sugar** |
| PSE_2230 | PJE062_711 | BPD_transp_2 | ABC sugar transporter, inner membrane subunit |  |
| PSE_2231 | PJE062_898 | Peripla_BP_1 | ABC sugar transporter, periplasmic ligand binding protein |  |
|  |  |  |  |  |  |
| PSE_2242 | PJE062_557 | ABC_tran | Fe(3+) ions import ATP-binding protein FbpC | *fbpC* | **Iron** |
| PSE_2243 | PJE062_594 | BPD_transp_1 | iron(III) transport system permease protein FbpB | *fbpB* |
| PSE_2244 | PJE062_592 | SBP_bac_1 | Iron(III) binding periplasmic protein | *fpbA* |
|  |  |  |  |  |  |
|  |  |  |  |  |  |
| **Table S 4.** Continued |  |  |  |  |  |
| **Locus  FO-BEG1** | **Locus  JE062** | **Pfam Model** | **Product** | **Gene** | **Predicted substrate specificity** |
| PSE_2252 | PJE062_905 | SBP_bac_5 | Oligopeptide-binding protein AppA | *appA* | **Oligopeptide** |
| PSE_2253 | PJE062_503 | BPD_transp_1 | Oligopeptide transport system permease protein OppB | *oppB* |
| PSE_2254 | PJE062_809 | BPD_transp_1 | Oligopeptide transport system permease protein OppC | *oppC* |
| PSE_2255 | PJE062_799 | ABC_tran | Glutathione import ATP-binding protein GsiA | *gsiA* |
|  |  |  |  |  |  |
| PSE_2267 | PJE062_899 | SBP_bac_3 | Cystine-binding periplasmic protein |  | **Amino acid** |
| PSE_2268 | PJE062_509 | BPD_transp_1 | polar amino acid ABC transporter, inner membrane subunit |  |
| PSE_2531 | ─ | ABC_tran | Cysteine/glutathione ABC transporter membrane/ATP-binding component |  |
|  |  |  |  |  |  |
| PSE_2358 | PJE062_12 | ABC_tran | Ferrichrome transport ATP-binding protein FhuC | *fhuC* | **Ferrichrome** |
| PSE_2359 | PJE062_245 | FecCD | Ferrichrome transport system permease protein FhuG | *fhuG* |
| PSE_2360 | PJE062_4 | FecCD | Ferrichrome transport system permease protein FhuB | *fhuB* |
| PSE_2361 | PJE062_378 | Peripla_BP_2 | Iron(III) dicitrate-binding periplasmic protein | *fecB* |
|  |  |  |  |  |  |
| PSE_2389 | PJE062_334 | ABC_tran | ABC transporter, ATP-binding protein |  | **Oligopeptide** |
| PSE_2390 | PJE062_243 | SBP_bac_5 | Periplasmic oligopeptide-binding protein | *oppA* |
| PSE_2391 | PJE062_43 | BPD_transp_1 | Oligopeptide transport system permease protein OppB | *oppB* |
| PSE_2392 | ─ | BPD_transp_1 | Oligopeptide transport system permease protein OppC | *oppC* |
| PSE_2393 | PJE062_72 | ABC_tran | Glutathione import ATP-binding protein GsiA | *gsiA* |
|  |  |  |  |  |  |
| PSE_2493 | PJE062_418 | ABC_tran | Glutathione import ATP-binding protein GsiA | *gsiA* | **Oligopeptide** |
| PSE_2494 | PJE062_435 | SBP_bac_5 | Periplasmic oligopeptide-binding protein | *oppA* |
| PSE_2495 | PJE062_318 | BPD_transp_1 | Oligopeptide transport system permease protein AppB | *appB* |
| PSE_2496 | PJE062_225 | BPD_transp_1 | Oligopeptide transport system permease protein AppC | *appC* |
|  |  |  |  |  |  |
| PSE_2513 | PJE062_36 | BPD_transp_1 | aliphatic sulfonates transport permease protein SsuC | *ssuC* | **Sulfonates** |
| PSE_2514 | PJE062_24 | ABC_tran | Aliphatic sulfonates import ATP-binding protein SsuB | *ssuB* |
| PSE_2515 | PJE062_383 | SBP_bac_1 | Putative aliphatic sulfonates-binding protein | *ssuA* |
|  |  |  |  |  |  |
|  |  |  |  |  |  |
|  |  |  |  |  |  |
|  |  |  |  |  |  |
| **Table S 4.** Continued |  |  |  |  |  |
| **Locus  FO-BEG1** | **Locus  JE062** | **Pfam Model** | **Product** | **Gene** | **Predicted substrate specificity** |
| PSE_2520 | PJE062_357 | ABC_tran | Glutathione import ATP-binding protein GsiA | *gsiA* | **Oligopeptide** |
| PSE_2521 | PJE062_204 | BPD_transp_1 | Glutathione transport system permease protein GsiD | *gsiD* |
| PSE_2522 | PJE062_434 | BPD_transp_1 | Glutathione transport system permease protein GsiC | *gsiC* |
| PSE_2523 | PJE062_42 | SBP_bac_5 | Glutathione-binding protein GsiB | *gsiB* |
|  |  |  |  |  |  |
| PSE_2141 | PJE062_513 | ABC_tran | ATP-binding component of ABC transporter |  | **Nopaline** |
| PSE_2564 | PJE062_4100 | BPD_transp_1 | Nopaline transport system permease protein NocM | *nocM* |
| PSE_2565 | PJE062_4285 | BPD_transp_1 | Nopaline transport system permease protein NocQ | *nocQ* |
| PSE_2566 | PJE062_4170 | SBP_bac_3 | Nopaline-binding periplasmic protein |  |
| PSE_2567 | PJE062_4189 | SBP_bac_3 | Nopaline-binding periplasmic protein |  |
| PSE_2568 | PJE062_4253 | SBP_bac_3 | Nopaline-binding periplasmic protein |  |
|  |  |  |  |  |  |
| PSE_2638 | PJE062_4138 | SBP_bac_5 | periplasmic dipeptide binding protein | *dppA* | **Oligopeptide** |
| PSE_2639 | PJE062_4284 | BPD_transp_1 | Dipeptide transport system permease protein DppB | *dppB* |
| PSE_2640 | PJE062_4303 | BPD_transp_1 | Dipeptide transport system permease protein DppC | *dppC* |
| PSE_2641 | PJE062_4336 | ABC_tran | Dipeptide transport ATP-binding protein DppD | *dppD* |
| PSE_2642 | PJE062_4101 | ABC_tran | Dipeptide transport ATP-binding protein DppF | *dppF* |
| PSE_2643 | PJE062_4114 | SBP_bac_5 | periplasmic dipeptide transport protein | *dppA* |
|  |  |  |  |  |  |
| PSE_2783 | PJE062_1514 | Peripla_BP_2 | periplasmic binding protein |  | **Hemin** |
| PSE_2784 | PJE062_1111 | FecCD | Hemin transport system permease protein HmuU | *hmuU* |
| PSE_2785 | PJE062_1709 | ABC_tran | Hemin import ATP-binding protein HmuV | *hmuV* |
|  |  |  |  |  |  |
| PSE_2808 | PJE062_1377 | SBP_bac_5 | Periplasmic alpha-galactoside-binding protein |  | **Oligopeptide** |
| PSE_2809 | PJE062_1731 | BPD_transp_1 | Oligopeptide transport system permease protein AppB | *appB* |
| PSE_2810 | PJE062_1776 | BPD_transp_1 | Oligopeptide transport system permease protein AppC | *appC* |
| PSE_2811 | PJE062_1552 | ABC_tran | Oligopeptide transport ATP-binding protein AppD | *appD* |
|  |  |  |  |  |  |
|  |  |  |  |  |  |
|  |  |  |  |  |  |
|  |  |  |  |  |  |
|  |  |  |  |  |  |
| **Table S 4.** Continued |  |  |  |  |  |
| **Locus  FO-BEG1** | **Locus  JE062** | **Pfam Model** | **Product** | **Gene** | **Predicted substrate specificity** |
| PSE_2816 | PJE062_1140 | SBP_bac_1 | sugar ABC transporter, periplasmic sugar-binding protein |  | **Sugar** |
| PSE_2817 | PJE062_1562 | BPD_transp_1 | sugar ABC transporter, permease protein |  |
| PSE_2818 | PJE062_1154 | BPD_transp_1 | sugar ABC transporter, permease protein |  |
| PSE_2819 | PJE062_1225 | ABC_tran | sugar ABC transporter, ATP-binding protein |  |
|  |  |  |  |  |  |
| PSE_2868 | PJE062_1053 | SBP_bac_5 | Oligopeptide-binding protein appA | *appA* | **Oligopeptide** |
| PSE_2869 | PJE062_1539 | BPD_transp_1 | Oligopeptide transport system permease protein AppB | *appB* |
| PSE_2870 | PJE062_1757 | BPD_transp_1 | Oligopeptide transport system permease protein AppC | *appC* |
| PSE_2871 | PJE062_1524 | ABC_tran | Oligopeptide transport ATP-binding protein AppD | *appD* |
| PSE_2872 | PJE062_1035 | ABC_tran | Oligopeptide transport ATP-binding protein AppF | *appF* |
|  |  |  |  |  |  |
| PSE_2879 | PJE062_1337 | RbsD_FucU | High affinity ribose transport protein RbsD | *rbsD* | **Sugar** |
| PSE_2880 | PJE062_1777 | ABC_tran | Ribose import ATP-binding protein RbsA | *rbsA* |
| PSE_2881 | PJE062_1423 | BPD_transp_2 | Ribose transport system permease protein RbsC | *rbsC* |
| PSE_2882 | PJE062_1180 | Peripla_BP_1 | D-ribose-binding periplasmic protein | *rbsB* |
|  |  |  |  |  |  |
| PSE_2918 | PJE062_1472 | SBP_bac_1 | extracellular solute-binding protein, family 1 |  | **Sugar** |
| PSE_2919 | PJE062_1003 | BPD_transp_1 | Maltose transport system permease protein MalF | *malF* |
| PSE_2920 | PJE062_1476 | BPD_transp_1 | Maltodextrin transport system permease protein MalD | *malD* |
| PSE_2921 | PJE062_1533 | ABC_tran | Maltose/maltodextrin import ATP-binding protein MalK | *malK* |
|  |  |  |  |  |  |
| PSE_2963 | PJE062_1648 | ANF_receptor | ABC transporter, urea, substrate-binding, UrtA | *urtA* | **Urea** |
| PSE_2964 | PJE062_1405 | BPD_transp_2 | urea ABC transporter, permease protein UrtB | *urtB* |
| PSE_2965 | PJE062_1553 | BPD_transp_2 | urea ABC transporter, permease protein UrtC | *urtC* |
| PSE_2966 | PJE062_1288 | ABC_tran | urea ABC transporter, ATP-binding protein UrtD | *urtD* |
| PSE_2967 | PJE062_1589 | ABC_tran | urea ABC transporter, ATP-binding protein UrtE | *urtE* |
|  |  |  |  |  |  |
| PSE_2977 | PJE062_1645 | BPD_transp_1 | Glycine betaine/L-proline transport system permease protein ProW | *proW* | **Glycine betaine/  L-proline** |
| PSE_2978 | PJE062_1063 | ABC_tran | Glycine betaine/L-proline transport ATP-binding protein ProV | *proV* |
| PSE_2979 | PJE062_1143 | OpuAC | Substrate-binding region of ABC-type glycine betaine transport system |  |
|  |  |  |  |  |  |
| **Table S 4.** Continued |  |  |  |  |  |
| **Locus  FO-BEG1** | **Locus  JE062** | **Pfam Model** | **Product** | **Gene** | **Predicted substrate specificity** |
| PSE_2987 | PJE062_1332 | OpuAC | Choline-binding protein | *opuBC* | **Glycine betaine/ carnitine/choline** |
| PSE_2988 | ─ | BPD_transp_1 | Glycine betaine/carnitine/choline transport system permease protein OpuCD | *opuCD* |
| PSE_2989 | PJE062_1578 | ABC_tran | Glycine betaine/carnitine/choline transport ATP-binding protein OpuCA | *opuCA* |
| PSE_2990 | PJE062_1498 | BPD_transp_1 | Glycine betaine/carnitine/choline transport system permease protein OpuCB | *opuCB* |
|  |  |  |  |  |  |
| PSE_3097 | PJE062_1174 | Peripla_BP_2 | Ferric anguibactin-binding protein | *fatB* | **Ferric anguibactin** |
| PSE_3098 | PJE062_1095 | FecCD | Ferric anguibactin transport system permease protein FatD | *fatD* |
| PSE_3099 | PJE062_1044 | FecCD | Ferric anguibactin transport system permease protein FatC | *fatC* |
| PSE_3100 | PJE062_1329 | ABC_tran | Ferrichrome transport ATP-binding protein FhuC | *fhuC* |
|  |  |  |  |  |  |
| PSE_3153 | PJE062_1631 | ANF_receptor | Leu/Ile/Val-binding protein homolog | *livB* | **Amino acid** |
| PSE_3154 | PJE062_1741 | BPD_transp_2 | High-affinity branched-chain amino acid transport system permease protein LivH (LIV-I protein H) | *livH* |
| PSE_3155 | PJE062_1386 | BPD_transp_2 | High-affinity branched-chain amino acid transport system permease protein LivM (LIV-I protein M) | *livM* |
| PSE_3156 | PJE062_1557 | ABC_tran | High-affinity branched-chain amino acid transport ATP-binding protein LivG (LIV-I protein G) | *livG* |
| PSE_3157 | PJE062_1394 | ABC_tran | High-affinity branched-chain amino acid transport ATP-binding protein LivF (LIV-I protein F) | *livF* |
|  |  |  |  |  |  |
| PSE_3176 | PJE062_1668 | SBP_bac_1 | ABC transporter periplasmic binding protein |  | **Sugar** |
| PSE_3177 | PJE062_1420 | BPD_transp_2 | sugar ABC transporter, permease protein |  |
| PSE_3178 | PJE062_1239 | BPD_transp_2 | sugar ABC transporter, permease protein |  |
| PSE_3179 | PJE062_1523 | ABC_tran | Ribose import ATP-binding protein RbsA | *rbsA* |
|  |  |  |  |  |  |
|  |  |  |  |  |  |
|  |  |  |  |  |  |
|  |  |  |  |  |  |
|  |  |  |  |  |  |
|  |  |  |  |  |  |
| **Table S 4.** Continued |  |  |  |  |  |
| **Locus  FO-BEG1** | **Locus  JE062** | **Pfam Model** | **Product** | **Gene** | **Predicted substrate specificity** |
| PSE_3515 | PJE062_1371 | ABC_tran | Glutamate/glutamine/aspartate/asparagine transport ATP-binding protein BztD | *bztD* | **Amino acid** |
| PSE_3516 | PJE062_1361 | BPD_transp_1 | Glutamate/glutamine/aspartate/asparagine transport system permease protein BztC | *bztC* |
| PSE_3517 | ─ | BPD_transp_1 | Glutamate/glutamine/aspartate/asparagine transport system permease protein BztB | *bztB* |
| PSE_3518 | ─ | SBP_bac_3 | Glutamate/glutamine/aspartate/asparagine-binding protein BztA | *bztA* |
|  |  |  |  |  |  |
| PSE_3627 | PJE062_1374 | BPD_transp_1 | Phosphonates transport system permease protein PhnE | *phnE* | **Phosphonate** |
| PSE_3628 | PJE062_1147 | BPD_transp_1 | Phosphonates transport ATP-binding protein PhnL | *phnL* |
| PSE_3629 | PJE062_1356 | SBP_bac_3 | ABC transporter, phosphonate, periplasmic substrate-binding protein PhnD | *phnD* |
| PSE_3630 | PJE062_1025 | ABC_tran | Phosphonates import ATP-binding protein PhnC | *phnC* |
|  |  |  |  |  |  |
| PSE_3640 | PJE062_1291 | SBP_bac_1 | ABC transporter, substrate binding protein (sugar) |  | **Sugar** |
| PSE_3641 | PJE062_983 | BPD_transp_1 | ABC transporter, membrane spanning protein (sugar) |  |
| PSE_3642 | PJE062_1417 | BPD_transp_1 | ABC transporter, membrane spanning protein (sugar) |  |
| PSE_3644 | PJE062_1309 | ABC_tran | ABC transporter, nucleotide binding/ATPase protein (sugar) |  |
|  |  |  |  |  |  |
| PSE_3646 | PJE062_1305 | Peripla_BP_1 | periplasmic binding protein/LacI transcriptional regulator |  | **Amino acid** |
| PSE_3647 | PJE062_1083 | SBP_bac_3 | putative amino acid uptake ABC transporter periplasmic solute-binding protein |  |
| PSE_3648 | PJE062_1302 | BPD_transp_1 | Amino acid uptake ABC transporter permease protein |  |
|  |  |  |  |  |  |
| PSE_3653 | PJE062_1142 | SBP_bac_1 | Putrescine-binding periplasmic protein | *potF* | **Putrescine/ Spermidine** |
| PSE_3654 | PJE062_1571 | ABC_tran | Putrescine transport ATP-binding protein PotG | *potG* |
| PSE_3655 | PJE062_1526 | BPD_transp_1 | Putrescine transport system permease protein PotH | *potH* |
| PSE_3656 | PJE062_1564 | BPD_transp_1 | Putrescine transport system permease protein PotI | *potI* |
|  |  |  |  |  |  |
|  |  |  |  |  |  |
|  |  |  |  |  |  |
|  |  |  |  |  |  |
|  |  |  |  |  |  |
| **Table S 4.** Continued |  |  |  |  |  |
| **Locus  FO-BEG1** | **Locus  JE062** | **Pfam Model** | **Product** | **Gene** | **Predicted substrate specificity** |
| PSE_3671 | PJE062_1069 | ABC_tran | High-affinity branched-chain amino acid transport ATP-binding protein LivF (LIV-I protein F) | *livF* | **Amino acid** |
| PSE_3672 | PJE062_1719 | ABC_tran | High-affinity branched-chain amino acid transport ATP-binding protein LivG (LIV-I protein G) | *livG* |
| PSE_3673 | PJE062_1499 | BPD_transp_2 | High-affinity branched-chain amino acid transport system permease protein LivM (LIV-I protein M) | *livM* |
| PSE_3674 | PJE062_1484 | BPD_transp_2 | High-affinity branched-chain amino acid transport system permease protein LivH (LIV-I protein H) | *livH* |
| PSE_3675 | PJE062_1325 | ANF_receptor | Leu/Ile/Val-binding protein homolog 4 | *livB* |
|  |  |  |  |  |  |
| PSE_3919 | ─ | BPD_transp_1 | Maltose transport system permease protein MalG | *malG* | **Sugar** |
| PSE_3920 | ─ | BPD_transp_1 | Maltose transport system permease protein MalF | *malF* |
| PSE_3921 | ─ | SBP_bac_1 | Maltose-binding periplasmic protein | *malE* |
| PSE_3924 | ─ | ABC_tran | Maltose/maltodextrin import ATP-binding protein MalK | *malK* |
|  |  |  |  |  |  |
| PSE_3948 | PJE062_4678 | SBP_bac_1 | extracellular solute-binding protein, family 1 |  | **Sugar** |
| PSE_3950 | ─ | BPD_transp_1 | Multiple sugar-binding transport system permease protein MsmF | *msmF* |
| PSE_3951 | PJE062_5134 | BPD_transp_1 | L-arabinose transport system permease protein AraQ | *araQ* |
| PSE_3953 | PJE062_4603 | ABC_tran | Alpha-glucoside transport ATP-binding protein AglK | *aglK* |
|  |  |  |  |  |  |
| PSE_4021 | PJE062_4745 | ANF_receptor | Leu/Ile/Val-binding protein homolog | *livB* | **Amino acid** |
| PSE_4023 | PJE062_44581 | ABC_tran | High-affinity branched-chain amino acid transport ATP-binding protein LivF (LIV-I protein F) | *livF* |
| PSE_4024 | PJE062_4641 | ABC_tran | High-affinity branched-chain amino acid transport ATP-binding protein LivG (LIV-I protein G) | *livG* |
| PSE_4025 | PJE062_4545 | BPD_transp_2 | High-affinity branched-chain amino acid transport system permease protein LivM (LIV-I protein M) | *livM* |
| PSE_4026 | PJE062_5154 | BPD_transp_2 | High-affinity branched-chain amino acid transport system permease protein LivH (LIV-I protein H) | *livH* |
|  |  |  |  |  |  |
|  |  |  |  |  |  |
|  |  |  |  |  |  |
|  |  |  |  |  |  |
|  |  |  |  |  |  |
| **Table S 4.** Continued |  |  |  |  |  |
| **Locus  FO-BEG1** | **Locus  JE062** | **Pfam Model** | **Product** | **Gene** | **Predicted substrate specificity** |
| PSE_4503 | PJE062_4774 | BPD_transp_1 | ABC transporter permease protein |  | **Putrescine/ Spermidine** |
| PSE_4504 | PJE062_4712 | BPD_transp_1 | spermidine/putrescine ABC transporter membrane component |  |
| PSE_4505 | PJE062_5011 | SBP_bac_1 | extracellular solute-binding protein family 1 |  |
| PSE_4506 | PJE062_4906 | ABC_tran | putrescine/spermidine ABC transporter ATPase |  |
|  |  |  |  |  |  |
| PSE_4557 | PJE062_4521 | ABC_tran | Dipeptide transport ATP-binding protein DppF | *dppF* | **Oligopeptide** |
| PSE_4558 | PJE062_2508 | ABC_tran | Dipeptide transport ATP-binding protein DppD | *dppD* |
| PSE_4559 | PJE062_2469 | BPD_transp_1 | Dipeptide transport system permease protein DppC | *dppC* |
| PSE_4560 | PJE062_2453 | BPD_transp_1 | Dipeptide transport system permease protein DppB | *dppB* |
| PSE_4561 | PJE062_2480 | SBP_bac_5 | Periplasmic dipeptide transport protein | *dppA* |
|  |  |  |  |  |  |
| PSE_4562 | PJE062_2473 | ABC_tran | Maltose/maltodextrin import ATP-binding protein MalK | *malK* | **Sugar** |
| PSE_4564 | PJE062_2457 | BPD_transp_1 | Maltose transport system permease protein MalG | *malG* |
| PSE_4565 | PJE062_2513 | BPD_transp_1 | Maltose transport system permease protein MalF | *malF* |
| PSE_4566 | PJE062_2461 | SBP_bac_1 | sugar ABC transporter, periplasmic sugar-binding protein |  |
|  |  |  |  |  |  |
| PSE_4804 | PJE062_2295 | ABC_tran | High-affinity branched-chain amino acid transport ATP-binding protein BraG | *braG* | **Amino acid** |
| PSE_4805 | PJE062_2276 | ANF_receptor | branched-chain amino acid ABC transporter, periplasmic branched-chain amino acid binding protein |  |
| PSE_4806 | PJE062_2012 | BPD_transp_2 | High-affinity branched-chain amino acid transport system permease protein BraE | *braE* |
| PSE_4807 | PJE062_2240 | BPD_transp_2 | High-affinity branched-chain amino acid transport system permease protein BraD | *braD* |
| PSE_4808 | PJE062_2180 | ABC_tran | High-affinity branched-chain amino acid transport ATP-binding protein BraF | *braF* |
|  |  |  |  |  |  |
| PSE_4831 | PJE062_2307 | SBP_bac_5 | Glutathione-binding protein GsiB | *gsiB* | **Oligopeptide** |
| PSE_4832 | PJE062_2293 | BPD_transp_1 | Dipeptide transport system permease protein DppB | *dppB* |
| PSE_4833 | PJE062_2270 | BPD_transp_1 | Dipeptide transport system permease protein DppC | *dppC* |
| PSE_4834 | PJE062_2181 | ABC_tran | Glutathione import ATP-binding protein GsiA | *gsiA* |
|  |  |  |  |  |  |
|  |  |  |  |  |  |
| **Table S 4.** Continued |  |  |  |  |  |
| **Locus  FO-BEG1** | **Locus  JE062** | **Pfam Model** | **Product** | **Gene** | **Predicted substrate specificity** |
| PSE_4895 | PJE062_2134 | ABC_tran | amino-acid ABC transporter ATP-binding protein YecC | *yecC* | **Amino acid** |
| PSE_4896 | PJE062_2061 | BPD_transp_1 | amino-acid ABC transporter permease protein PatM | *patM* |
| PSE_4897 | PJE062_2075 | SBP_bac_3 | amino-acid ABC transporter-binding protein PatH | *patH* |
|  |  |  |  |  |  |
| PSE_4935 | PJE062_2212 | ABC_tran | Molybdenum import ATP-binding protein ModC | *modC* | **Molybdenum** |
| PSE_4936 | PJE062_2256 | BPD_transp_1 | Molybdenum transport system permease protein ModB | *modB* |
| PSE_4937 | PJE062_2348 | SBP_bac_1 | Molybdate-binding periplasmic protein | *modA* |
|  |  |  |  |  |  |
| PSE_4960 | PJE062_2103 | BioY | BioY protein | *bioY* | **Cobalt** |
| PSE_4961 | PJE062_2002 | CbiQ | Cobalt transport protein | *cbiQ* |
| PSE_4962 | PJE062_2254 | ABC_tran | Cobalt import ATP-binding protein CbiO 1 | *cbiO* |
|  |  |  |  |  |  |
| PSE_5060 | PJE062_4022 | SBP_bac_1 | ABC-type sugar transport system, periplasmic component |  | **Glycerol-3-phosphate** |
| PSE_5062 | PJE062_4017 | BPD_transp_1 | sn-glycerol-3-phosphate transport system permease protein UgpE | *ugpE* |
| PSE_5063 | PJE062_3999 | BPD_transp_1 | sn-glycerol-3-phosphate transport system permease protein UgpA | *ugpA* |
| PSE_5064 | PJE062_3997 | ABC_tran | sn-glycerol-3-phosphate import ATP-binding protein UgpC | *ugpC* |
| PSE_5065 | PJE062_4020 | ABC_tran | sn-glycerol-3-phosphate import ATP-binding protein UgpC | *ugpC* |
|  |  |  |  |  |  |
| PSE_p0066 | PJE062_3787 | Peripla_BP_1 | D-ribose-binding protein | *rbsB* | **Sugar** |
| PSE_p0067 | PJE062_3695 | ABC_tran | Putative ribose/galactose/methyl galactoside import ATP-binding protein | *rgmG* |
| PSE_p0068 | PJE062_3689 | BPD_transp_2 | Galactoside transport system permease protein MglC | *mglC* |
|  |  |  |  |  |  |
| PSE_p0070 | PJE062_3838 | ABC_tran | sn-glycerol-3-phosphate import ATP-binding protein UgpC | *ugpC* | **Glycerol-3-phosphate** |
| PSE_p0071 | PJE062_3875 | BPD_transp_1 | sn-glycerol-3-phosphate transport system permease protein UgpE | *upgE* |
| PSE_p0072 | PJE062_3669 | BPD_transp_1 | sn-glycerol-3-phosphate transport system permease protein UgpA | *upgA* |
| PSE_p0073 | PJE062_3794 | SBP_bac_1 | sn-glycerol-3-phosphate-binding periplasmic protein UgpB | *upgB* |
|  |  |  |  |  |  |
|  |  |  |  |  |  |
|  |  |  |  |  |  |
|  |  |  |  |  |  |
|  |  |  |  |  |  |
| **Table S 4.** Continued |  |  |  |  |  |
| **Locus  FO-BEG1** | **Locus  JE062** | **Pfam Model** | **Product** | **Gene** | **Predicted substrate specificity** |
| PSE_p0079 | PJE062_3869 | SBP_bac_1 | ABC transporter, periplasmic binding-protein |  | **Sugar** |
| PSE_p0080 | ─ | BPD_transp_1 | Lactose transport system permease protein LacF | *lacF* |
| PSE_p0081 | ─ | BPD_transp_1 | Multiple sugar-binding transport system permease protein MsmG | *msmG* |
| PSE_p0082 | PJE062_3781 | ABC_tran | Maltose/maltodextrin import ATP-binding protein MalK | *malK* |
|  |  |  |  |  |  |
| PSE_p0179 | PJE062_3795 | BPD_transp_1 | Spermidine/putrescine transport system permease protein PotB | *potB* | **Putrescine/ Spermidine** |
| PSE_p0180 | PJE062_3917 | BPD_transp_1 | Putrescine transport system permease protein PotH | *potH* |
| PSE_p0181 | PJE062_3909 | SBP_bac_1 | ABC transporter, periplasmic substrate-binding protein |  |
| PSE_p0182 | PJE062_3834 | ABC_tran | Spermidine/putrescine import ATP-binding protein PotA | *potA* |
|  |  |  |  |  |  |
| PSE_p0206 | PJE062_3717 | ABC_tran | sugar ABC transporter, ATP-binding protein |  | **Sugar** |
| PSE_p0210 | PJE062_3844 | BPD_transp_1 | L-arabinose transport system permease protein AraQ | *araQ* |
| PSE_p0211 | PJE062_3654 | BPD_transp_1 | starch degradation products transport system permease protein AmyD | *amyD* |
| PSE_p0212 | PJE062_3625 | SBP_bac_1 | ABC-type sugar transport system, periplasmic component |  |
|  |  |  |  |  |  |
| PSE_p0237 | PJE062_3796 | ABC_tran | Oligopeptide transport ATP-binding protein AppF | *appF* | **Oligopeptide** |
| PSE_p0238 | PJE062_3619 | BPD_transp_1 | Oligopeptide transport system permease protein AppC | *appC* |
| PSE_p0239 | PJE062_3887 | BPD_transp_1 | Oligopeptide transport system permease protein AppB | *appB* |
| PSE_p0240 | PJE062_3972 | SBP_bac_5 | Periplasmic dipeptide transport protein | *dppA* |
|  |  |  |  |  |  |
| PSE_p0350 | PJE062_3636 | ABC_tran | Oligopeptide transport ATP-binding protein AppF | *appF* | **Oligopeptide** |
| PSE_p0351 | ─ | ABC_tran | Oligopeptide transport ATP-binding protein OppD | *oppD* |
| PSE_p0352 | PJE062_3745 | BPD_transp_1 | Probable D,D-dipeptide transport system permease protein DdpC | *dppC* |
| PSE_p0353 | PJE062_3770 | BPD_transp_1 | Glutathione transport system permease protein GsiC | *gsiC* |
| PSE_p0354 | PJE062_3690 | SBP_bac_5 | Periplasmic dipeptide transport protein | *dppA* |

**Table S 5.** Genes detected in the genomes of *Pseudovibrio* sp. FO-BEG1 and JE062 coding for predicted peptidases/proteases and proteins involved in protection against reactive oxygen species (ROS). Genes that could not be detected in the not closed genome of strain JE062 are indicated with ‘─’. Absence of a gene name or an EC number indicates that no assignment was made due to missing of these parameters for the respective genes.

| **Locus  FO-BEG1** | **Locus  JE062** | **Product** | **Gene** | **EC** |
| --- | --- | --- | --- | --- |
|  |  | **Peptidases /Proteases** |  |  |
| PSE_0001 | PJE062_3184 | Peptidase M23/M37 family protein |  |  |
| PSE_0229 | PJE062_3278 | Peptidase family M48 |  |  |
| PSE_0251 | PJE062_3407 | Peptidase family S41 |  |  |
| PSE_0273 | PJE062_3414 | D-alanyl-D-alanine carboxypeptidase | *dacC* |  |
| PSE_0434 | ─ | Peptidase family M48 |  |  |
| PSE_0464 | ─ | Peptidase M75, Imelysin |  |  |
| PSE_0466 | PJE062_2551 | Peptidase M75, Imelysin |  |  |
| PSE_0494 | PJE062_2791 | Peptidase M22, glycoprotease |  |  |
| PSE_0563 | PJE062_2577 | Pyrrolidone-carboxylate peptidase | *pcp* | 3.4.19.3 |
| PSE_0669 | PJE062_2688 | Peptidase family S49 |  |  |
| PSE_0709 | PJE062_2578 | Peptidase family S41 |  |  |
| PSE_0710 | PJE062_2576 | Peptidase M23 |  |  |
| PSE_0811 | PJE062_2788 | Peptidase family U32 |  |  |
| PSE_0812 | PJE062_2571 | Peptidase family U32 |  |  |
| PSE_0992 | PJE062_2365 | Penicillin-insensitive murein endopeptidase precursor/D-alanyl-D-alanine-endopeptidase | *mepA* | 3.4.24.- |
| PSE_1070 | PJE062_4381 | Type IV leader peptidase family |  |  |
| PSE_1080 | PJE062_4511 | Cytosol aminopeptidase/leucine aminopeptidase | *pepA* | 3.4.11.1 |
| PSE_1106 | PJE062_4512 | Peptidase M19, renal dipeptidase |  |  |
| PSE_1182 | ─ | Dipeptidase 1/Microsomal dipeptidase |  |  |
| PSE_1188 | ─ | Metallopeptidase family M24 |  |  |
| PSE_1495 | PJE062_5268 | Peptidase M16 family protein |  |  |
| PSE_1496 | PJE062_5323 | Peptidase M16 family protein |  |  |
| PSE_1498 | PJE062_5212 | Lipoprotein signal peptidase | *lspA* | 3.4.23.36 |
| PSE_1533 | PJE062_182 | Peptidase U62, modulator of DNA gyrase |  |  |
| PSE_1565 | PJE062_241 | Peptidase M16 family protein |  |  |
| PSE_1568 | PJE062_410 | D-alanyl-D-alanine carboxypeptidase |  |  |
| PSE_1618 | PJE062_92 | Peptidase family S49 |  |  |
| PSE_1833 | ─ | Peptidase M24 |  |  |
| PSE_1842 | ─ | Peptidase M24 |  |  |
| PSE_2257 | PJE062_671 | Peptidase M23B |  |  |
| PSE_2285 | PJE062_817 | Peptidase family M20/M25/M40 |  |  |
| PSE_2356 | PJE062_223 | Peptidase family M20/M25/M40 |  |  |
| PSE_2396 | PJE062_57 | Peptidase M19, renal dipeptidase |  |  |
| PSE_2422 | PJE062_398 | Peptidase family M20/M25/M40 |  |  |
| PSE_2435 | PJE062_293 | Protease 2/Oligopeptidase B | *ptrB* | 3.4.21.83 |
| PSE_2461 | PJE062_207 | Peptidase U35, phage prohead HK97 |  |  |
| PSE_2472 | PJE062_117 | Phage cell wall peptidase, NlpC/P60 |  |  |
| PSE_2490 | PJE062_382 | Aminopeptidase N (Alpha-aminoacylpeptide hydrolase) | *ampN* | 3.4.11.2 |
| PSE_2733 | PJE062_4294 | Cytosol aminopeptidase/Leucine aminopeptidase | *ampA* | 3.4.11.1 |
|  |  |  |  |  |
|  |  |  |  |  |
| **Table S 5.** Continued |  |  |  |  |
| **Locus  FO-BEG1** | **Locus  JE062** | **Product** | **Gene** | **EC** |
| PSE_2795 | PJE062_1383 | Peptidase family M20/M25/M40 |  |  |
| PSE_2941 | PJE062_1153 | Peptidase family M20/M25/M40 |  |  |
| PSE_2997 | PJE062_1623 | Prolyl oligopeptidase family |  |  |
| PSE_3040 | ─ | Clp protease |  |  |
| PSE_3062 | ─ | Peptidase M15 |  |  |
| PSE_3141 | PJE062_1066 | D-alanyl-D-alanine carboxypeptidase | *dacA* | 3.4.16.4 |
| PSE_3234 | PJE062_1678 | Peptidase M23B |  |  |
| PSE_3404 | PJE062_1716 | Peptidase family M50 |  | 3.4.24.- |
| PSE_3468 | PJE062_1772 | Peptidase M23B |  |  |
| PSE_3475 | ─ | Peptidase family M48 |  |  |
| PSE_3524 | ─ | Peptidyl-dipeptidase dcp (Dipeptidyl carboxypeptidase) | *dcp* | 3.4.15.5 |
| PSE_3609 | PJE062_1430 | Signal peptidase I | *lepB* | 3.4.21.89 |
| PSE_3808 | PJE062_3548 | D-alanyl-D-alanine carboxypeptidase | *dacF* | 3.4.16.4 |
| PSE_3821 | ─ | Clp protease |  | 3.4.21.92 |
| PSE_3979 | PJE062_5150 | Transglutaminase-like cysteine peptidase |  |  |
| PSE_3998 | PJE062_4868 | Methionine aminopeptidase (MAP) (Peptidase M) | *map* | 3.4.11.18 |
| PSE_4028 | PJE062_4576 | Peptidase S58, DmpA |  |  |
| PSE_4247 | PJE062_5063 | Xaa-Pro aminopeptidase 1 | *xpp1* | 3.4.11.9 |
| PSE_4285 | PJE062_5046 | Peptidase T | *pepT* | 3.4.11.4 |
| PSE_4388 | PJE062_5031 | Prolyl oligopeptidase family |  |  |
| PSE_4435 | PJE062_4791 | Xaa-Pro dipeptidase | *pepQ* | 3.4.13.9 |
| PSE_4669 | PJE062_2225 | M42 glutamyl aminopeptidase |  |  |
| PSE_4674 | PJE062_2053 | Cell division protease FtsH homolog | *ftsH* | 3.6.4.3 |
| PSE_4759 | PJE062_2089 | Oligoendopeptidase F homolog |  |  |
| PSE_4873 | PJE062_2076 | Peptidase family M48 |  |  |
| PSE_4884 | PJE062_2338 | O-sialoglycoprotein endopeptidase (Glycoprotease) | *gcp* | 3.4.24.57 |
| PSE_4893 | PJE062_1994 | Peptidase family M20/M25/M40 |  |  |
| PSE_4926 | PJE062_2084 | Peptidase U32 |  |  |
| PSE_5035 | PJE062_4092 | Peptidase S58, DmpA |  |  |
| PSE_0055 | PJE062_3479 | CAAX protease family protein |  |  |
| PSE_0097 | PJE062_3209 | ATP-dependent protease La, LON |  |  |
| PSE_0489 | PJE062_2770 | ATP-dependent protease peptidase subunit | *hslV* | 3.4.25.- |
| PSE_0491 | PJE062_2610 | ATP-dependent hsl protease ATP-binding subunit HslU | *hslU* |  |
| PSE_0494 | PJE062_2791 | Peptidase M22, glycoprotease |  |  |
| PSE_0709 | PJE062_2578 | Peptidase family S41 |  |  |
| PSE_0869 | PJE062_2969 | Serralysin |  | 3.4.24.40 |
| PSE_0880 | PJE062_3017 | zinc protease pqqL | *pqqL* | 3.4.99.- |
| PSE_2435 | PJE062_293 | Protease 2/Oligopeptidase B | *ptrB* | 3.4.21.83 |
| PSE_2484 | PJE062_401 | Serine protease | *degP1* | 3.4.21.- |
| PSE_2726 | PJE062_4115 | Serine protease |  |  |
| PSE_3446 | PJE062_1826 | ATP-dependent protease La | *lon* | 3.4.21.53 |
| PSE_3447 | PJE062_1775 | ATP-dependent Clp protease ATP-binding subunit clpX | *clpX* |  |
| PSE_3448 | PJE062_1602 | ATP-dependent Clp protease proteolytic subunit (Endopeptidase Clp) | *clpP* | 3.4.21.92 |
| PSE_3805 | PJE062_3583 | ATP-dependent Clp protease ATP-binding subunit clpA | *clpA* |  |
|  |  |  |  |  |
|  |  |  |  |  |
| **Table S 5.** Continued |  |  |  |  |
| **Locus  FO-BEG1** | **Locus  JE062** | **Product** | **Gene** | **EC** |
| PSE_3806 | PJE062_3584 | ATP-dependent Clp protease adapter protein clpS 1 | *ClpS* |  |
| PSE_3852 | PJE062_4839 | Serine protease |  |  |
| PSE_4208 | PJE062_5075 | Intracellular protease 1 (Intracellular protease I) | *pfpI* |  |
| PSE_4228 | PJE062_4726 | Trypsin-like serine protease |  |  |
| PSE_4718 | PJE062_1984 | Zinc protease |  |  |
| PSE_4884 | PJE062_2338 | O-sialoglycoprotein endopeptidase (Glycoprotease) | *gcp* | 3.4.24.57 |
|  |  |  |  |  |
|  |  | **Protection against reactive oxygen species** |  |  |
| PSE_0143 | PJE062_3455 | Copper/zinc superoxide dismutase | *sodC* | 1.15.1.1 |
| PSE_2428 | PJE062_90 | Manganese/iron superoxide dismutase | *sodF* | 1.15.1.1 |
|  |  | Catalase |  |  |
| PSE_1899 | PJE062_693 | Peroxidase/catalase (Catalase-peroxidase) | *catA* | 1.11.1.6 |
| PSE_4005 | PJE062_4983 | Catalase | *catA* | 1.11.1.6 |
| PSE_0100 | PJE062_3343 | Cytochrome c551 peroxidase (Cytochrome c peroxidase) (CCP) | *ccpR* |  |
| PSE_0180 | PJE062_3475 | Thioredoxin peroxidase | *tdx* |  |
| PSE_0181 | PJE062_3303 | Alkylhydroperoxidase AhpD | *ahpD* |  |
| PSE_0722 | ─ | Peroxiredoxin, OsmC-like protein | *osmC* |  |
| PSE_1918 | PJE062_770 | Alkyl hydroperoxide reductase/ Thiol specific antioxidant/ Mal allergen |  |  |
| PSE_1949 | PJE062_923 | Di-heme cytochrome c peroxidase |  |  |
| PSE_2025 | PJE062_566 | Alkylhydroperoxidase AhpD |  |  |
| PSE_2156 | PJE062_603 | Alkyl hydroperoxide reductase/ Thiol specific antioxidant/ Mal allergen |  |  |
| PSE_2209 | PJE062_579 | Alkyl hydroperoxide reductase/ Thiol specific antioxidant/ Mal allergen |  |  |
| PSE_2511 | PJE062_470 | Alkyl hydroperoxide reductase/ Thiol specific antioxidant/ Mal allergen |  |  |
| PSE_2926 | PJE062_1164 | Di-heme cytochrome c peroxidase |  |  |
| PSE_3314 | ─ | Alkyl hydroperoxide reductase/ Thiol specific antioxidant/ Mal allergen |  |  |
| PSE_3466 | PJE062_1662 | Alkyl hydroperoxide reductase/ Thiol specific antioxidant/ Mal allergen |  |  |
| PSE_3521 | PJE062_1626 | Phosphatidic acid phosphatase type 2/haloperoxidase |  |  |
| PSE_3918 | ─ | Alkyl hydroperoxide reductase/ Thiol specific antioxidant/ Mal allergen |  |  |
| PSE_4045 | PJE062_4776 | Alkyl hydroperoxide reductase/ Thiol specific antioxidant/ Mal allergen |  |  |
| PSE_4466 | PJE062_4550 | Organic hydroperoxide resistance protein |  |  |
| PSE_5045 | PJE062_4053 | Di-heme cytochrome c peroxidase |  |  |
| PSE_5046 | PJE062_4061 | Di-heme cytochrome c peroxidase |  |  |
| PSE_p0159 | PJE062_3672 | Di-heme cytochrome c peroxidase |  |  |
| PSE_p0165 | PJE062_3748 | Alkylhydroperoxidase AhpD |  |  |
| PSE_p0321 | PJE062_3655 | Alkylhydroperoxidase AhpD |  |  |
|  |  |  |  |  |
|  |  |  |  |  |
|  |  |  |  |  |
|  |  |  |  |  |
|  |  |  |  |  |
|  |  |  |  |  |

**Table S 6.** Genes detected in the genomes of *Pseudovibrio* sp. FO-BEG1 and JE062 coding for predicted proteins involved vitamin synthesis. Genes that could not be detected in the not closed genome of strain JE062 are indicated with ‘─’. Absence of a gene name or an EC number indicates that no assignment was made due to missing of these parameters for the respective genes.

| **Locus  FO-BEG1** | **Locus  JE062** | **Product** | **Gene** | **EC** |
| --- | --- | --- | --- | --- |
|  |  | **Vitamin B1 Thiamine^a)^** |  |  |
| PSE_0621 | PJE062_2616 | Thiamine-phosphate pyrophosphorylase | *thiE* | 2.5.1.3 |
| PSE_0622 | PJE062_2638 | Hydroxyethylthiazole kinase | *thiM* | 2.7.1.50 |
| PSE_0906 | PJE062_2968 | Thiamine biosynthesis protein ThiF | *thiF* | 2.7.7.- |
| PSE_0907 | PJE062_2761 | Thiamine-phosphate pyrophosphorylase | *thiE* | 2.5.1.3 |
| PSE_0908 | PJE062_2880 | Thiazole biosynthesis protein ThiG | *thiG* |  |
| PSE_0909 | PJE062_2900 | ThiS, thiamine-biosynthesis | *thiS* |  |
| PSE_0910 | PJE062_2986 | Thiamine biosynthesis oxidoreductase ThiO | *thiO* | 1.4.3.19 |
| PSE_0911 | PJE062_2936 | Thiamine biosynthesis protein ThiC | *thiC* |  |
| PSE_3819 | PJE062_5088 | Thiamine-phosphate pyrophosphorylase | *thiE* | 2.5.1.3 |
| PSE_4137 | PJE062_5017 | Phosphomethylpyrimidine kinase | *thiD* | 2.7.4.7 |
| PSE_4138 | PJE062_4585 | Hydroxyethylthiazole kinase | *thiM* | 2.7.1.50 |
| PSE_4699 | PJE062_2332 | Thiamine-phosphate pyrophosphorylase | *thiE* | 2.5.1.3 |
| PSE_1900 | PJE062_4447 | Thiamine-binding periplasmic protein | *thiB* |  |
| PSE_1901 | PJE062_4491 | Thiamine transport system permease protein ThiP | *thiP* |  |
| PSE_1902 | PJE062_4514 | Thiamine import ATP-binding protein ThiQ | *thiQ* |  |
| PSE_3582 | PJE062_1837 | Thiamine-monophosphate kinase | *thiL* |  |
| PSE_2998 | PJE062_1279 | Cysteine desulfurase, NifS | *nifS/iscS* | 2.8.1.7 |
| PSE_1658 | PJE062_60 | 1-deoxy-D-xylulose-5-phosphate synthase | *dxpS* | 2.2.1.7 |
| PSE_3005 | PJE062_1473 | TENA/THI-4/PQQC family | *tenA* | 3.5.99.2 |
| PSE_4898 | PJE062_2346 | TENA/THI-4/PQQC family | *tenA* | 3.5.99.2 |
|  |  |  |  |  |
|  |  | **Vitamin B2 Riboflavin^b)^** |  |  |
| PSE_1646 | PJE062_256 | Riboflavin biosynthesis protein RibBA (3,4-dihydroxy-2-butanone 4-phosphate synthase/GTP cyclohydrolase-2) | *ribBA* | 3.5.4.25/ 4.1.99.12 |
| PSE_0835 | PJE062_3009 | Riboflavin biosynthesis protein RibD (Diaminohydroxyphosphoribosylaminopyrimidine deaminase/5-amino-6-(5-phosphoribosylamino)uracil reductase) | *ribD* | 3.5.4.26/ 1.1.1.93 |
| PSE_3584 | PJE062_1363 | Riboflavin synthase beta chain | *risB* |  |
| PSE_3585 | ─ | Riboflavin synthase alpha chain | *risA* | 2.5.1.9 |
| PSE_3586 | PJE062_977 | Riboflavin biosynthesis protein RibD (Diaminohydroxyphosphoribosylaminopyrimidine deaminase/5-amino-6-(5-phosphoribosylamino)uracil reductase) | *ribD* | 3.5.4.26/ 1.1.1.193 |
| PSE_1503 | PJE062_5201 | Riboflavin biosynthesis protein ribF (Riboflavin kinase/ FMN adenylyltransferase) | *ribF* | 2.7.1.26/ 2.7.7.2 |
|  |  |  |  |  |
|  |  | **Vitamin B9 Folic acid^c)^** |  |  |
| PSE_0236 | PJE062_3408 | GTP cyclohydrolase 1 | *folE* | 3.5.4.16 |
| PSE_3972 | PJE062_4864 | GTP cyclohydrolase 1 | *folE* | 3.5.4.16 |
| PSE_2813 | PJE062_994 | Alkaline phosphatase |  | 3.1.3.1 |
| PSE_5052 | PJE062_3977 | Alkaline phosphatase |  | 3.1.3.1 |
| PSE_3836 | PJE062_4956 | Dihydroneopterin aldolase (DHNA) | *folB* | 4.1.2.25 |
|  |  |  |  |  |
|  |  |  |  |  |
| **Table S 6.** Continued |  |  |  |  |
| **Locus  FO-BEG1** | **Locus  JE062** | **Product** | **Gene** | **EC** |
| PSE_0133 | PJE062_3459 | 2-amino-4-hydroxy-6-hydroxymethyldihydropteridine pyrophosphokinase | *folK* | 2.7.6.3 |
| PSE_0134 | PJE062_3328 | Dihydropteroate synthase (DHPS) | *folP* | 2.5.1.15 |
| PSE_0339 | PJE062_3466 | Bifunctional protein folC (Folylpolyglutamate synthase/Dihydrofolate synthase) | *folC* | 6.3.2.12/ 6.3.2.17 |
| PSE_4234 | ─ | Dihydrofolate reductase type III DHFR | *folA* | 1.5.1.3 |
|  |  |  |  |  |
|  |  | **Vitamin B12 Cobalamin^d)^** |  |  |
| PSE_0073 | PJE062_3117 | Aerobic cobaltochelatase subunit CobS | *cobS* | 6.6.1.2 |
| PSE_0074 | PJE062_3423 | Aerobic cobaltochelatase subunit CobT | *cobT* | 6.6.1.2 |
| PSE_0272 | PJE062_3130 | Cobalamin (vitamin B12) biosynthesis CobW-like | *cobW* |  |
| PSE_0298 | PJE062_3171 | Cobyrinic acid a,c-diamide synthase |  |  |
| PSE_0823 | PJE062_2890 | Sirohydrochlorin cobaltochelatase (CbiX) | *cbiX* | 4.99.1.3 |
| PSE_0824 | PJE062_2592 | Precorrin-8X methylmutase/Precorrin isomerase CobH/CbiC | *cobH/cbiC* | 1.7.7.1/ 5.4.1.2 |
| PSE_0825 | PJE062_2904 | Precorrin-6Y C(5,15)-methyltransferase CobL/CbiE/CbiT | *cobL/cbiE/cbiT* | 2.1.1.132 |
| PSE_0826 | PJE062_3055 | Precorrin-2 C(20)-methyltransferase CobI/CbiL | *cobI/cbiL* | 2.1.1.130 |
| PSE_0827 | PJE062_3024 | Precorrin-3B C(17)-methyltransferase CobJ/CibH/GbiG | *cobJ/cibH/cbiG* | 2.1.1.131 |
| PSE_0828 | PJE062_2713 | Precorrin-4 C(11)-methyltransferase CobM/CbiF | *cobM/cbiF* | 2.1.1.133 |
| PSE_0829 | PJE062_2999 | Cobyrinic acid A,C-diamide synthase CobB/CbiA | *cobB/cbiA* | 6.3.5.9 |
| PSE_0830 | PJE062_2731 | Uroporphyrinogen-III C-methyltransferase | *cobA* | 2.1.1.107 |
| PSE_0832 | PJE062_2996 | Cobalt-precorrin-6A synthase | *cbiD* |  |
| PSE_0833 | PJE062_2650 | Precorrin-6A reductase CobK/CbiJ | *cobK/cbiJ* | 1.3.1.54 |
| PSE_2555 | PJE062_4304 | Cobalamin (5'-phosphate) synthase CobS/CobV | *cobS/cobV* | 2.7.8.26 |
| PSE_2556 | PJE062_4127 | Nicotinate-nucleotide-dimethylbenzimidazole phosphoribosyltransferase (CobT/CobU) | *cobT/cobU* | 2.4.2.21 |
| PSE_3492 | PJE062_1509 | Cobalamin biosynthesis protein CobD/CbiB | *cobD/cbiB* | 6.3.1.10 |
| PSE_3493 | PJE062_1793 | Cobyric acid synthase CobQ/CobB | *cobQ/cobB* | 6.3.5.10 |
| PSE_3494 | PJE062_1191 | Cob(I)yrinic acid a,c-diamide adenosyltransferase (CobA/CobO) | *cobA/cobO* | 2.5.1.17 |
| PSE_3495 | PJE062_1559 | Aerobic cobaltochelatase subunit cobN | *cobN* | 6.6.1.2 |
| PSE_3496 | PJE062_1259 | Cobalamin (vitamin B12) biosynthesis CobW | *cobW* |  |
| PSE_3497 | PJE062_1011 | Bifunctional adenosylcobalamin biosynthesis protein CobP/CobU (Adenosylcobinamide kinase/Adenosylcobinamide-phosphate guanylyltransferase) | *cobP/cobU* | 2.7.1.156/ 2.7.7.62 |
| PSE_4445 | PJE062_4542 | Cob(II)yrinic acid a,c-diamide reductase | *bluB* | 1.16.8.1 |
| PSE_4713 | ─ | Cobyrinic acid a,c-diamide synthase CobQ/CobB | *cobQ/cobB* |  |
| PSE_4818 | PJE062_2023 | Cobalamin (vitamin B12) biosynthesis CobW | *cobW* |  |
| PSE_3491 | PJE062_1667 | Threonine-phosphate decarboxylase CobC/CobD | *cobC/cobD* | 4.1.1.81 |
|  |  |  |  |  |
|  |  | **Vitamin B6 Pyridoxine^e)^** |  |  |
| PSE_4671 | PJE062_1992 | Phosphoserine aminotransferase (PdxF) | *serC/pdxF* | 2.6.1.52 |
|  |  |  |  |  |
| **Table S 6.** Continued |  |  |  |  |
| **Locus  FO-BEG1** | **Locus  JE062** | **Product** | **Gene** | **EC** |
| PSE_1564 | PJE062_153 | Threonine synthase | *thrC* | 4.2.3.1 |
| PSE_2728 | PJE062_4225 | 4-hydroxythreonine-4-phosphate dehydrogenase | *pdxA* | 1.1.1.262 |
| PSE_4205 | PJE062_4768 | Pyridoxine 5'-phosphate synthase | *pdxJ* | 2.6.99.2 |
| PSE_p0050 | PJE062_3885 | Pyridoxamine kinase | *pdxY* | 2.7.1.35 |
| PSE_0084 | ─ | Pyridoxine kinase/Pyridoxal kinase/Pyridoxamine kinase | *pdxK* | 2.7.1.35 |
| PSE_1669 | PJE062_333 | Pyridoxine/pyridoxamine 5'-phosphate oxidase | *pdxH* | 1.4.3.5 |
| PSE_1658 | PJE062_60 | 1-deoxy-D-xylulose-5-phosphate synthase | *dxpS* | 2.2.1.7 |
|  |  |  |  |  |
|  |  | **Vitamin H Biotin^f)^** |  |  |
| PSE_3083 | PJE062_1792 | Biotin synthase | *bioB* | 2.8.1.6 |
| PSE_3084 | PJE062_1708 | 8-amino-7-oxononanoate synthase | *bioF* | 2.3.1.47 |
| PSE_3085 | PJE062_1407 | Dethiobiotin synthetase | *bioD* | 6.3.3.3 |
| PSE_3086 | PJE062_1813 | Adenosylmethionine-8-amino-7-oxononanoate aminotransferase | *bioA* | 2.6.1.62 |
| PSE_3087 | PJE062_1055 | 3-oxoacyl-[acyl-carrier-protein] synthase 3 | *bioZ* | 2.3.1.180 |
|  |  |  |  |  |
|  |  | **Lipoic acid^g)^** |  |  |
| PSE_3289 | PJE062_975 | Lipoyl synthase | *lipA* | 2.8.1.8 |
| PSE_3828 | PJE062_4856 | Octanoyltransferase | *lipB* | 2.3.1.181 |

### ^a)^ Thiamin (Vitamin B1)

For the biosynthesis of vitamin B_1_, thiazole and pyrimidine must be synthesized via different pathways and then combined to thiamin phosphate (Begl*ey et a*l., 1999; Jurgens*on et a*l., 2009). We identified genes coding for the enzymes of the pyrimidine branch as well as the proteins responsible for the formation of thiazole, including ThiO for the aerobic formation of dehydroglycine as the final step of the thiazole synthesis. We also identified the key enzyme ThiE, performing the linkage of thiazole and pyrimidine to thiamin phosphate and its subsequent phosphorylation to the vitamin thiamin pyrophosphate via ThiL (Jurgens*on et a*l., 2009).

### ^b)^ Riboflavin (Vitamin B2)

Vitamin B_2_ is required for numerous reactions and processes and is produced by plants and microorganisms, but must be taken up by animals (Fischer and Bacher, 2005). We identified the key enzyme GTP cyclohydrolase II, which is required for the conversion of GTP as the first step of the pathway, in the genomes of FO-BEG1 and JE062. Furthermore, riboflavin synthase and lumazine synthase, catalyzing the formation of riboflavin and the re-utilization of the by-product 5-amino-6-ribitylamino-2,4(1*H*,3*H*)-pyrimidinedion, respectively, as well as other enzymes described to be required for this *de novo* synthesis pathway (Fischer and Bacher, 2005), were found in both genomes (in strain JE062, only the alpha subunit of the riboflavin synthase was missing), indicating the capability of both strains to produce vitamin B_2_.

### ^c)^ Folic acid (Vitamin B9)

Tetrahydrofolate, a biochemical derivate of the folic acid, serves as a donor and acceptor of one-carbon units in a number of anabolic and catabolic processes (Bermingham and Derrick, 2002). All enzymes reported to be required for the formation of folate have been detected in strain FO-BEG1 (in JE062, the dehydrofolate reductase is missing), except for DHNE and DHPP. The roles of 7,8-dihydoneopterin triphosphate epimerase (DHNE) and 7,8-dihydroneopterin triphosphate pyrophosphohydrolase (DHPP) are not clarified yet (Bermingham and Derrick, 2002) and it is questionable, whether they at all are involved in the *de novo* synthesis of folic acid. The capability of the strains FO-BEG1 and JE062 to grow without the external supply of vitamins implies that folate is synthesized in these strains.

### ^d)^ Cobalamin (Vitamin B12)

The *de novo* synthesis of cobalamin is restricted to prokaryotes. All other living organisms must take up this vitamin to ensure the function of several enzymes, like the methionine synthase and the methylmalony-CoA mutase (Marte*ns et a*l., 2002). The biosynthesis of vitamin B_12_ is a complex process that requires an array of enzymes. Two pathways exist for the *de novo* synthesis of cobalamin – the aerobic synthesis, in which cobalt is inserted into the molecule via the proteins CobN, CobS and CobT, and the anaerobic pathway with CbiX as the enzyme responsible for the insertion of cobalt. Genes for both pathways are present in the analyzed genomes of *Pseudovibrio* sp. FO-BEG1 and JE062, CobG, however, a key enzyme in the oxygen-dependent pathway, seems to be missing. A cobalamin biosynthesis pathway of similar structure has been reported for the *Roseobacter* clade already, and vitamin B_12_ production has indeed been shown in *Dinoroseobacter* *shibae* DFL12, a symbiont of marine algae (Wagner-Döbl*er et a*l., 2010).

### ^e)^ Pyridoxine (Vitamin B6)

Only prokaryotes and plants can *de novo* synthesize vitamin B_6_. It is a cofactor of over 100 enzymatic reactions, predominantly in the amino acid metabolism (Fitzpatri*ck et a*l., 2007). In both genomes we could identify genes *pdxA, pdxJ* and *dxpS* coding for the key enzymes of the so-called DXP-dependent pathway, which leads to the condensation of the vitamer pyridoxine 5´-phosphate (PNP) (Fitzpatri*ck et a*l., 2007). Furthermore, the synthesized PNP can be interconverted via the salvage pathway, which is completely present in both genomes, into the other vitamer forms of vitamin B_6_ (Moon*ey et a*l., 2009).

### ^f)^ Biotin (Vitamin H)

A complete operon for the synthesis of biotin consisting of *bioB, bioF, bioD, bioA and bioZ* was identified in the genomes of *Pseudovibrio* sp. FO-BEG1 and JE062. The arrangement of the genes is identical with the described operon of *Mesorhizobium* sp. (Sulliv*an et a*l., 2001), a symbiotic alphaproteobacterium found in the soil. This bacterium contains the unique gene *bioZ,* which is proposed to be involved in the pimeloyl-CoA synthesis in the first steps of biotin formation (Guillén-Navar*ro et a*l., 2005; Streit and Entcheva, 2003). Importantly, in *Mesorhizobium*, the operon is functional and has been proven to produce biotin, if expressed.

### ^g)^ Lipoic acid

For the endogenous synthesis of lipoic acid, which is a branch of the fatty acid biosynthesis, LipB and LipA are required, a lipoyl (octanoyl)-transferase and an enzyme catalyzing the sulfur insertion into the molecule, respectively (Booker, 2004). Genes encoding both proteins were identified in both genomes.

### References

Begley TP, Downs DM, Ealick SE, McLafferty FW, Van Loon APGM, Taylor S *et al.* (1999). Thiamin biosynthesis in prokaryotes. *Arch Microbiol* **171:** 293–300.

Bermingham A, Derrick JP. (2002). The folic acid biosynthesis pathway in bacteria: evaluation of potential for antibacterial drug discovery. *BioEssays* **24:** 637–648.

Booker SJ. (2004). Unraveling the pathway of lipoic acid biosynthesis. *Chem Biol* **11:** 10–12.

Fischer M, Bacher A. (2005). Biosynthesis of flavocoenzymes. *Nat Prod Rep* **22:** 324–350.

Fitzpatrick TB, Amrhein N, Kappes B, Macheroux P, Tews I, Raschle T. (2007). Two independent routes of *de novo* vitamin B_6_ biosynthesis: not that different after all. *Biochem J* **407:** 1–13.

Guillén-Navarro K, Encarnación S, Dunn MF. (2005). Biotin biosynthesis, transport and utilization in rhizobia. *FEMS Microbiol Lett* **246:** 1591–1565.

Jurgenson CT, Begley TP, Ealick SE. (2009). The structural and biochemical foundations of thiamin biosynthesis. *Annu Rev Biochem* **78:** 569–603.

Martens JH, Barg H, Warren MJ, Jahn D. (2002). Microbial production of vitamin B_12_. *Appl Microbiol Biotechnol* **58:** 275–285.

Mooney S, Leuendorf JE, Hendrickson C, Hellmann H. (2009). Vitamin B6: a long known compound of surprising complexity. *Molecules* **14:** 329–351.

Streit WR, Entcheva P. (2003). Biotin in microbes, the genes involved in its biosynthesis, its biochemical role and perspectives for biotechnological production. *Appl Microbiol Biotechnol* **61:** 21–31.

Sullivan JT, Brown SD, Yocum RR, Ronson CW. (2001). The *bio* operon on the acquired symbiosis island of *Mesorhizobium* sp. strain R7A includes a novel gene involved in pimeloyl-CoA synthesis. *Microbiol-SGM* **147:** 1315–1322.

Wagner-Döbler I, Ballhausen B, Berger M, Brinkhoff T, Buchholz I, Bunk B *et al.* (2010). The complete genome sequence of the algal symbiont *Dinoroseobacter shibae*: a hitchhiker's guide to life in the sea. *ISME J* **4:** 61–77.

**Table S 7.** Genes detected in the genomes of *Pseudovibrio* sp. FO-BEG1 and JE062 coding for predicted integrases, transposases, subunits of the gene transfer agent (GTA) and genes of the NRPS/PKS cluster as well as the *tdaA-tdaF* locus involved in tropodithietic acid (TDA) synthesis. Genes that could not be detected in the not closed genome of strain JE062 are indicated with ‘─’. Absence of a gene name indicates that no assignment was made due to missing of this parameter for the respective genes. For the NRPS/PKS cluster, sequence similarity to the genes of the colibactin producing *E. coli* CFT073 strain is given.

| **Locus  FO-BEG1** | **Locus  JE062** | **Product** | **Gene** | |
| --- | --- | --- | --- | --- |
|  |  | **GTA** |  | |
| PSE_2457 | PJE062_203 | Phage DNA Packaging Protein |  | |
| PSE_2458 | PJE062_274 | Phage portal protein, HK97 |  | |
| PSE_2459 | PJE062_167 | hypothetical protein |  | |
| PSE_2460 | PJE062_351 | hypothetical protein |  | |
| PSE_2461 | PJE062_207 | Peptidase U35, phage prohead HK97 |  | |
| PSE_2462 | PJE062_422 | Phage major capsid protein, HK97 family |  | |
| PSE_2463 | ─ | Phage conserved hypothetical protein, phiE125 gp8 |  | |
| PSE_2464 | ─ | Phage head-tail adaptor |  | |
| PSE_2465 | PJE062_282 | conserved hypothetical protein |  | |
| PSE_2466 | PJE062_363 | Phage major tail protein, TP901-1 family |  | |
| PSE_2467 | ─ | Gene transfer agent (GTA) like protein |  | |
| PSE_2468 | ─ | Conserved hypothetical phage protein |  | |
| PSE_2469 | PJE062_443 | Phage minor tail protein |  | |
| PSE_2470 | PJE062_201 | Gene transfer agent protein |  | |
| PSE_2471 | PJE062_275 | Phage conserved hypothetical protein BR0599 |  | |
| PSE_2472 | PJE062_117 | Phage cell wall peptidase, NlpC/P60 |  | |
| PSE_2473 | PJE062_155 | Gene transfer agent (GTA) orfg15, like protein |  | |
|  |  |  |  | |
|  |  | **Integrases** |  | |
| PSE_0066 | PJE062_3298 | Phage integrase family |  | |
| PSE_0453 | PJE062_3485 | Phage integrase family |  | |
| PSE_0457 | ─ | Phage integrase family |  | |
| PSE_0623 | PJE062_2786 | Phage integrase family |  | |
| PSE_0748 | PJE062_2951 | Phage integrase family |  | |
| PSE_1819 | ─ | Phage integrase family |  | |
| PSE_3013 | PJE062_3594 | Phage integrase family |  | |
| PSE_3106 | ─ | Phage integrase family |  | |
| PSE_3107 | ─ | Phage integrase family |  | |
| PSE_3108 | ─ | Phage integrase family |  | |
| PSE_3364 | ─ | Phage integrase family |  | |
| PSE_3365 | ─ | Phage integrase family |  | |
| PSE_3929 | PJE062_5158 | Phage integrase family |  | |
| PSE_4169 | PJE062_1148 | Phage integrase family |  | |
| ─ | PJE062_1677 | Phage integrase family |  | |
| ─ | PJE062_1028 | Phage integrase family |  | |
| ─ | PJE062_507 | Phage integrase family |  | |
| ─ | PJE062_4173 | Phage integrase family |  | |
|  |  |  |  | |
|  |  |  |  | |
| **Table S 7.** Continued |  |  |  | |
| **Locus  FO-BEG1** | **Locus  JE062** | **Product** | **Gene** | |
|  |  | **Transposases** |  | |
| PSE_1176 | ─ | ISCc3, transposase OrfA |  | |
| PSE_1177 | ─ | ISCc3, transposase OrfB |  | |
| PSE_1204 | ─ | ISCc3, transposase OrfB |  | |
| PSE_1205 | ─ | ISCc3, transposase OrfA |  | |
| PSE_2059 | ─ | Transposase |  | |
| PSE_2315 | ─ | ISCc3, transposase OrfA |  | |
| PSE_2316 | ─ | ISCc3, transposase OrfB |  | |
| PSE_3120 | ─ | ISCc3, transposase OrfA |  | |
| PSE_3121 | ─ | ISCc3, transposase OrfB |  | |
| PSE_3337 | ─ | ISCc3, transposase OrfA |  | |
| PSE_3338 | ─ | ISCc3, transposase OrfB |  | |
| PSE_3339 | ─ | ISCc3, transposase OrfB |  | |
| PSE_3340 | ─ | Transposon Tn7 transposition protein TnsE | *tnsE* | |
| PSE_3341 | ─ | Transposon Tn7 transposition protein TnsD | *tnsD* | |
| PSE_3342 | ─ | Transposon Tn7 transposition protein TnsC | *tnsC* | |
| PSE_3344 | ─ | Transposon Tn7 transposition protein TnsB | *tnsB* | |
| PSE_3345 | ─ | Transposon Tn7 transposition protein TnsA | *tnsA* | |
| PSE_3361 | ─ | ISCc3, transposase OrfA |  | |
| PSE_3362 | ─ | ISCc3, transposase OrfB |  | |
| PSE_4189 | ─ | Transposase |  | |
| PSE_p0146 | ─ | Transposase IS4 family protein |  | |
| ─ | PJE062_3174 | Transposase |  | |
|  |  |  |  |  |
| **Locus  FO-BEG1** | **Locus  JE062** | **Product** | **Gene** | **Evalue / identity [%] to *E. coli* CFT073** |
|  |  | **NRPS-PKS** |  |  |
| PSE_3317 | ─ | 4'-phosphopantetheinyl transferase | *hetI* | **─** |
| PSE_3318 | ─ | Cadicidin biosynthesis thioesterase |  | 8.0E-56 / 44% |
| PSE_3319 | ─ | Beta-lactamase class C |  | 1.0E-53 / 34% |
| PSE_3320 | ─ | Polyketide synthase |  | 4.0E-103 / 43% |
| PSE_3321 | PJE062_617* | Protein containing DUF214, permase predicted |  | **─** |
| PSE_3322 | PJE062_571* | Protein containing DUF214, permase predicted |  | **─** |
| PSE_3323 | PJE062_611* | Lipoprotein-releasing system ATP-binding protein LolD | *lolD* | **─** |
| PSE_3324 | ─ | Secretion protein HlyD | *hlyD* | **─** |
| PSE_3325 | ─ | Non-ribosomal peptide synthetase |  | 2.0E-145 / 34% |
| PSE_3326 | ─ | Multi antimicrobial extrusion protein MatE |  | 4.0E-78 / 49% |
| PSE_3327 | ─ | Asp-tRNAAsn/Glu-tRNAGln amidotransferase A subunit |  | 2.0E-89 / 43% |
| PSE_3328 | ─ | Peptide synthetase |  | 0.0 / 43% |
| PSE_3329 | ─ | Non-ribosomal peptide synthetase |  | 0.0 / 38% |
| PSE_3330 | ─ | Polyketide synthase |  | 0.0 / 51% |
|  |  |  |  |  |
|  |  |  |  |  |
| **Table S 7.** Continued |  |  |  |  |
| **Locus  FO-BEG1** | **Locus  JE062** | **Product** | **Gene** | **Evalue / identity [%] to *E. coli* CFT073** |
| PSE_3331 | ─ | Non-ribosomal peptide synthase |  | 0.0 / 39% |
| PSE_3332 | ─ | Acyl-CoA dehydrogenase domain protein |  | 3.0E-79 / 50% |
| PSE_3333 | ─ | Hypothetical protein |  | 2.0E-12 / 56% |
| PSE_3334 | ─ | 3-hydroxybutyryl-CoA dehydrogenase | *hbd* | 1.0E-85 / 64% |
| PSE_3335 | ─ | Polyketide synthase |  | 1.0E-178 / 44% |
| PSE_3336 | ─ | Polyketide synthase |  | 0.0 / 40% |
|  |  |  |  |  |
| PSE_p0217 | PJE062_3951 | Type I polyketide synthase |  | **─** |
|  |  |  |  |  |
|  |  | Genes marked with * encode an ABC transporter |  |  |
|  |  |  |  |  |
|  |  | **Genes presumably involved in TDA biosynthesis^a)^** |  |  |
| PSE_2264 | PJE062_934 | Transcriptional regulator, LysR family protein | *tdaA* | **─** |
| PSE_2263 | PJE062_697 | Glutathione S-transferase domain | *tdaB* | **─** |
| PSE_2261 | PJE062_802 | YwfB | *tdaC* | **─** |
| PSE_2260 | PJE062_828 | thioesterase superfamily protein | *tdaD* | **─** |
| PSE_2259 | PJE062_841 | acyl-CoA dehydrogenase domain protein | *tdaE* | **─** |
| PSE_2247 | PJE062_775 | Flavoprotein | *tdaF* | **─** |
| PSE_1790 | PJE062_343 | phenylacetic acid degradation oxidoreductase PaaK | *paaK* | **─** |
| PSE_1788 | PJE062_430 | phenylacetate-CoA oxygenase, PaaI subunit | *paaI* | **─** |
| PSE_1798 | PJE062_190 | Phenylacetate-CoA oxygenase, PaaJ subunit | *paaJ* | **─** |
| PSE_1673 | PJE062_253 | betaC-S lyase | *malY* | **─** |
| PSE_4373 | PJE062_4989 | sulfite oxidase subunit YedY | *tdaH* | **─** |
| PSE_1234 | PJE062_4367 | sulfite reductase (NADPH) protein | *cysI* | **─** |

**^a)^Genes presumably involved in the production of tropodithietic acid (TDA)**

Information about the involved genes was taken from Geng and Belas (2010) and Geng *et al.* (2008)

### References

Geng HF, Belas R. (2010). Expression of tropodithietic acid biosynthesis is controlled by a novel autoinducer. *J Bacteriol* **192:** 4377–4387.

Geng HF, Bruhn JB, Nielsen KF, Gram L, Belas R. (2008). Genetic dissection of tropodithietic acid biosynthesis by marine roseobacters. *Appl Environ Microbiol* **74:** 1535–1545.

**Table S 8.** Genes detected in the genomes of *Pseudovibrio* sp. FO-BEG1 and JE062 coding for predicted proteins presumably involved in attachment, protein-protein interactions and predicted LuxR transcriptional regulators. Genes that could not be detected in the not closed genome of strain JE062 are indicated with ‘─’. For *luxR* homologues, the presence of an amino-terminal autoinducer-binding or response regulatory domain is indicated with a ‘+’. Absence of a gene name or an EC number indicates that no assignment was made due to missing of these parameters for the respective genes.

| **Locus  FO-BEG1** | **Locus  JE062** | **Product** | | | **Gene** | | **EC** |
| --- | --- | --- | --- | --- | --- | --- | --- |
|  |  | **ORFs containing ankyrin repeat domains^a)^** | | |  | |  |
| PSE_0544 | PJE062_2814 | ankyrin repeat protein | | |  | |  |
| PSE_2079 | ─ | ankyrin repeat protein | | |  | |  |
| PSE_3116 | ─ | ankyrin repeat protein | | |  | |  |
|  |  |  | | |  | |  |
|  |  | **ORFs containing tetratricopeptide repeat domains^a)^** | | |  | |  |
| PSE_0098 | PJE062_3465 | Thioredoxin | | |  | |  |
| PSE_0865 | PJE062_3032 | Methyltransferase type 12 | | |  | | 2.1.1.- |
| PSE_1079 | PJE062_4509 | Tetratricopeptide repeat protein | | |  | |  |
| PSE_1686 | PJE062_118 | Tetratricopeptide repeat protein | | |  | |  |
| PSE_2479 | PJE062_323 | Cytochrome c-type biogenesis protein CycH | | | *cycH* | |  |
| PSE_2694 | PJE062_4181 | Tetratricopeptide repeat protein | | |  | |  |
| PSE_3163 | PJE062_1441 | Tetratricopeptide repeat protein | | |  | |  |
| PSE_3476 | ─ | peptidase M48, Ste24p | | |  | |  |
| PSE_3705 | PJE062_1339 | type III secretion system chaperone protein B | | |  | |  |
| PSE_4003 | PJE062_4620 | Tetratricopeptide repeat protein | | |  | |  |
| PSE_4676 | PJE062_2205 | Tol-Pal system YbgF | | |  | |  |
| PSE_4741 | PJE062_2047 | Tetratricopeptide repeat protein | | |  | |  |
| PSE_4770 | PJE062_2250 | Tetratricopeptide repeat protein | | |  | |  |
| PSE_4880 | PJE062_2166 | HemY domain protein | | | *hemY* | |  |
| PSE_p0011 | PJE062_3638 | Methyltransferase type 12 | | |  | | 2.1.1.- |
|  |  |  | | |  | |  |
|  |  | **ORFs containing Sel1 domains^a)^** | | |  | |  |
| PSE_0262 | PJE062_3497 | Sel1 domain protein repeat-containing protein | | |  | |  |
| PSE_1737 | PJE062_108 | Sel1 domain protein repeat-containing protein | | |  | |  |
| PSE_1793 | PJE062_380 | Sel1 domain protein repeat-containing protein | | |  | |  |
| PSE_2549 | PJE062_4185 | Sel1 domain protein repeat-containing protein | | |  | |  |
| PSE_3010 | PJE062_1367 | Sel1 domain protein repeat-containing protein | | |  | |  |
| PSE_4219 | PJE062_4679 | Sel1 domain protein repeat-containing protein | | |  | |  |
| PSE_4221 | PJE062_5026 | Sel1 domain protein repeat-containing protein | | |  | |  |
| PSE_4700 | PJE062_2257 | Sel1 domain protein repeat-containing protein | | |  | |  |
|  |  |  | | |  | |  |
|  |  |  | | |  | |  |
|  |  |  | | |  | |  |
| **Table S 8.** Continued |  |  | | |  | |  |
| **Locus  FO-BEG1** | **Locus  JE062** | **Product** | | | **Gene** | | **EC** |
|  |  | **Invasion associated locus B^a)^** | | |  | |  |
| PSE_1550 | PJE062_305 | Invasion associated locus B | | | *ialB* | |  |
| PSE_1999 | PJE062_658 | Invasion associated locus B | | | *ialB* | |  |
| PSE_2098 | PJE062_650 | Invasion associated locus B | | | *ialB* | |  |
| PSE_2110 | PJE062_550 | Invasion associated locus B | | | *ialB* | |  |
| PSE_3194 | PJE062_1243 | Invasion associated locus B | | | *ialB* | |  |
|  |  |  | | |  | |  |
|  |  | **Genes associated with amyloid production** | | |  | |  |
| PSE_4342 | PJE062_4994 | Curlin associated repeat | | |  | |  |
| PSE_4343 | PJE062_4861 | Curlin associated repeat | | |  | |  |
| PSE_4344 | PJE062_5083 | Curlin associated repeat | | |  | |  |
| PSE_4345 | PJE062_4741 | Curli production assembly/transport component CsgG | | | *csgG* | |  |
| PSE_4346 | PJE062_4746 | Curli production assembly/transport component CsgF | | | *csgF* | |  |
|  |  |  | | |  | |  |
|  |  | ***tda*/*cpa* locus** | | |  | |  |
| PSE_1070 | PJE062_4381 | Type IV leader peptidase family | | | *cpaA* | |  |
| PSE_1071 | PJE062_4395 | Pilus assembly, Flp-type CpaB | | | *cpaB* | |  |
| PSE_1072 | PJE062_4414 | bacterial type II/III secretion system protein | | | *cpaC* | |  |
| PSE_1074 | PJE062_4427 | pilus assembly protein CpaD | | | *cpaD* | |  |
| PSE_1075 | PJE062_4478 | response regulator receiver protein | | | *cpaE* | |  |
| PSE_1076 | PJE062_4469 | type II/IV secretion system protein, TadA subfamily | | | *cpaF* | |  |
|  |  |  | | |  | |  |
|  |  | **Genes containing YadA domains^a)^** | | |  | |  |
| PSE_2099 | PJE062_871 | protein containing YadA-like, C-terminal domain | | |  | |  |
| PSE_2111 | PJE062_850 | protein containing YadA-like, C-terminal domain | | |  | |  |
|  |  |  | | |  | |  |
|  |  | **Genes containing TadE-like domains^a)^** | | |  | |  |
| PSE_1084 | PJE062_4462 | TadE-like protein | | | *tadE* | |  |
| PSE_1085 | PJE062_4380 | TadE-like protein | | | *tadE* | |  |
|  |  |  | | |  | |  |
| **Locus  FO-BEG1** | **Locus  JE062** | **Product** | **Gene** | **Presence of a response regulatory domain** | | **Presence of an autoinducer domain** | |
|  |  | **LuxR transcriptional regulator** |  |  | |  | |
| PSE_1326 | PJE062_1887 | transcriptional regulator, LuxR family protein | *luxR* | + | |  | |
| PSE_1528 | PJE062_237 | transcriptional regulator, LuxR family protein | *luxR* |  | | + | |
| PSE_1752 | PJE062_454 | transcriptional regulator, LuxR family protein | *luxR* | + | |  | |
| PSE_1756 | PJE062_469 | transcriptional regulator, LuxR family protein | *luxR* | + | |  | |
| PSE_2176 | PJE062_945 | transcriptional regulator, LuxR family protein | *luxR* | + | |  | |
|  |  |  |  |  | |  | |
|  |  |  |  |  | |  | |
| **Table S 8.** Continued |  |  |  |  | |  | |
| **Locus  FO-BEG1** | **Locus  JE062** | **Product** | **Gene** | **Presence of a response regulatory domain** | | **Presence of an autoinducer domain** | |
| PSE_2420 | PJE062_63 | transcriptional regulator, LuxR family protein | *luxR* |  | |  | |
| PSE_4321 | PJE062_5001 | transcriptional regulator, LuxR family protein | *luxR* |  | |  | |
| PSE_4867 | PJE062_2072 | transcriptional regulator, LuxR family protein | *luxR* |  | | + | |
| PSE_4891 | ─ | transcriptional regulator, LuxR family protein | *luxR* |  | |  | |
| PSE_4980 | PJE062_4013 | transcriptional regulator, LuxR family protein | *luxR* |  | |  | |
| PSE_4981 | PJE062_3991 | transcriptional regulator, LuxR family protein | *luxR* |  | |  | |
| PSE_4982 | PJE062_4089 | transcriptional regulator, LuxR family protein | *luxR* |  | |  | |
| PSE_p0305 | PJE062_3930 | transcriptional regulator, LuxR family protein | *luxR* |  | |  | |

### ^a)^ Homologues of genes presumably involved in attachment and prokaryote-eukaryote interactions

Genomes of sponge-associated microorganisms often show an overrepresentation of eukaryotic domains mediating protein-protein interactions, especially ankyrin repeat and tetratricopeptide repeat (TPR) domains containing genes, which are thought to play a role in bacteria-host interactions (L*iu et a*l., 2011; Sie*gl et a*l., 2011; Thom*as et a*l., 2010). Genes containing ankyrin or TPR repeat domains could also be identified in the genome of both *Pseudovibrio* strains. Additionally, we found genes containing YadA and TadE domains, known to be required for binding to host tissue (Hoicz*yk et a*l., 2000) and adherence to surfaces (Kachla*ny et a*l., 2001), respectively. Open reading frames containing Sel1 repeats were detected in both *Pseudovibrio* genomes. Those repeats were found in great abundance in an amoeba symbiont (Schmitz-Ess*er et a*l., 2010) and are thought to mediate prokaryote-eukaryote interactions (Mittl and Schneider-Brachert, 2007). We also identified five homologues of “invasion-associated locus B” genes in genomes of strain FO-BEG1 and JE062. In *Bartonella bacilliformis* the invasion-associated locus B gene (*ialB*) was shown to have a direct role in human erythrocyte parasitism and was needed for adherence and invasion of the erythrocytes by the bacterium (Coleman and Minnick, 2001). In summary, we identified a number of different protein homologues and genes with repeat motifs assumed to be involved in prokaryote-eukaryote interactions, supporting the proposed role of *Pseudovibrio* as a symbiont with possibilities to attach, invade and interact with the host organism.

### References

Coleman SA, Minnick MF. (2001). Establishing a direct role for the *Bartonella bacilliformis* invasion-associated locus B (IalB) protein in human erythrocyte parasitism. *Infect Immun* **69:** 4373–4381.

Hoiczyk E, Roggenkamp A, Reichenbecher M, Lupas A, Heesemann J. (2000). Structure and sequence analysis of *Yersinia* YadA and *Moraxella* UspAs reveal a novel class of adhesins. *EMBO J* **19:** 5989–5999.

Kachlany SC, Planet PJ, DeSalle R, Fine DH, Figurski DH. (2001). Genes for tight adherence of *Actinobacillus actinomycetemcomitans*: from plaque to plague to pond scum. *Trends Microbiol* **9:** 429–437.

Liu MY, Kjelleberg S, Thomas T. (2011). Functional genomic analysis of an uncultured δ-proteobacterium in the sponge *Cymbastela concentrica*. *ISME J* **5:** 427–435.

Mittl PRE, Schneider-Brachert W. (2007). Sel1-like repeat proteins in signal transduction. *Cell Signal* **19:** 20–31.

Schmitz-Esser S, Tischler P, Arnold R, Montanaro J, Wagner M, Rattei T *et al.* (2010). The genome of the amoeba symbiont "*Candidatus* Amoebophilus asiaticus" reveals common mechanisms for host cell interaction among amoeba-associated bacteria. *J Bacteriol* **192:** 1045–1057.

Siegl A, Kamke J, Hochmuth T, Piel J, Richter M, Liang CG *et al.* (2011). Single-cell genomics reveals the lifestyle of *Poribacteria*, a candidate phylum symbiotically associated with marine sponges. *ISME J* **5:** 61–70.

Thomas T, Rusch D, DeMaere MZ, Yung PY, Lewis M, Halpern A *et al.* (2010). Functional genomic signatures of sponge bacteria reveal unique and shared features of symbiosis. *ISME J* **4:** 1557–1567.

**Table S 9.** Genes detected in the genomes of *Pseudovibrio* sp. FO-BEG1 and JE062 coding for predicted subunits of the type III and type VI secretion systems. Genes that could not be detected in the not closed genome of strain JE062 are indicated with ‘─’. Absence of a gene name indicates that no assignment was made due to missing of this parameter for the respective genes. For the type III secretion system, sequence similarity to the best SwissProt hit is given.

| **Locus  FO-BEG1** | **Locus JE062** | **Product** | **Gene** | **Evalue / identity [%] to SwissProt Hit** |
| --- | --- | --- | --- | --- |
|  |  | **Type III secretion system** |  |  |
| PSE_2750 | PJE062_4298 | Inositol phosphate phosphatase IpgD | *ipgD* | 5.0E-18 / 27% |
| PSE_3453 | PJE062_1387 | Protein kinase YpkA | *ypkA* | 5.0E-11 / 26% |
| PSE_3455 | PJE062_1601 | Protein kinase YpkA | *ypkA* | 4.0E-10 / 26% |
| PSE_3456 | PJE062_1751 | Protein kinase YpkA | *ypkA* | 2.0E-12 / 28% |
| PSE_3677 | PJE062_1314 | Low calcium response locus protein D (LcrD) | *yscV* | 0.0 / 54% |
| PSE_3678 | ─ | hypothetical protein |  |  |
| PSE_3679 | PJE062_1029 | dienelactone hydrolase and related enzymes |  |  |
| PSE_3680 | PJE062_1817 | Yop proteins translocation protein U | *yscU* | 4.0E-62 / 41% |
| PSE_3681 | PJE062_1687 | Yop proteins translocation protein T | *yscT* | 8.0E-22 / 38% |
| PSE_3682 | PJE062_1490 | Yop proteins translocation protein S | *yscS* | 2.0E-5 / 52% |
| PSE_3683 | PJE062_1016 | Yop proteins translocation protein R | *yscR* | 8.0E-49 / 57% |
| PSE_3684 | PJE062_1246 | Yop proteins translocation protein Q | *yscQ* | 0.0060 / 30% |
| PSE_3685 | PJE062_1406 | hypothetical protein |  |  |
| PSE_3686 | PJE062_1474 | hypothetical protein |  |  |
| PSE_3687 | PJE062_1036 | Yop proteins secretion ATPase | *yscN* | 2.0E-147 / 66% |
| PSE_3688 | PJE062_1156 | Yop proteins translocation protein L | *yscL* | 8.0E-9 / 26% |
| PSE_3689 | PJE062_996 | hypothetical protein |  |  |
| PSE_3690 | PJE062_1724 | Yop proteins translocation J | *yscJ* | 1.0E-42 / 47% |
| PSE_3691 | PJE062_1825 | hypothetical protein |  |  |
| PSE_3692 | PJE062_1134 | hypothetical protein |  |  |
| PSE_3693 | PJE062_1461 | hypothetical protein |  |  |
| PSE_3694 | PJE062_969 | hypothetical protein |  |  |
| PSE_3695 | PJE062_1844 | hypothetical protein |  |  |
| PSE_3696 | PJE062_1796 | hypothetical protein |  |  |
| PSE_3697 | PJE062_1006 | Yop proteins translocation protein C | *yscC* | 1.0E-8 / 24% |
| PSE_3698 | PJE062_1665 | hypothetical protein |  |  |
| PSE_3699 | PJE062_991 | hypothetical protein |  |  |
| PSE_3700 | PJE062_1656 | hypothetical protein |  |  |
| PSE_3701 | PJE062_1486 | Tetratricopeptide region |  |  |
| PSE_3702 | PJE062_1647 | hypothetical protein |  |  |
| PSE_3703 | PJE062_962 | hypothetical protein |  |  |
| PSE_3704 | PJE062_1171 | hypothetical protein |  |  |
| PSE_3705 | PJE062_1339 | type III secretion system chaperone protein B | *escB* | *1.0E-5 / 27% |
| PSE_3706 | PJE062_1315 | hypothetical protein |  |  |
| PSE_3707 | ─ | hypothetical protein |  |  |
| PSE_3708 | PJE062_1165 | hypothetical protein |  |  |
| PSE_3709 | PJE062_1353 | hypothetical protein |  |  |
| PSE_3710 | ─ | membrane-bound Yop targeting protein YopN | *yopN* | *6.0E-14 / 25% |
|  |  |  |  |  |
|  |  |  |  |  |
|  |  |  |  |  |
| **Table S 9.** Continued |  |  |  |  |
| **Locus  FO-BEG1** | **Locus JE062** | **Product** | **Gene** | **Evalue / identity [%] to SwissProt Hit** |
| PSE_3711 | ─ | hypothetical protein |  |  |
| PSE_3712 | PJE062_1659 | Cyclic nucleotide-binding domain |  |  |
| PSE_3713 | PJE062_1450 | hypothetical protein |  |  |
| PSE_3714 | PJE062_1254 | hypothetical protein |  |  |
| PSE_3715 | PJE062_1816 | Yop proteins translocation protein D | *yscD* | 0.045 / 21% |
| PSE_4190 | ─ | Effector protein YopJ (Virulence factor YopJ) | *yopJ* | 6.0E-22 / 29% |
|  |  |  |  |  |
|  |  | * No SwissProt Hit, BlastP Hit instead |  |  |
|  |  |  |  |  |
|  |  | **Type VI secretion system cluster I** |  |  |
| PSE_1854 | PJE062_773 | ImpA domain protein | *impA* |  |
| PSE_1855 | PJE062_714 | Type VI secretion protein IcmF | *icmF* |  |
| PSE_1856 | PJE062_893 | type VI secretion protein, VC_A0107 family |  |  |
| PSE_1857 | PJE062_506 | type VI secretion protein EvpB | *evpB* |  |
| PSE_1858 | PJE062_730 | type VI secretion system, lysozyme-related protein |  |  |
| PSE_1859 | PJE062_884 | type VI secretion protein, VC_A0110 family |  |  |
| PSE_1860 | PJE062_596 | type VI secretion protein, VC_A0111 family |  |  |
| PSE_1861 | PJE062_492 | FHA domain protein | *fha* |  |
| PSE_1862 | PJE062_933 | type VI secretion lipoprotein, VC_A0113 family |  |  |
| PSE_1863 | PJE062_502 | type VI secretion protein, VC_A0114 family |  |  |
| PSE_1864 | PJE062_588 | type IV / VI secretion system DotU | *dotU* |  |
| PSE_1865 | PJE062_641 | ATPase, type VI secretion system ClpV1 | *clpV1* |  |
|  |  |  |  |  |
|  |  | **Type VI secretion system cluster II** |  |  |
| PSE_2844 | PJE062_1121 | Type VI secretion protein IcmF | *icmF* |  |
| PSE_2845 | PJE062_1271 | Type IV / VI secretion system, DotU/OmpA/MotB | *dotU/ompA/motB* |  |
| PSE_2846 | PJE062_1112 | type VI secretion protein, VC_A0114 family |  |  |
| PSE_2847 | PJE062_1027 | type VI secretion lipoprotein, VC_A0113 family |  |  |
| PSE_2848 | PJE062_1673 | FHA domain protein | *fha* |  |
| PSE_2849 | PJE062_1460 | hypothetical protein |  |  |
| PSE_2850 | PJE062_1178 | hypothetical protein |  |  |
| PSE_2851 | PJE062_1054 | Rhs element Vgr protein |  |  |
| PSE_2852 | PJE062_1408 | hypothetical protein |  |  |
| PSE_2853 | PJE062_1012 | type VI secretion protein, EvpB/VC_A0108 family |  |  |
| PSE_2854 | PJE062_1728 | voltage-gated sodium channel |  |  |
| PSE_2855 | PJE062_1703 | ATPase, type VI secretion system ClpV1 | *clpV1* |  |
| PSE_2856 | PJE062_1167 | type VI secretion protein, VC_A0111 family |  |  |
| PSE_2857 | PJE062_1049 | type VI secretion protein, VC_A0110 family |  |  |
|  |  |  |  |  |
| **Table S 9.** Continued |  |  |  |  |
| **Locus  FO-BEG1** | **Locus JE062** | **Product** | **Gene** | **Evalue / identity [%] to SwissProt Hit** |
| PSE_2858 | PJE062_1774 | type VI secretion system lysozyme-related protein |  |  |
| PSE_2859 | PJE062_1348 | virulence protein, SciE type | *sciE* |  |
| PSE_2860 | PJE062_988 | SciM protein | *sciM* |  |
| PSE_2861 | PJE062_1094 | Cytoplasmic protein SciI | *sciI* |  |
| PSE_2862 | PJE062_1199 | Cytoplasmic protein SciH | *sciH* |  |
| PSE_2863 | PJE062_1550 | ImpA domain protein | *impA* |  |
|  |  |  |  |  |
| PSE_p0119 | ─ | Rhs-family protein | *rhs* |  |
| PSE_p0120 | ─ | hypothetical protein |  |  |
| PSE_p0121 | ─ | FHA domain protein | *fha* |  |
| PSE_p0122 | ─ | type VI secretion system Vgr family protein | *vgr* |  |
| PSE_p0123 | ─ | hypothetical protein |  |  |
| PSE_p0124 | ─ | Type VI secretion system effector, Hcp1 | *hcp1* |  |
|  |  |  |  |  |
| PSE_1192 | PJE062_748 | Type VI secretion system effector, Hcp1 family | *hcp1* |  |
| PSE_1193 | ─ | ypothetical protein |  |  |
| PSE_1194 | ─ | hypothetical protein |  |  |
| PSE_1195 | PJE062_528 | hypothetical protein |  |  |
| PSE_1196 | PJE062_779 | Type VI secretion system Vgr family protein | *vgr* |  |
