# Supplementary material for: The genus Pseudovibrio contains metabolically versatile bacteria adapted for symbiosis
Source: Environ Microbiol. 2013 Apr 18;15(7):2095–113. doi: 10.1111/1462-2920.12123 (PMC3806328; doi:10.1111/1462-2920.12123)
Supplement: Table S10 — Sequences associated with the Roseobacter cluster that have been used for the calculation of the phylogenetic tree. [file emi0015-2095-sd11.doc]

**Table S10.** Sequences associated with the *Roseobacter* cluster that have been used for the calculation of the phylogenetic tree.

| **Sequence name** | **Accession number** |
| --- | --- |
| *Sulfitobacter delicatus* strain KMM 3584T | AY180103 |
| *Sulfitobacter dubius* strain KMM 3554T | AY180102 |
| *Oceanibulbus indolifex* HEL−45 1103467007066 | ABID01000001 |
| *Sulfitobacter litoralis* strain Iso 3 | DQ097527 |
| *Sulfitobacter pontiacus* strain LMG19752 | DQ915637 |
| *Sulfitobacter brevis* | Y16425 |
| *Sulfitobacter mediterraneus* | Y17387 |
| *Sulfitobacter mediterraneus* strain LMG19756 | DQ915636 |
| *Roseobacter denitrificans* | M96746 |
| *Roseobacter litoralis* (ATCC 49566) | X78312 |
| *Loktanella fryxellensis* | AJ582225 |
| *Loktanella salsilacus* | AJ440997 |
| *Loktanella vestfoldensis* | AJ582226 |
| *Loktanella hongkongensis* strain UST950701−009P | AY600300 |
| *Jannaschia helgolandensis* | AJ438157 |
| *Jannaschia rubra* strain 4SM3T | AJ748747 |
| *Jannaschia donghaensis* strain DSW−17 | EF202612 |
| *Oceanicola granulosus* HTCC2516 1099521380021 | AAOT01000030 |
| *Octadecabacter arcticus* 238 | U73725 |
| *Roseobacter gallaeciensis* | Y13244 |
| *Silicibacter pomeroyi* strain DSM15171 | DQ915631 |
| *Silicibacter pomeroyi* strain DSS−3 | AF098491 |
| *Ruegeria lacuscaerulensis* strain ITI−1157 | U77644 |
| *Roseovarius mucosus* | AJ534215 |
| *Roseovarius tolerans* | Y11551 |
| *Roseovarius nubinhibens* ISM 1099451318301 | AALY01000001 |
| *Roseovarius halotolerans* HJ50 | EU431217 |
|  |  |
| Table S10. Continued |  |
| **Sequence name** | **Accession number** |
| *Roseivivax halodurans* | D85829 |
| *Roseivivax halotolerans* | D85831 |
| *Salipiger mucescens* strain A3 | AY527274 |
| *Oceanicola batsensis* HTCC2597 1099451005753 | AAMO01000005 |
| *Antarctobacter heliothermus* | Y11552 |
| *Antarctobacter heliothermus* strain DSM11445 | DQ915602 |
| *Rhodobacter capsulatus* strain ATCC 11166 | DQ342320 |
| *Rhodobacter sphaeroides* | X53854 |
